# An Online Community Improves Adherence in an Internet-Mediated Walking Program. Part 1: Results of a Randomized Controlled Trial

**DOI:** 10.2196/jmir.1338

**Published:** 2010-12-17

**Authors:** Caroline R Richardson, Lorraine R Buis, Adrienne W Janney, David E Goodrich, Ananda Sen, Michael L Hess, Kathleen S Mehari, Laurie A Fortlage, Paul J Resnick, Brian J Zikmund-Fisher, Victor J Strecher, John D Piette

**Affiliations:** ^9^Michigan Diabetes Research and Training CenterUniversity of MichiganAnn Arbor, MIUnited States; ^8^Center for Health Communications ResearchUniversity of MichiganAnn Arbor, MIUnited States; ^7^Department of Health Behavior and Health EducationUniversity of MichiganAnn Arbor, MIUnited States; ^6^Department of Internal MedicineUniversity of MichiganAnn Arbor, MIUnited States; ^5^School of InformationUniversity of MichiganAnn Arbor, MIUnited States; ^4^Department of StatisticsUniversity of MichiganAnn Arbor, MIUnited States; ^3^College of Nursing - Adult HealthWayne State UniversityDetroit, MIUnited States; ^2^Health Services Research & Development Center for Clinical Management ResearchVeterans Affairs Ann Arbor Healthcare SystemAnn Arbor, MIUnited States; ^1^Department of Family MedicineUniversity of MichiganAnn Arbor, MIUnited States

**Keywords:** Internet, walking, social support, chronic disease management, adherence, attrition, retention, engagement, online community, exercise

## Abstract

**Background:**

Approximately half of American adults do not meet recommended physical activity guidelines. Face-to-face lifestyle interventions improve health outcomes but are unlikely to yield population-level improvements because they can be difficult to disseminate, expensive to maintain, and inconvenient for the recipient. In contrast, Internet-based behavior change interventions can be disseminated widely at a lower cost. However, the impact of some Internet-mediated programs is limited by high attrition rates. Online communities that allow participants to communicate with each other by posting and reading messages may decrease participant attrition.

**Objective:**

Our objective was to measure the impact of adding online community features to an Internet-mediated walking program on participant attrition and average daily step counts.

**Methods:**

This randomized controlled trial included sedentary, ambulatory adults who used email regularly and had at least 1 of the following: overweight (body mass index [BMI] ≥ 25), type 2 diabetes, or coronary artery disease. All participants (n = 324) wore enhanced pedometers throughout the 16-week intervention and uploaded step-count data to the study server. Participants could log in to the study website to view graphs of their walking progress, individually-tailored motivational messages, and weekly calculated goals. Participants were randomized to 1 of 2 versions of a Web-based walking program. Those randomized to the “online community” arm could post and read messages with other participants while those randomized to the “no online community" arm could not read or post messages. The main outcome measures were participant attrition and average daily step counts over 16 weeks. Multiple regression analyses assessed the effect of the online community access controlling for age, sex, disease status, BMI, and baseline step counts.

**Results:**

Both arms significantly increased their average daily steps between baseline and the end of the intervention period, but there were no significant differences in increase in step counts between arms using either intention-to-treat or completers analysis. In the intention-to-treat analysis, the average step count increase across both arms was 1888 ± 2400 steps. The percentage of completers was 13% higher in the online community arm than the no online community arm (online community arm, 79%, no online community arm, 66%, *P* = .02). In addition, online community arm participants remained engaged in the program longer than no online community arm participants (hazard ratio = 0.47, 95% CI = 0.25 - 0.90, *P* = .02). Participants with lower baseline social support posted more messages to the online community (*P* < .001) and viewed more posts (*P* < .001) than participants with higher baseline social support.

**Conclusion:**

Adding online community features to an Internet-mediated walking program did not increase average daily step counts but did reduce participant attrition. Participants with low baseline social support used the online community features more than those with high baseline social support. Thus, online communities may be a promising approach to reducing attrition from online health behavior change interventions, particularly in populations with low social support.

**Trial Registration:**

NCT00729040; http://clinicaltrials.gov/ct2/show/NCT00729040 (Archived by WebCite at http://www.webcitation.org/5v1VH3n0A)

## Introduction

Intensive and expensive interventions targeting diet and exercise can reduce the risk of developing chronic conditions such as diabetes and cardiovascular disease [1]. The major challenge that remains is to find a way to deliver lifestyle interventions to more people and at a lower cost. Individuals, health systems, and insurance providers are turning to automated lifestyle interventions as a way to control costs and improve health outcomes. Automated lifestyle interventions assist users with diet and exercise logging, goal setting, feedback, and motivational messages. In addition to being lower cost than interventions delivered by a trained provider, automated interventions can be more convenient for the user in that they do not require frequent travel to a facility or scheduled synchronous sessions. Unfortunately, many of the automated lifestyle interventions that have been tested suffer from high dropout rates [2] and limited effectiveness.

Online communities are groups of users that interact by posting and reading messages on a group message board on the Internet. Online communities have the potential to improve both participant retention and the effectiveness of automated lifestyle interventions [3]. An active online community might contain user posted stories about overcoming barriers, empathic messages of support for those who are struggling, and celebrations of success. Such user interaction, if successful, could leverage social support, positive social modeling, and dynamic content to keep users engaged in the program and to support behavior change.

Unfortunately, previous studies examining the impact of online communities on Internet-mediated lifestyle interventions have been disappointing. In a review of 38 studies of online communities in Internet-mediated health interventions by Eysenbach et al, there was little evidence found of a positive impact of online communities on behavioral outcomes or program retention [4]. One of the major issues limiting the effectiveness of online communities is that it is difficult to create and sustain a vibrant and active online community. In a recent review of online health interventions, Bennet and Glasgow state that "despite our best efforts, forums, message boards, and chat rooms are rarely used in Internet interventions" [5].

The primary goal of this trial was to measure the impact on program retention and behavior change of adding an online community to an automated lifestyle change intervention. We added an online community to an automated Internet-mediated walking program that has been shown in previous studies to increase walking by approximately 1 mile per day among participants [6]. The hypothesis was that participants with access to online community features would increase step counts more and would remain engaged in the program longer than those without online community access.

The online community in this study was designed using strategies and features to encourage participant engagement and to increase the chances that the online community conversation would be active enough to have a measurable impact on users. The focus of this manuscript is to report the main outcomes from the randomized controlled trial. A second manuscript in this issue details the strategies used to create the online community [7].

## Methods

### Study Design

In this 2-arm randomized controlled trial, participants in both the intervention and control arms were enrolled in Stepping Up to Health (SUH), an Internet-mediated walking program. Participants in both arms were given a user name and password that allowed them to access a personalized intervention webpage. Intervention-arm participants in the “online community” arm had access to online community features embedded in their intervention webpage. In contrast, control participants allocated to the ”no online community” arm could not read or post messages to other control-arm participants.

### Recruitment

A list was obtained of all patients who received treatment from a University of Michigan Health System provider within the previous 6 months with at least 1 of the following: body mass index (BMI) ≥ 25, type 2 diabetes, or coronary artery disease. Individuals diagnosed with quadriplegia or paraplegia or as having been pregnant within the previous year were excluded. Using a computerized process [8], a random subsample of the list received an invitation letter (Multimedia Appendix 1) for study participation. The letter included a brief description of the study, key eligibility criteria, and a website address for more information. Individuals who heard about the study by word-of-mouth were referred to the study website for detailed study information and eligibility screening.

### Eligibility Screening and Consent

Interested individuals were instructed to go to the study website where they completed an automated eligibility (Multimedia Appendix 2) and consent (Multimedia Appendix 3) process online. Participants were eligible if they were over 18 years of age and had at least one of the following: BMI ≥ 25, type 2 diabetes, or coronary artery disease. To be eligible, participants had to have access to an Internet-connected computer with Windows XP or Vista operating system, a valid email address, and use email at least once per week. Additionally, participants had to be sedentary, which was defined as less than 150 minutes per week of moderate physical activity [9]. Participants were required to have access to a treating physician who could provide medical clearance. Individuals were not eligible if they were pregnant, could not walk a block on their own, or could not make their own medicolegal decisions.

After providing consent, participants received a mailed packet containing a pedometer, an upload cable for the pedometer, pedometer instructions, study team contact information, and a medical clearance form (Multimedia Appendix 4) for the participant’s physician to complete and return.

### Baseline Data Collection

Baseline data collection had 2 components: survey data and pedometer data. Participants completed a detailed online survey (Multimedia Appendix 5) including questions about demographics, health history, motivations, and barriers for walking, knowledge and attitudes about diabetes, heart disease and obesity, and comfort with computers.

Step counts were assessed using an Omron HJ-720-ITC pedometer that contains a dual-axial accelerometer, an embedded USB port, and enough memory to store 42 days of step-count data. These pedometers are valid and reliable [10] and accurate to ± 4% of observed steps [11]. During the baseline period, pedometer displays were covered by a sticker. Participants wore the pedometer for 7 days without removing the sticker and then uploaded their step-count data.

### Randomization

Once participants completed baseline data collection and submitted a signed medical clearance form, an automated randomization algorithm [12] assigned them to either the control or the intervention arm with unequal probability (a ratio of 1:5). The randomization of more individuals to the intervention arm was intentional to ensure a large participant pool to sustain online community dialogue. This type of unequal randomization has been used in previous studies, often for ethical reasons [13]. Such unequal designs retain all of the benefits of a balanced randomized controlled trial with respect to controlling for potential confounding and do not introduce statistical bias. Total sample size was increased to counteract the decrement to statistical power resulting from unequal randomization (see “Sample Size Calculation” section for details).

Once randomized, participants received automated email messages informing them of their initial step-count goals and instructing them to remove the stickers from their pedometers. Participants then gained full access to their personalized intervention page based on their arm assignment.

### Intervention

**Figure 1:**
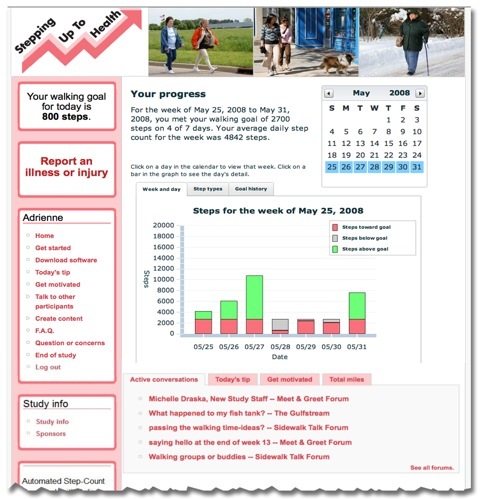
Screenshot of website

The intervention website was implemented in Drupal [14], an open-source content management system with online community features. Figure 1 is a sample screen shot of a personalized SUH home page. The SUH intervention includes 4 intervention components described in detail in a previously published manuscript: uploading pedometers, step-count feedback, individually assigned and gradually incrementing step-count goals, and individually tailored motivational messages [6]. Participants were instructed to wear their pedometers every day while awake and to log in at least once a week to view tailored messages and updated goals.

### Theoretical Framework

The key behavior change theories that support our current hypotheses are Bandura’s social-cognitive theory and social influence theories including social learning theory [15]. There are 3 possible mechanisms by which participation in an online community might impact program attrition and step counts.

#### Mechanism 1: Increased Social Support

Social support, defined as the structure and quality of social relationships, can improve health outcomes by improving adherence to healthy behaviors [16] and by impacting emotions and mood [17-19].

#### Mechanism 2: Social Modeling

The experiences of others, including the barriers they have overcome and the successes they have achieved, can serve as inspirational models. Reading the posts of others enables vicarious learning [20].

#### Mechanism 3: Increased Intervention Website Exposure

Online communities can provide engaging and dynamic content that increase return visits and encourage use of nononline community components including self-regulation components such as goal setting, feedback, and tailored motivational messages.

**Figure 2:**
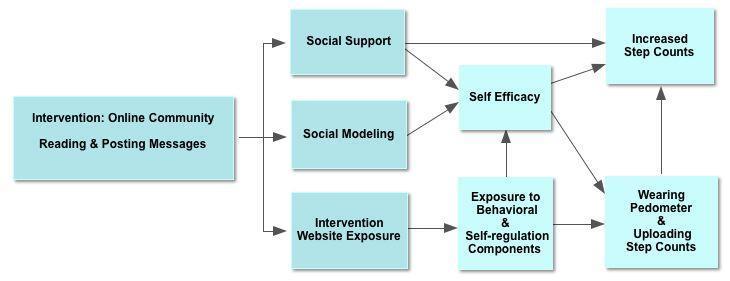
Conceptual model

The online community design followed principles and practices developed by online community experts. Consistent with our theoretical model, content in the online community was focused on providing social support, encouraging social modeling of successes, and facilitating use of noncommunity components of the intervention. To promote sociability, participants were encouraged to post self-introductions, and research staff posted their own self-introductions. In addition, research staff posted open-ended questions encouraging participants to post messages modeling self-regulation strategies such as overcoming barriers and describing successes. Posts about pedometers, goals, and graphs encouraged participants to pay attention to the nononline community components of the intervention. To generate more activity, contests were run with small rewards such as water bottles or bumper stickers for posting content. Because researchers have found that people who get responses, especially to initial posts, are more likely to continue posting, research staff made an effort to post responses, usually within 24 hours, to all participant posts [21-23]. All posts by staff were identified as such. Part 2 of this manuscript provides more details about strategies used to stimulate the online community [7].

### Postintervention Assessment

At the end of the 16-week intervention period, participants completed a postintervention online survey, performed a final pedometer upload, and received a US $25 honorarium plus a free 1-year subscription to a commercial Internet-mediated walking program [24].

### Objective Measures

#### Change in Average Daily Step Counts

Change in average daily step counts was calculated by subtracting average end-of-study step counts from average baseline step counts using uploaded pedometer data. Days during which the pedometer was not worn (less than 100 steps recorded during the day or less than 8 hours of wear time as assessed by the pedometers activity flag) were considered not valid and were not included in averages. At least 5 of 7 consecutive days of valid baseline data were required for randomization. At least 20 of 30 days of valid pedometer data were required to calculate the average step count at the end of intervention period.

#### Percent of Valid Days of Pedometer Data

The percent of valid days of uploaded pedometer data was calculated by dividing the number of valid days of uploaded pedometer data by 112 days (16 weeks).

#### Online Community Use

Each click by a user on a website hyperlink generated a time-stamped record. Each instance of clicking a link that led to a section or subsection of the online community and its features counted as a “view.” Each instance in which a participant composed a new post or replied to an existing message on the online community counted as a “post.”

#### Intervention Completers

Participants who uploaded at least 20 valid days of pedometer data during the final month of the 4-month intervention were considered “completers” for the completers and attrition analysis.

### Subjective Measures

Participants responded to a series of lengthy surveys (Multimedia Appendices 5 through 8). The majority of the survey responses were used only to inform the tailored messaging algorithms (Multimedia Appendices 9 through 16). Self-reported responses to online survey items about age, sex, race, height, weight, Internet proficiency, previous pedometer use, and previous use of social media were also used to describe the study sample and to control for potential confounding in multiple regression. In addition, 2 single-item, unvalidated measures were used as predictors or outcomes in secondary analyses, 1 on social support and 1 on motivation for walking. Social support was measured in the baseline survey with the question, “Do you currently get support from your family and friends in getting enough physical activity?” Additionally, in a brief survey at the end of the intervention period, participants with online community support responded to the question, “Did the ability to talk to or read posts from other participants motivate you to walk more?”

### Statistical Analysis 

#### Sample Size Calculation

In calculating sample size, 2 goals were considered. First, as in a traditional sample size calculation, the sample size was calculated to provide adequate power based on the variance and clinically significant difference of the outcome. The minimum clinically significant increase in average daily step counts was estimated at 1000 steps. If an individual walks with moderate intensity at 3 miles per hour, an increase of 1000 steps is equivalent to approximately 10 minutes of walking per day. A previous study using the SUH intervention revealed a step count standard deviation of 2000 steps [6]. If statistical power had been the only goal in determining sample size, the sample required in each arm would have been 63 for a total sample of 126. However, we also desired a sufficient number of participants in the online community arm to sustain an active online community. To this aim, we increased the total sample size and changed the randomization ratio to yield the appropriate sample size for adequate power with an unequal design. We then increased this estimated sample size by 25% to allow for attrition, and our final total targeted sample size was 300 participants.

#### Analysis

Univariate statistics summarized baseline characteristics and process and outcome variables. Means and standard deviations were reported for continuous variables with a normal distribution, and percentages were reported for categorical variables. Within-arm comparisons between baseline and endpoint physical activity levels used paired *t* tests. For all other results, multiple regression models controlled for the continuous variables, age and BMI, and for the dichotomous variables, sex, type 2 diabetes, and coronary artery disease. All regressions were also adjusted for average baseline step count (a continuous variable) except when the dependent variable was average baseline step count. Regression assumptions were tested and regressions were performed with and without influential outliers to ensure validity. For intention-to-treat analyses, all individuals who were randomized were included in the analysis and baseline values were carried forward for those who did not complete the program. Completers analyses only included individuals who completed the program, uploading at least 20 valid days of pedometer data during the final month of the program.

Linear regression analysis was used with normally distributed, continuous dependent variables including total steps, change in total steps, and valid days uploaded. Logistic regression analysis was used to estimate the effect of online community access on attrition rate and walking motivation. Likert scales were dichotomized for analysis. Poisson regressions compared website variables indicating counts of messages posted and posts viewed and compared the frequency of total, serious, and minor adverse events.

A mixed-model regression compared the rate of step-count increase between arms. A time-to-event analysis compared time to last pedometer upload between arms, with an unadjusted log-rank test for equality of survivor functions and a Cox regression model controlling for confounders with a Breslow methods for ties. Those individuals whose last upload was after 102 days were right censored. STATA 10.1 (StataCorp, College Station, TX, USA) was used for sample size calculations and statistical analyses.

### Human Subjects

The University of Michigan Institutional Review Board approved the study with a waiver of documentation of written consent (IRBMED HUM00012230). All participants gave online consent.

## Results

### Recruitment

**Figure 3:**
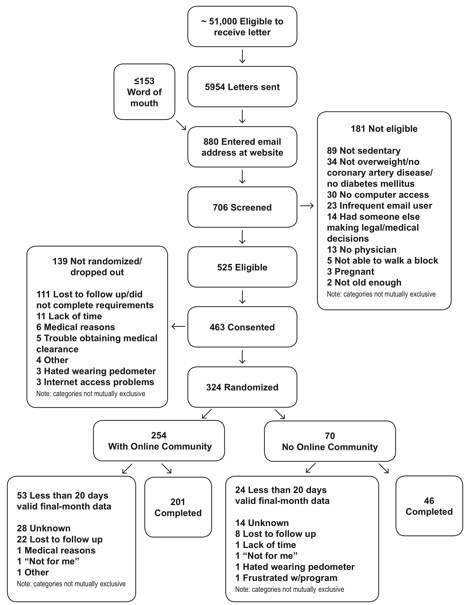
Recruitment flow sheet

A total of 5954 potentially eligible patients received invitation letters. Of those, 706 completed online eligibility screening, and 525 were eligible to participate. A total of 324 individuals completed baseline enrollment procedures (online community arm = 254, no online community arm = 70). See Figure 3 for more details.

### Baseline Characteristics

Participant ages ranged from 24 to 82 years (mean age 52.0 ± 11.4). Two-thirds of participants were women and the majority of participants were white (Table 1). Baseline step counts were significantly higher in the online community arm than the no online community arm. This difference was due to chance alone, as a computer algorithm assigned participants randomly to arms with no research staff input (Table 2).

**Table 1:** Baseline demographics and characteristics by arm^a^

		Both Arms	No Online Community Arm	With Online Community Arm
N		324	70	254
Mean age (SD)		52.0 (11.4)	53.3 (11.8)	51.7 (11.3)
**Gender**				
	Male, %	35%	34%	36%
	Female, %	65%	66%	64%
	Hispanic, %	2%	1%	2%
**Race**				
	White, %	86%	80%	87%
	Black, %	6%	6%	6%
	Asian, %	3%	6%	3%
	American Indian, %	1%	1%	0%
	Other, %	2%	4%	1%
**Body Mass Index**				
	Mean BMI (SD)	33.2 (6.2)	33.4 (5.8)	33.1 (6.3)
	BMI ≥ 25, %	99%	99%	99%
	BMI ≥ 30, %	62%	67%	60%
Coronary artery disease, %		12%	13%	12%
Type 2 diabetes, %		20%	26%	19%
Used pedometer previously, %		43%	41%	44%
**Internet proficiency**				
	Limited, %	2.8%	2.9%	2.8%
	Basic, %	8.8%	8.8%	8.8%
	Moderate, %	33.1%	33.8%	32.9%
	Advanced, %	41.0%	44.1%	39.8%
	Expert, %	14.5%	10.3%	15.7%
**Use social media at least weekly**				
	Forums, %	19.0%	18.3%	19.1%
	Listservs, %	22.6%	25.0%	21.9%
	Chat rooms, %	8.3%	8.3%	8.3%
	Blogs, %	11.5%	6.7%	12.8%

^a^ No significant difference between arms.

### Online Community Use

Consistent with our theoretical model, content in the online community provided social support, encouraged social modeling of successes, and facilitated use of noncommunity components of the intervention. In introductions and elsewhere, many users described personal challenges that made it difficult for them to exercise. This gave participants in the online community arm an opportunity to respond with empathy, encouragement, and informational social support. Both staff and participants referred frequently to nononline community intervention components in posts. Within the online community arm, the online community was active with 65% (165/254) of participants using the online community, either as posters or “lurkers” (ie, readers who did not post).

### Average Daily Step Counts

**Figure 4:**
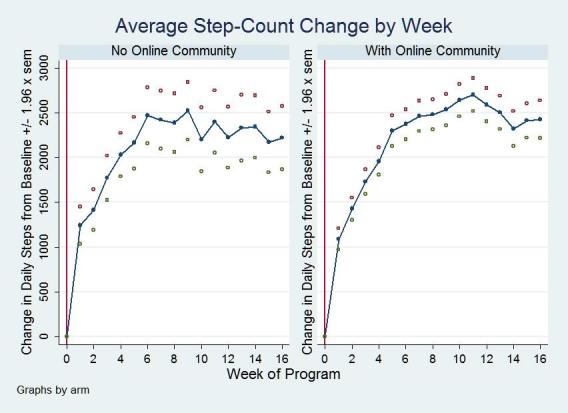
Average step-count change by week

Table 2 shows arm and total sample baseline step counts, final step counts, and absolute change in average daily step counts using both intention-to-treat and completers analysis. Both arms significantly increased their average daily steps between baseline and the end of the intervention period, but there were no significant differences between arms using either intention-to-treat or completers analysis. For the entire sample (n = 324), participants increased their average daily steps by 1888 steps per day in the intention-to-treat analysis (*P* < .001), which approximates to 1 mile per day. Among those who completed the intervention, the average step-count increase was 2477 steps per day (*P* < .001) or about 1.25 miles per day. See Figure 4 for average step-count change by week. The rate of increase in step counts did not differ by arm (*P* = .82).

**Table 2:** Step-count measures by arm

		Both Arms n = 324	No Online Community Arm n = 70	With Online Community Arm n = 254	Between-Arm Comparison *P* value^a^
**Total steps, intention-to-treat**					
	Baseline, mean (SD)	4441 (2000)	3859 (1586)	4601 (2074)	.01
	Final, mean (SD)	6329 (3066)	5438 (2667)	6575 (3127)	.20
	Change, mean (SD)	1888 (2400)	1579 (2137)	1974 (2464)	.20
*P* value (SEM)^b^, intention-to-treat		< .001 (133)	< .001 (255)	< .001 (155)	
Completers, n (% of participants randomized to arm)		247 (76%)	46 (66%)	201 (79%)	
**Total steps, completers**					
	Baseline, mean (SD)	4468 (1884)	4018 (1621)	4571 (1927)	.10
	Final, mean (SD)	6945 (3006)	6421 (2623)	7065 (3081)	.97
	Change, mean (SD)	2477 (2469)	2402 (2232)	2494 (2525)	.97
*P* value (SEM)^b^		< .001 (157)	< .001 (329)	< .001 (178)	

^a^ *P* values for parameter estimate of arm in linear regression adjusting for age, sex, coronary artery disease, type 2 diabetes, BMI, and baseline steps (except where baseline steps was the outcome).

^b^ Pre-post paired *t* tests, not adjusted for confounders

### Program Engagement and Attrition

**Figure 5:**
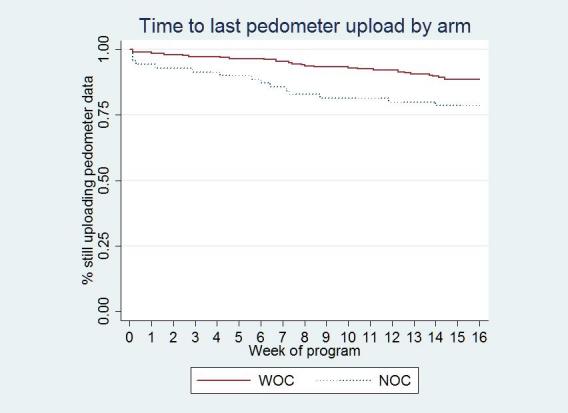
Time-to-event analysis

The online community arm uploaded valid pedometer data on more days than the no online community arm (online community, 87% of days, no online community, 75% of days, *P* = .001). In addition, the online community arm was more likely to upload valid final-month data; percentage of completers was 13% higher in the online community arm than the no online community arm (online community, 79%, no online community, 66%, *P* = .02). Time to last pedometer upload was earlier in the no online community arm indicating that those in the no online community arm dropped out earlier than those in the online community arm (hazard ratio = 0.47*, 95%* confidence interval [CI] = 0.25 - 0.90*, P* = .02). Figure 5 charts the weekly percentage of participants who were still uploading data.

### Social Support

There was no difference between arms in baseline and postintervention perceived social support. Online community arm participants who reported lower baseline social support posted more messages to the online community (incidence-rate ratio = .65, 95% CI = 0.54 - 0.78*, P* < .001,) and viewed more posts (incidence-rate ratio = .50, 95% CI = 0.49 - 0.52*, P* < .001) than participants with higher baseline social support. Participants in both arms who reported having social support at the end of the study were more likely to increase their step counts (*P* = .01).

### Intervention Website Exposure

The online community arm had more home pages hits than the no online community arm with intention-to-treat analysis (*P* = .02) but not with completers analysis. Online community and no online community participants did not differ in views of tailored messages.

### Effect of Online Community Use on Walking

Online community participants who posted more showed a larger increase in step counts (additional 62 steps per day for each message posted, *P* = .03). Additionally, online community participants with more pages viewed had larger increases in step counts (additional 2.3 steps per day per page view, *P* < .001). More posts written and pages viewed correlated with greater reported motivation to increase walking (odds ratio [OR] = 1.15, 95% CI = 1.06 - 1.24*, P* = .001, and OR = 1.005, 95% CI = 1.002 - 1.007*, P* < .001 respectively).

### Adverse Events

There was no difference between arms in the number of related total, serious, or minor adverse events. There were no adverse events associated with online community use. There were 7 serious adverse events related to the intervention including a slip and fall on ice during a walk resulting in a broken leg, 1 hypoglycemic event with a fall, and 5 adverse events related to cardiac symptoms. Minor musculoskeletal injuries were common with 2.5% (8/324) of participants experiencing plantar fasciitis.

## Discussion

### Summary of Principal Results

Access to an online community focused on enhancing social support, social modeling, and self-regulation strategies increased participant retention in an Internet-mediated walking intervention. This study is one of the first to document the benefit of an online community using a randomized controlled trial design. The results presented here strengthen the evidence supporting the use of online communities as a tool for reducing attrition. In contrast, online community access did not change average daily step counts among those who remained in the program. Step-count increases between arms of completers were nearly identical.

While this study was designed to document the overall effect of the online community on program retention and step counts, some secondary quantitative analyses support the mechanisms hypothesized in the conceptual model. First, those who reported lower baseline social support used the online community more frequently both for posting and for reading posts by others. Viewing posts was also correlated with higher increases in step counts. These findings support the hypothesized social support and social modeling mechanisms. The survival curves in Figure 5 suggest that some but not all of the improved retention seen in the online community arm was evident during the first week of participation. This early effect was more likely due to social modeling than to social support as it takes time to build supportive relationships. Posts from participants that modeled overcoming barriers, described personal successes, and gave generic encouragement were available to those in the online community arm from the first time they logged in to the site. Additionally, those in the online community arm used the self-regulation components of the intervention more often than those in the no online community arm. For example, online community arm participants wore their pedometers on more days and uploaded valid pedometer data for more days than no online community participants.

### Study Strengths

There were a number of unique aspects to this study that strengthened the results. First, this study was innovative in that it tested the effect of a single component (online community support) in a randomized controlled trial, the gold standard to determine causality between an intervention and outcome. Randomization minimizes the potential for influence from both measured and unmeasured confounders. The few studies that have assessed the impact of online communities have generally used observational rather than experimental study designs [4]. The effect of online communities detected in such observational studies may be entirely due to confounding constructs such as baseline participant motivation or self-regulation skills. Individuals with baseline traits that favor successful behavior change may be more likely to use online community resources. Additionally, we found support for the hypothesis that online community access would increase exposure to nononline community intervention components such as self-regulation tools. For example, online community participants wore their pedometers on more days and uploaded valid pedometer data for more days than no online community participants.

In addition to randomization, objective measures of outcomes also strengthened the study results. Both walking and program retention outcomes were measured objectively using uploaded pedometer data and electronic logging of participant interaction with the website rather than less reliable subjective reports of retention or behavior changes. Also, the entire intervention as well as all participant recruitment and enrollment procedures were automated and were delivered remotely with no face-to-face interaction between study participants and research staff. This emphasis on automation means that the intervention could be scaled up to a large volume of users with few modifications. Additionally, inclusion criteria were intentionally broad including a large percentage of adults who could benefit from increasing their physical activity. This increases the potential reach of the intervention and strengthens the generalizability of the study results.

### Comparison With Existing Literature

The significant increase in participant retention found in this study contrasts with previously published literature showing no benefit or possible harm from online communities. For example, Glasgow et al found that adding an online community to an information-focused, Internet-based intervention for diabetes self-management did not significantly improve any of the behavioral, biological, or psychosocial outcomes after 10 months compared with the information-only control group [25]. Some studies raise concerns about possible negative effects of online communities. Negative social modeling by online community participants may encourage participants to initiate or continue unhealthy behaviors or negative coping strategies. For example, Takahashi et al studied a peer-support group for depression and found that interactions with individuals who were depressed or had negative perceptions of the online community could trigger depressive states [26].

Consistent with the current findings, a few well-designed randomized controlled trials have shown positive results for Internet-based health behavior interventions. In one study, 580 participants with chronic low-back pain were randomized to an email discussion group or a no email discussion control group. Those randomized to the email discussion intervention group had significant improvements in pain, disability, role function, and health distress compared with the control group [27]. Notably, the email discussion list was active with over 2000 posted messages during the year-long intervention. In fact, this high level of activity may have been detrimental to continued participation; approximately 20% of the intervention-arm participants dropped out specifically because of the high email volume during the first month. In addition to the online community, participants in the intervention arm also received a book and videotape with information about chronic low-back pain, and these confounders may have impacted the improved outcomes. However, the high email volume suggests that the email exchanges played a significant role in improving outcomes.

Previous online community studies have also been limited by low community use. In one of the few trials to specifically examine the impact of an online community, Stoddard et al randomized participants to an online smoking cessation intervention with or without an online community. Of the 684 individuals randomized to the online community intervention, only 81 participants viewed or posted a message [28]. In another randomized study, McKay et al examined the effect of online community features on physical activity among patients with diabetes. Participants randomized to the intervention arm (n = 38) posted only a total of 42 messages during the 8-week intervention. Compared with the control group, participants with online community access had a small and nonsignificant increase in physical activity [29].

A large observational study of a smoking cessation website showed that only 24% of the 607 participants posted messages to the online community, and those who posted had higher quit rates than those who did not post. However, after controlling for use of other online features including interactive quitting tools and one-to-one messaging, the association between posting and increased quit rates was no longer significant. This suggests that the association between online community posting and smoking cessation was not causal and may have been confounded by exposure to other website components or by baseline commitment to quitting [30]. Collectively, these studies reinforce the concern that low online community use is a common problem in automated health behavior change interventions and that low use may weaken the effect of online communities on retention and behavior change outcomes.

### Study Limitations

There are a number of study limitations to consider when interpreting this study. First, by chance and despite randomization, participants in the online community arm were more active at baseline than those in the no online community arm. This difference required control for baseline step counts in all analyses. This was accomplished by using change in step counts as the outcome rather than absolute step counts. Additionally, baseline step counts were included as a potential confounder in all between-arm multiple regression analyses. For future studies, a better approach would use stratified randomization to ensure equitable allocation of higher and lower baseline step-count participants into the two arms.

A second limitation is that the techniques used to stimulate online community involvement required significant research staff contributions to online community content. Such manipulations were necessary to test the effectiveness of an active online community. However, the staff-provided content may differ from spontaneous participant content, so these results might not be generalizable. Size does matter in an online community. Larger online communities tend to have more active interactions and tend to attract and retain more users. Increasing the size of the online community by randomizing more people to the online community arm than to the control arm was another strategy used to insure active and engaging interaction between participants. Studies of large, organic, and preexisting online communities may require less manipulation by research staff to sustain an active conversation, but such studies are difficult to randomize.

A third limitation is that the intervention lasted only 4 months and may not predict attrition and intervention adherence over longer periods. Additionally, there is no information about physical activity level during periods in which the pedometer was not worn. Participants may have been less active on days when they did not wear the pedometer, and this would artificially inflate the calculated average step counts. Because those in the no online community arm uploaded fewer days of valid pedometer data than those in the online community arm, this would bias the results in favor of the no online community arm.

Finally, the association between social support and online community use must be interpreted with caution. The measure of baseline social support was a single-item survey question designed to provide data to a message-tailoring algorithm rather than to precisely measure social support. However, using well-validated measures, previous investigators have established the connection between perceived social support and online community use. Barrera et al randomized participants with diabetes to 1 of 4 conditions: (1) a diabetes information only control, (2) a personal self-management coach, (3) an online community only, or (4) a combination of the personal self-management coach and the online community. Results showed that the online community alone or in combination with the personal self-management coach significantly increased perceived social support compared with the control group [31]. Social support may be a critical component to the success of online interventions, but whether this support actually mediates the relationship between online community use and program engagement remains to be determined. The online communities created in the Barrerra et al study as well as our study were created specifically for the study interventions. An alternative approach may be to create Internet-based health interventions that leverage preexisting friendships and online community affiliations. Building on existing social ties may increase intervention efficacy and is a worthy approach for future studies.

The current study is one in a series that examines the effects of specific components of an Internet-mediated walking program. Previous studies examined components related to participant safety, goal setting options, and group competition on intervention outcomes [6,32,33]. By examining specific components of complex programs, we hope to develop an evidence base that will guide the development of future interventions.

### Conclusions

Adding online community features to an Internet-mediated walking program did not increase participant step count but did reduce attrition. Participants with low baseline social support for physical activity used the online community features more than participants with high baseline social support. Thus, online communities may be one solution to attrition from online health behavior change interventions, particularly in populations with low perceived social support for health behavior change. However, the design and implementation of active online communities is a considerable challenge. Part 2 of this manuscript describes some of the design choices and costs involved in implementing an online community [7].

## Multimedia Appendix 1

Invitation letter

## Multimedia Appendix 2

Eligibility screening

## Multimedia Appendix 3

Informed consent document

## Multimedia Appendix 4

Medical clearance form

## Multimedia Appendix 5

Presurvey (baseline survey)

## Multimedia Appendix 6

Week two survey

## Multimedia Appendix 7

Postsurvey 1

## Multimedia Appendix 8

Postsurvey 2

## Multimedia Appendix 9

Tailored content for session 1

## Multimedia Appendix 10

Tailored content for session 2

## Multimedia Appendix 11

Tailored content for session 3

## Multimedia Appendix 12

Tailored content for session 4

## Multimedia Appendix 13

Tailored content for session 5

## Multimedia Appendix 14

Tailored content for session 6

## Multimedia Appendix 15

Tailored content for session 7

## Multimedia Appendix 16

Tailored content for barriers

## Abbreviations

BMI: body mass index
SUH: Stepping Up to Health
VA: Veterans Affairs
