# Supplementary material for: An Online Community Improves Adherence in an Internet-Mediated Walking Program. Part 1: Results of a Randomized Controlled Trial
Source: J Med Internet Res. 2010 Dec 17;12(4):e71. doi: 10.2196/jmir.1338 (PMC3056526; doi:10.2196/jmir.1338)
Supplement: Supplementary file 1 [file jmir_v12i4e71_app1.pdf]

Step up, and take charge of your health!

Name of Potential Study Participant
Address
City, State, Zip Code

Dear <name of potential study participant>,

Researchers in the Department of Family Medicine at the University of Michigan Health System are recruiting patients for a research study about motivating people to exercise – specifically, to walk. You are invited to participate in this exciting opportunity. Participation involves wearing a pedometer, and using the internet to track your progress for sixteen weeks.

The goal of the study is to learn how to motivate people who do not exercise a lot to increase their exercise gradually, focusing on people who have the most to gain from increasing their exercise level. People who are overweight or live with certain chronic conditions like heart disease or diabetes have the most to gain from walking more.

This opportunity can be of benefit to you in several ways! You'll have the opportunity to improve your own health by getting more exercise, you'll be compensated, and you'll get the chance to take part in important research preventing and managing heart disease and diabetes.

If you are interested in finding out more about this study, visit:

https://www.steppinguptohealth.org

When asked for a "source code," please enter the four-digit number: <**source code**>

If you have trouble with the website, please call us at (734) 998-7120.

Taking part in this research is **voluntary**. You do not have to participate. Your decision on whether to participate will not affect the medical care you receive at the University of Michigan Health System.

Sincerely,
Dr. Caroline Richardson
Dept. of Family Medicine
University of Michigan Health System
