# Supplementary material for: An Online Community Improves Adherence in an Internet-Mediated Walking Program. Part 1: Results of a Randomized Controlled Trial
Source: J Med Internet Res. 2010 Dec 17;12(4):e71. doi: 10.2196/jmir.1338 (PMC3056526; doi:10.2196/jmir.1338)
Supplement: Supplementary file 2 [file jmir_v12i4e71_app2.pdf]

Online eligibility screening questions

What is your date of birth? *

May

16

2008

How tall are you?

How much do you weigh (in pounds)? *

Can you walk at least a block on your own? *

- Yes
- No

Which of the following statements most closely describes you? *

- I do not do any kind of exercise. I try to avoid walking more than a very short distance.
- I do not do any kind of planned exercise, and my daily activities do not include much walking.
- I do not do any kind of planned exercise program, but I walk during my usual daily activities.
- I do medium-intensity exercise, such as going for a brisk walk, one to three days a week.
- I do medium-intensity exercise, such as going for a brisk walk, four or more days a week for at least 30 minutes each time.
- I do high-intensity exercise, like running or step aerobics, at least three times a week for at least 45 minutes each time.

Do you have or have you had any of the following: a diagnosis of coronary artery disease, a heart attack, a stent placed in the arteries in your heart or angina (chest pain related to your heart)? *

- Yes
- No

Do you have type 2 diabetes? *

- Yes
- No

This study will require medical clearance from a physician. Do you have a primary care provider (family doctor), cardiologist (heart specialist) or endocrinologist (diabetes specialist) who can provide medical clearance for you? *

- Yes
- No

Do you have a legal guardian, appointed by a court, who currently makes medical decisions for you? *

- Yes
- No

How often do you check your email? *

- I don't have email
- Every month
- Less than once a week
- Once a week
- Twice a week
- Every other day
- Every day

Do you own a computer, or have regular access to a computer, with an internet connection? *

- Yes
- No

Does that computer have Microsoft Windows XP or Microsoft Windows Vista? *

- Yes
- No
