# Supplementary material for: An Online Community Improves Adherence in an Internet-Mediated Walking Program. Part 1: Results of a Randomized Controlled Trial
Source: J Med Internet Res. 2010 Dec 17;12(4):e71. doi: 10.2196/jmir.1338 (PMC3056526; doi:10.2196/jmir.1338)
Supplement: Supplementary file 3 [file jmir_v12i4e71_app3.pdf]

Consent to be a part of a research study at the University of Michigan

Title of study: *Automated Step-Count Feedback with Online Peer Support to Increase Physical Activity*

Principal Investigator: Dr. Caroline R. Richardson, M.D., Dept. of Family Medicine

Sponsor: Robert Wood Johnson Foundation

This research study looks at how to help people start and continue a walking program. It uses pedometers (a device worn at the waist that counts steps) to measure walking, and a website with goals and tailored messages to increase walking gradually. Because we think that support from fellow walkers may help, some of the participants will get to post messages to other participants online. This study will include about 300 participants, 240 in the group that can post messages to each other online, and 60 in the group that cannot. Group assignment will be random, using a computer process like the flip of a coin. Participants will be adults who are overweight and/or have heart disease and/or have type 2 diabetes who have access to a computer with Windows and an internet connection.

If you decide to be a part of the study, you would wear a pedometer every day for about 17 weeks (a little more than four months), and check the website and upload your pedometer over the internet as often as you want (but at least once a week). You would also take surveys online at the beginning and end, and a few times throughout the study. At the end of the study, you would get to keep your pedometer, and you would receive a $25 gift certificate and a yearlong membership to WalkingSpree.com. If you are a UM employee, you would be paid through payroll instead of by gift certificate. There is no financial cost to you or your insurance to participate in this study.

You do not have to participate. Participation in this study is completely voluntary, and will not affect your health care in any way. You are free to leave at any time with no penalty. If you choose not to participate, you may start your own exercise program. If you decide to leave the study before it is finished, please let the researchers know. In certain cases, the researchers might decide it is best for you not to finish the study. For example, if your health changes it may not be safe for you to continue the study. If you are participating in another study, please tell the researchers for both studies.

As with any research study, there may be some risks including minor muscle or joint injury, loss of confidentiality, or unpleasant interaction with other participants on the online message board. A walking program could make some health problems worse, such as heart problems, blood pressure problems (too high or too low), breathing problems (like asthma), problems related to your diabetes (low blood sugar, foot sores), or problems related to getting enough water when you exercise (if you are on a fluid restriction or diuretic). If you have any concerns or symptoms -- now or at any time during the study – please talk to your doctor and the study team.

You may benefit from increasing your walking by improving your fitness, and preventing or improving heart disease or diabetes. Your participation in this study might benefit others as we learn how to motivate people to exercise.

To make this study work, the researchers need to collect and store some information about you, such as your name and address, and information about your health. All the information we store, we take from you directly or with your permission; we take security precautions such as encryption on our study website, an internet “firewall,” and locked

physical storage. To compensate participants, we also need to collect social security numbers (UMIDs for employees at UM), which will be stored in a locked cabinet away from other study records.

Contact information

Principal Investigator:	Dr. Caroline Richardson	Study Coordinator:	Adrienne Janney
Telephone:	(800) 385-4128	Telephone:	(800) 385-4128

Mailing Address: 1018 Fuller Street
Ann Arbor, MI 48109-5708
Email address: studyteam@steppinguptohealth.org

You may also express a concern about a study by contacting the Institutional Review Board listed below, or by calling the University of Michigan Compliance Help Line at 1-888-296-2481.

University of Michigan Medical School Institutional Review Board (IRBMED)

Argus I	Telephone: 734-763-4768
517 W. William	Fax: 734-615-1622
Ann Arbor, MI 48103-4943	e-mail: irbmed@umich.edu

If you are concerned about a possible violation of your privacy, contact the University of Michigan Health System Privacy Officer at 1-888-296-2481. When you call or write about a concern, please provide as much information as possible, including the name of the researcher, the IRBMED number (at the top of this form), and details about the problem. This will help University officials to look into your concern. When reporting a concern, you do not have to give your name unless you want to.

I have read the above, and am satisfied with my understanding of this study, its possible benefits and risks, and my other choices. My questions so far have been answered. I understand that if I have more questions or concerns about the study or my participation as a research subject, I may contact one of the people listed in Section 10 (above). I understand that I am able to print a copy of this form, and that if I consent, I will also receive a copy of this form in the mail.

<Print a copy of this consent form.> If you understand, and agree to enroll in this research study, please choose “Yes, enroll me now,” below.

__ YES, enroll me now. I agree to participate in this research study.

__ NO, thank you.

__NOT YET; I am interested in this study, but I have some questions, and would like to talk to a member of the study staff.

Consent Subtitle: ________________________

Consent Version: ________________________
