# Supplementary material for: An Online Community Improves Adherence in an Internet-Mediated Walking Program. Part 1: Results of a Randomized Controlled Trial
Source: J Med Internet Res. 2010 Dec 17;12(4):e71. doi: 10.2196/jmir.1338 (PMC3056526; doi:10.2196/jmir.1338)
Supplement: Supplementary file 5 [file jmir_v12i4e71_app5.pdf]

Stepping Up to Health is providing you with both a pedometer and a website to help you walk more. To begin the program, please answer the following survey questions so we can learn more about you, your current level of activity, and other questions related to your lifestyle. The survey must be taken in one sitting, so you might want to set aside some time to take this survey. If you quit before the end of the survey, your answers will not be saved, and you will have to start over when you return. It takes most people 20-30 minutes to complete this survey. Please answer each question. Your honest answers will provide the keys we need to create a program that meets your individual needs. The more we know, the more we can develop tools that make sense to you. However, you are not required to answer any question. When you complete the main survey, you may be directed to up to three disease-specific sections. You will have a chance to take a break before filling out the additional sections.

{THEREAREXQUESTIONS}

Section 1

Question Code: 2X2X5

Short Desc: 1EnjoyRecAct

Do you participate in any of the following social or recreational activities?

Check any that apply

- ☐ Bowling (2X2X5Bowl)
- ☐ Gardening (2X2X5Garden)
- ☐ Golfing (2X2X5Golf)
- ☐ Shuffleboard (2X2X5Shuffle)
- ☐ Reading (2X2X5Read)
- ☐ Watching television (2X2X5TV)
- ☐ Bicycling (2X2X5Bike)
- ☐ Fishing (2X2X5Fish)
- ☐ Tennis (2X2X5Tennis)
- ☐ Swimming (2X2X5Swim)
- ☐ Bingo (2X2X5Bingo)
- ☐ Playing cards (2X2X5Cards)
- ☐ Attending church or social clubs (2X2X5Church)
- ☐ Going to lunch or coffee with friends (2X2X5Friends)
- ☐ I do not partake in any recreational activities (2X2X5None)

Question Code: 2X2X6

Short Desc: 2aSitAct

Over the past 7 days in your leisure time, how often did you participate in sitting activities such as

reading, watching TV or doing handcrafts?
Choose only one of the following

- Never (Never)
- Seldom (1-2 days) (Seldom)
- Sometimes (3-4 days) (Sometimes)
- Often (5-7 days) (Often)

Question Code: 2X2X7
Short Desc: 2bSitActWriteIn

What were these activities?
SEE QUSTIONCODE ABOVE

Question Code: 2X2X8
Short Desc: 2cHrsSitAct

On average, how many hours per day did you engage in these sitting activities in your leisure time?
Choose only one of the following

- Less than 1 hour (Less1hour)
- 1 but less than 2 hours (1to2hours)
- 2-4 hours (2to4hours)
- More than 4 hours (More4hours)

Question Code: 2X2X9
Short Desc: 3aWalkOutside

Over the past 7 days, how often did you take a walk outside your home or yard for any reason? For example, for fun or exercise, walking to work, walking the dog, etc.?
Choose only one of the following

- Never (Never)
- Seldom (1-2 days) (Seldom)
- Sometimes (3-4 days) (Sometimes)
- Often (5-7 days) (Often)

Question Code: 2X2X10
Short Desc: 3bWalkOutsideWriteIn

What were these activities?
SEE QUSTIONCODE ABOVE

Question Code: 2X2X11
Short Desc: 3cHrsWalkOutside

On average, how many hours (minutes) per day did you spend walking?
Choose only one of the following

- ○ Less than 1 hour (Less1hour)
- ○ 1 but less than 2 hours (1to2hours)
- ● 2-4 hours (2to4hours)
- ○ More than 4 hours (More4hours)

Question Code: 2X2X12
Short Desc: 4aLightRecAct

Over the past 7 days, how often did you engage in light sport or recreational activities such as bowling, golf with a cart, shuffleboard, fishing from a boat or pier or other similar activities?
Choose only one of the following

- ○ Never (Never)
- ○ Seldom (1-2 days) (Seldom)
- ● Sometimes (3-4 days) (Sometimes)
- ○ Often (5-7 days) (Often)

Question Code: 2X2X14
Short Desc: 4bLightRecActWriteIn

What were these activities?
SEE QUSTIONCODE ABOVE

Question Code: 2X2X15
Short Desc: 4cHrsLightRecAct

On average, how many hours per day did you engage in these light sport or recreational activities?
Choose only one of the following

- ○ Less than 1 hour (Less1hour)
- ○ 1 but less than 2 hours (1to2hours)
- ● 2-4 hours (2to4hours)
- ○ More than 4 hours (More4hours)

Question Code: 2X2X16
Short Desc: 5aModRecAct

Over the past 7 days, how often did you engage in moderate sport and recreational activities such as doubles tennis, ballroom dancing, hunting, ice skating, golf without a cart, softball or other similar activities?

Choose only one of the following

- Never (Never)
- Seldom (1-2 days) (Seldom)
- Sometimes (3-4 days) (Sometimes)
- Often (5-7 days) (Often)

Question Code: 2X2X17

Short Desc: 5bModRecActWriteIn

What were these activities?

SEE QUSTIONCODE ABOVE

Question Code: 2X2X18

Short Desc: 5cHrsModRecAct

On average, how many hours per day did you engage in these moderate sport or recreational activities?

Choose only one of the following

- Less than 1 hour (Less1hour)
- 1 but less than 2 hours (1to2hours)
- 2-4 hours (2to4hours)
- More than 4 hours (More4hours)

Question Code: 2X2X19

Short Desc: 6aStrenRecAct

Over the past 7 days, how often did you engage in strenuous sport and recreational activities such as jogging, swimming, cycling, singles tennis, aerobic dance, skiing (downhill or cross-country) or other similar activities?

Choose only one of the following

- Never (Never)
- Seldom (1-2 days) (Seldom)
- Sometimes (3-4 days) (Sometimes)
- Often (5-7 days) (Often)

Question Code: 2X2X20

Short Desc: 6bStrenRecActWriteIn

What were these activities?

SEE QUSTIONCODE ABOVE

Question Code: 2X2X21
Short Desc: 6cHrsStrenRecAct

On average, how many hours per day did you engage in these strenuous sport or recreational activities?
Choose only one of the following

- ○ Less than 1 hour (Less1hour)
- ○ 1 but less than 2 hours (1to2hours)
- ● 2-4 hours (2to4hours)
- ○ More than 4 hours (More4hours)

Question Code: 2X2X22
Short Desc: 7aEndurEx

Over the past 7 days, how often did you do any exercises specifically to increase muscle strength and endurance, such as lifting weights or pushups, etc.?
Choose only one of the following

- ○ Never (Never)
- ○ Seldom (1-2 days) (Seldom)
- ● Sometimes (3-4 days) (Sometimes)
- ○ Often (5-7 days) (Often)

Question Code: 2X2X23
Short Desc: 7bEndurExWriteIn

What were these activities?

SEE QUSTIONCODE ABOVE

Question Code: 2X2X24
Short Desc: 7cHrsEndurEx

On average, how many hours per day did you engage in exercises to increase muscle strength and endurance?
Choose only one of the following

- ○ Less than 1 hour (Less1hour)
- ● 1 but less than 2 hours (1to2hours)
- ○ 2-4 hours (2to4hours)

- More than 4 hours (More4hours)

Question Code: 2X2X27
Short Desc: 9aWorkPayVol

During the past 7 days, did you work, either for pay or as a volunteer?
Choose only one of the following

- Yes (Yes)
- No (No)

Question Code: 2X2X28
Short Desc: 9bHrsWorkPayVol
Question Code: 2X2X29
Short Desc: 9cPhysActForJob

Section 2

Question Code: 2X5X26
Short Desc: 8a-f

During the past 7 days, did you engage in any of the following activities?

	Yes	No
Have you done any light housework, such as dusting or washing dishes? 2X5X26LtHousework		
Have you done any heavy housework or chores, such as vacuuming, scrubbing floors, washing windows, or carrying wood? 2X5X26HeavyHousework		
Home repairs like painting, wallpapering, electrical work, etc.? 2X5X26HomeRepairs		
Lawn work or yard care, including snow or leaf removal, wood chopping, etc.? 2X5X26LawnWork		
Outdoor gardening? 2X5X26Gardening		
Caring for another person, such as children, grandchildren, dependent spouse, or another adult?		

2X5X26CareForOther

Question Code: 2X5X30
Short Desc: 10OAMotWalk

Overall, how MOTIVATED are you to walk each day? Use the scale below.

	1 - Not Motivated	2	3	4	5	6	7	8	9	10 - Extremely Motivated
Choose One 2X5X30Menu	◯	◯	◯	◯	◯	◯	◯	◯	◯	◯

Question Code: 2X5X31
Short Desc: 11Int

How much have you THOUGHT about your reasons for exercising? Use the scale below.
Choose only one of the following

- ◯ 1 No thought at all (1)
- ◯ 2 (2)
- ◯ 3 (3)
- ◯ 4 (4)
- ◯ 5 (5)
- ◯ 6 (6)
- ◯ 7 (7)
- ◯ 8 (8)
- ◯ 9 (9)
- ◯ 10 A great deal of thought (10)

Question Code: 2X5X32
Short Desc: 12Int

There are many REASONS people decide to walk. Please tell us how important each reason is for YOU.

	1 Not at all important	2	3	4	5	6	7 Very important
To prevent future health problems 2X5X32IMPreventFuture	◯	◯	◯	◯	◯	◯	◯
I feel guilty for not	◯	◯	◯	◯	◯	◯	◯

exercising more
2X5X32NMGuilt

Other people want me to
2X5X32EMOtherWant

To take responsibility for my own health
2X5X32IMResponsible

Exercise sets a good example for my family
2X5X32EMGdExFam

To improve my physical health
2X5X32IMPhysHealth

My doctor told me to exercise more
2X5X32EMDoctor

I enjoy exercise
2X5X32IMEnjoy

I want to set a good example for my community
2X5X32EMGdExCom

I believe it is a good thing
2X5X32IMBelieve

If I don't exercise, I know I will regret it
2X5X32NMRegret

Others would be upset with me if I didn't
2X5X32EMUpsetOthers

To manage my weight
2X5X32IMManageWeight

I don't want to let others down
2X5X32EMOthersDown

To improve my ability to do daily activities 2X5X32IMDailyAct	○	○	○	○	○	○	○
To increase my energy levels 2X5X32IMIncreaseEnerg	○	○	○	○	○	○	○
I want others to see that I can 2X5X32EMOthersSee	○	○	○	○	○	○	○
It is easier to do what I am told 2X5X32EMTold	○	○	○	○	○	○	○
I would feel bad about myself if I didn't 2X5X32NMBadAboutSelf	○	○	○	○	○	○	○
To control an existing health problem 2X5X32MotControlExist	○	○	○	○	○	○	○
It will give me a sense of accomplishment 2X5X32IMAccomplishmen	○	○	○	○	○	○	○
To feel less tension and stress 2X5X32MotLessTension	○	○	○	○	○	○	○
To feel more attractive 2X5X32MotAttractive	○	○	○	○	○	○	○

Question Code: 2X5X33
Short Desc: 13OAConWalk

Overall, how CONFIDENT are you that you can walk each day? Use the scale below.
Choose only one of the following

- ○ 1 Not at all Confident
- ○ 2
- ○ 3
- ○ 4
- ○ 5
- ○ 6
- ○ 7

- 8
- 9
- 10 Extremely Confident

Question Code: 2X5X34
Short Desc: 14OAC

Listed below are issues that can make it DIFFICULT for people to exercise. How confident are you that you can exercise when:

	1 Not at all Confident	2	3	4	5	6	7 Very Confident
You feel you don't have the time to exercise 2X5X34BarTime							
The weather is hot 2X5X34BarHotWeath							
The weather is cold 2X5X34BarColdWeath							
Health issues make it difficult 2X5X34BarHealthProb							
You're afraid of getting hurt 2X5X34BarHurt							
It takes too much effort 2X5X34BarEffort							
It's too expensive to join a club or gym 2X5X34BarExpensive							
You don't feel you have a safe place to exercise 2X5X34BarSafePlace							
You might sweat 2X5X34BarSweat							
You don't have anyone to exercise with							

2X5X34BarNoOne

You feel self conscious about your looks during exercise 2X5X34BarSelfConsciou	○	○	○	○	○	○	○
You don't have energy 2X5X34BarEnergy	○	○	○	○	○	○	○
You feel tired 2X5X34BarTired	○	○	○	○	○	○	○
You're in a bad mood 2X5X34BarBadMood	○	○	○	○	○	○	○

Question Code: 2X5X35
Short Desc: 15Cur

Do you currently GET support from your family or friends in getting enough physical activity?
Choose only one of the following

- ○ Yes (Yes)
- ○ No (No)

Question Code: 2X5X36
Short Desc: 16WantSupport

Do you WANT support from your family or friends in getting enough physical activity?
Choose only one of the following

- ○ Yes (Yes)
- ○ No (No)

Question Code: 2X5X37
Short Desc: 17WhoSupport

Section 3

Question Code: 2X6X38
Short Desc: 18Nei

Please choose the answer that best applies to you and your neighborhood. Both "local" and "within walking distance" mean a 20-minute walk from your home or less.

	Strongly Disagree			Strongly Agree
I can do most of my shopping at local stores. 2X6X38LocalStores				
Stores are within easy walking distance of my home. 2X6X38StoresWalkDist				
Parking is difficult in local shopping areas. 2X6X38ParkingDiff				
There are many places to go within easy walking distance of my home. 2X6X38ManyPlacesWalk				
It is easy to walk to a bus/subway/train stop from my home. 2X6X38EasyWalkTransit				
The streets in my neighborhood are hilly, making my neighborhood difficult to walk in. 2X6X38StreetsHillyDif				

Question Code: 2X6X39
Short Desc: 19Saf

Please choose the answer that best applies to you and your neighborhood. Both "local" and "within walking distance" mean a 20-minute walk from your home or less.

	Strongly Disagree			Strongly Agree
There are sidewalks or walking paths that I feel are safe enough to walk on in my neighborhood. 2X6X39SidewalksSafe				
I feel safe walking in my neighborhood during the day. 2X6X39SafeWalkDay				

I feel safe walking in my neighborhood at night. 2X6X39SafeWalkNight

Question Code: 2X6X40
Short Desc: 20OwnDog

Do you own a dog?
Choose only one of the following

- Yes (Yes)
- No (No)

Question Code: 2X6X41
Short Desc: 21WalkDog
Question Code: 2X6X42
Short Desc: 22OftenWalkDog

Section 4

Question Code: 2X7X43
Short Desc: 23GenHealth

In general, how would you rate your health?
Choose only one of the following

- Excellent (Excellent)
- Very good (VeryGood)
- Good (Good)
- Fair (Fair)
- Poor (Poor)

Question Code: 2X7X44
Short Desc: 24GenHealth

Please rate how much your health currently limits you in the following activities.

	Not at all Limited	Somewhat Limited	Significantly Limited
Moderate intensity activities, such as racquet sports, pushing a vacuum cleaner, bowling, golf, bicycling, swimming, or fast walking 2X7X44SF36d			

Low intensity activities, such as casual walking, home maintenance, or gardening 2X7X44SF36L	○	○	○
Climbing several flights of stairs 2X7X44SF36S	○	○	○
Climbing one flight of stairs 2X7X44SF36O	○	○	○

Question Code: 2X7X45
Short Desc: 25GenHealth

For each question please give the one answer that comes closest to the way you have been feeling in the last 4 weeks. How much of the time:

	None of the time	A little of the time	A good bit of the time	Most of the time	All of the time
Have you felt calm and peaceful? 2X7X45CalmPeaceful	○	○	○	○	○
Did you have a lot of energy? 2X7X45HaveEnergy	○	○	○	○	○
Have you felt downhearted and blue? 2X7X45DownheartedBlue	○	○	○	○	○

Question Code: 2X7X46
Short Desc: 26HealthHistory

Do you or have you ever suffered from, or have you been diagnosed with, any of the following?
Check any that apply

- ☐ Stroke (2X7X46Strok)
- ☐ Parkinson's disease (2X7X46Parki)
- ☐ Lung disease, emphysema, asthma or bronchitis (2X7X46Asthm)
- ☐ Arthritis (2X7X46Arthr)
- ☐ Osteoporosis or thin bones (2X7X46Osteo)
- ☐ Depression, anxiety or an emotional problem

(2X7X46MdDis)
- Neurological disorder (2X7X46NeurD)
- Sleep problems such as insomnia or narcolepsy (2X7X46Sleep)
- Chronic pain (2X7X46Chron)
- A hip or knee joint replacement surgery (2X7X46Joint)
- Liver problems (2X7X46Liver)
- Type 2 diabetes (2X7X46Diabe)
- High blood pressure (2X7X46HBP)
- Impaired glucose tolerance (pre-diabetes) (2X7X46IGT)
- High cholesterol (2X7X46HiCho)
- Breast cancer (2X7X46Breas)
- Colon cancer (2X7X46Colon)
- Prostate cancer (2X7X46Prost)
- Lung cancer (2X7X46LungC)
- Other cancer (2X7X46Other)
- Stomach or digestive disorder (2X7X46Diges)
- Kidney problems (2X7X46Kidne)
- Angina or chest pain from heart disease (2X7X46Angina)
- Heart failure (2X7X46HeartFail)
- Heart attack (2X7X46HeartAttack)
- Coronary artery disease (2X7X46CAD)

Question Code: 2X7X47
Short Desc: 27HealthHistory

Each item below is a belief statement about your health with which you may agree or disagree. Please choose a response along the scale that best represents how you feel.

	**1 - Strongly disagree**	**2**	**3**	**4**	**5**		**7 - Strongly agree**
The main thing that affects my health is what I myself do 2X7X47MHLCWhatIDoAffe	○	○	○	○	○	○	○
If I take care of myself, I can avoid illness 2X7X47MHLCSelfCareAvo	○	○	○	○	○	○	○
If I take the right actions, I can stay healthy 2X7X47MHLCRightActSta	○	○	○	○	○	○	○

Question Code: 2X7X48
Short Desc: 28OtherBeh

Please tell us about other behaviors related to your health.
Check any that apply

- ☐ I wear my seat belt when driving or a passenger in a car. (2X7X48SeatBelt)
- ☐ I wear a helmet when riding a bicycle. (2X7X48BicHelmet)
- ☐ I wear a helmet when riding a motorcycle. (2X7X48MotorHelmet)
- ☐ I have a physical with a health care provider every 1-3 years. (2X7X48Physical)
- ☐ I practice a stress management technique (such as yoga or deep breathing). (2X7X48StressManage)
- ☐ I floss my teeth. (2X7X48Floss)
- ☐ I get a flu shot every year. (2X7X48FluShot)
- ☐ I take a multivitamin. (2X7X48MultiVit)
- ☐ I'm up to date with my shots (tetanus, diphtheria, measles, chicken pox, etc). (2X7X48Vacc)

Question Code: 2X7X49
Short Desc: 29TryingLoseWt

Are you currently trying to lose weight?
Choose only one of the following

- ○ Yes (Yes)
- ○ No (No)

Question Code: 2X7X50
Short Desc: 30SmokeCigs

Do you currently smoke cigarettes?
Choose only one of the following

- ○ Yes (Yes)
- ○ No, but I am a former smoker (NoFormer)
- ○ No, I have never smoked (NoNever)

Question Code: 2X7X51
Short Desc: 31CigsDaily
Question Code: 2X7X52
Short Desc: 32YrsAgoQuit
Question Code: 2X7X53
Short Desc: 33FamilyHealthHist

Does anyone in your immediate family (mother, father, sister, brother) have any of the following

conditions?
Check any that apply

- ☐ Arthritis (2X7X53Arthritis)
- ☐ Diabetes (2X7X53Diabetes)
- ☐ Impaired glucose tolerance (pre-diabetes) (2X7X53IGT)
- ☐ Cardiovascular disease (2X7X53CVD)
- ☐ Stroke (2X7X53Stroke)
- ☐ High cholesterol (2X7X53HiChol)
- ☐ High blood pressure (2X7X53HBP)
- ☐ Breast cancer (2X7X53BreastCancer)
- ☐ Colon cancer (2X7X53ColonCancer)
- ☐ Prostate cancer (2X7X53ProstateCancer)
- ☐ Other cancer (2X7X53OtherCancer)
- ☐ Osteoporosis (2X7X53Osteo)
- ☐ Overweight and/or obese (2X7X53OvrWtObese)
- ☐ I do not know my family health history (2X7X53DK)

Section 5

Question Code: 2X10X82
Short Desc: 34PedUse

Have you ever used a pedometer?
Choose only one of the following

- ○ Yes (Yes)
- ○ No (No)

Question Code: 2X10X83
Short Desc: 35Ped30Days
Question Code: 2X10X87
Short Desc: 39MaritalStatus

What is your current marital status?
Choose only one of the following

- ○ Single (Single)
- ○ Married (Married)
- ○ Living with partner (Partner)
- ○ Separated (Separated)
- ○ Divorced (Divorced)
- ○ Widowed (Widowed)
- ○ Other (Other)

Question Code: 2X10X88
Short Desc: 40HispanicLatino

Do you consider yourself to be Hispanic or Latino (a

person of Mexican, Puerto Rican, Cuban, South or Central American, or other Spanish culture or origin)?

Choose only one of the following

- Yes, Hispanic or Latino (Yes)
- No, not Hispanic or Latino (No)

Question Code: 2X10X89
Short Desc: 41Race

What race do you consider yourself to be?

Check any that apply

- American Indian or Alaska Native. A person having origins in any of the original peoples of North, Central, or South America, and who maintains tribal affiliation or community attachment. (2X10X89AmIndian)
- Asian. A person having origins in any of the original peoples of the Far East, Southeast Asia, or the Indian subcontinent. (2X10X89Asian)
- Black or African-American. A person having origins in any of the black racial groups of Africa. (2X10X89Black)
- Native Hawaiian or Other Pacific Islander. A person having origins in any of the original peoples of Hawaii, Guam, Samoa, or other Pacific Islands. (2X10X89PacIsland)
- White. A person having origins in any of the original peoples of Europe, the Middle East, or North Africa. (2X10X89White)
- Other race (2X10X89OtherRace)

Other:

Question Code: 2X10X90
Short Desc: 42HomeStatus

Do you live alone?

Choose only one of the following

- Yes (Yes)
- No (No)

Question Code: 2X10X91
Short Desc: 43AdultInHome

What other adults live with you?

Check any that apply

- Spouse (2X10X91Spouse)
- Partner (2X10X91Partner)
- Friend (2X10X91Friend)
- Adult children (2X10X91AdultChild)

- Immediate family (mother, father, brother, sister) (2X10X91ImFamily)
- Relative (2X10X91Relative)
- Other adults (2X10X91OthAdult)

Question Code: 2X10X92
Short Desc: 44ChildInHome

Do any children under the age of 18 live in your home?
Choose only one of the following

- Yes (Yes)
- No (No)

Question Code: 2X10X93
Short Desc: 45EducationLevel

What is the highest grade or level of schooling you have completed?
Choose only one of the following

- 7th grade or less (Lessthan7)
- Between 8th and 11th grade (8to11)
- 12th grade or completed high school or GED (12orGED)
- Post high school training other than college (e.g., vocational or technical) (PostHiSchool)
- Some college (SomeCollege)
- 2-year college graduate (Associate's degree) (2YearCollege)
- College graduate (CollegeGrad)
- Postgraduate (Postgraduate)

Question Code: 2X10X94
Short Desc: 46Employment

Which of the following best describes your current employment status?
Check any that apply

- Working full time, 35 hours or more a week (2X10X94FullTime)
- Working part time, less than 35 hours a week (2X10X94PartTime)
- Unemployed or laid off and looking for work (2X10X94UnLooking)
- Unemployed and not looking for work (2X10X94UnNotLooking)
- Homemaker (2X10X94Homemaker)

- In school (2X10X94Student)
- Retired (2X10X94Retired)
- Disabled, not able to work (2X10X94Disabled)
- Other (2X10X94EmployOther)

Question Code: 2X10X95
Short Desc: 47Income

Which of the categories best describes your total annual combined household income from all sources?
Choose only one of the following

- Less than $5,000 (Less5K)
- $5,000 to $9,999 (5Kto9999)
- $10,000 to $14,999 (10Kto14999)
- $15,000 to $19,999 (15Kto19999)
- $20,000 to $29,999 (20Kto29999)
- $30,000 to $39,999 (30Kto39999)
- $40,000 to $49,999 (40Kto49999)
- $50,000 to $59,999 (50Kto59999)
- $60,000 to $69,999 (60Kto69999)
- $70,000 and over (70KMore)

Question Code: 2X10X96
Short Desc: 48InternetAbil

How would you rate your ability to use the Internet?
Choose only one of the following

- Limited (Limited)
- Basic (Basic)
- Moderate (Mod)
- Advanced (Adv)
- Expert (Expert)

Question Code: 2X10X97
Short Desc: 49InternetUse

Would you say that you use the Internet...
Choose only one of the following

- Every day (EveryDay)
- A few times a week (FewWk)
- Once a week (OnceWk)
- A few times a month (FewMnth)
- Once a month (OnceMnth)
- Less than once a month (LessOnceMnth)

next >>

[Exit and Clear Survey]
