# Supplementary material for: An Online Community Improves Adherence in an Internet-Mediated Walking Program. Part 1: Results of a Randomized Controlled Trial
Source: J Med Internet Res. 2010 Dec 17;12(4):e71. doi: 10.2196/jmir.1338 (PMC3056526; doi:10.2196/jmir.1338)
Supplement: Supplementary file 6 [file jmir_v12i4e71_app6.pdf]

Congratulations on completing two weeks of the Stepping Up to Health program! We would like to ask you some questions that will help us to know how and why this program works or does not work for you. This survey must be taken in one sitting, so you might want to set aside some time to take this survey. If you quit before the end of the survey, your answers will not be saved, and you will have to start over when you return. It takes most people 10-15 minutes to complete this survey. Please answer each question. Your honest answers will provide the keys we need to create a program that meets your individual needs. The more we know, the more we can develop tools that make sense to you. You are not required to answer any question.

Section 1

Question Code: 9X23X164

Short Desc: 1effect

How effective do you think the following would be in helping to improve your health?

	1 -Not effective	2	3	4	5 -Very effective	Not applicable
Taking additional medications 9X23X164meds	1	2	3	4	5	na
Improving diet 9X23X164diet	1	2	3	4	5	na
Exercising more 9X23X164exerc	1	2	3	4	5	na
Bariatric surgery 9X23X164bariac	1	2	3	4	5	na

Section 2

Question Code: 9X24X165

Short Desc: 1fraction

	1-not good at all	2	3	4	5	6-Extremely good
How good are you at working with fractions? 9X24X165frac	1	2	3	4	5	6

How good are you at working with percentages? 9X24X165precent	1	2	3	4	5	6
How good are you at calculating a 15% tip? 9X24X165tip	1	2	3	4	5	6
How good are you at figuring out how much a shirt will cost if it is 25% off? 9X24X165shirtsale	1	2	3	4	5	6
When reading the newspaper, how helpful do you find tables and graphs that are parts of a story? 9X24X165tables	1	2	3	4	5	6

Question Code: 9X24X166
Short Desc: 2wordsnums

	**Always prefer words**	**2**	**3**	**4**	**5**	**Always prefer numbers**
When people tell you the chance of something happening, do you prefer that they use words ("it rarely happens") or numbers ("there's a 1% chance")? 9X24X166wordnum	1	2	3	4	5	6

Question Code: 9X24X167
Short Desc: 3percpred

	**Always Prefer Percentages**	**2**	**3**	**4**	**5**	**Always Prefer Words**
When you hear a weather forecast, do you prefer predictions using percentages (e.g., "there will be a 20% chance of rain today") or predictions using only words (e.g., "there is a	1	2	3	4	5	6

small chance of rain today")?
9X24X167percentwords

Question Code: 9X24X168
Short Desc: 4numuse

	Never	2	3	4	5	Very Often
How often do you find numerical information to be useful? 9X24X168numbuse	1	2	3	4	5	6

Section 3

Question Code: 9X25X169
Short Desc: respond

Please respond to the following statements:

	1-strongly disagree	2	3	4-strongly agree
I enjoy seeing someone else do something that neither of us is supposed to do. 9X25X169somelse	1	2	3	4
I have a strong desire to maintain my personal freedom. 9X25X169freedom	1	2	3	4
I enjoy playing "devil's advocate" whenever I can. 9X25X169devadv	1	2	3	4
In discussions, I am easily persuaded by others. 9X25X169persuade	1	2	3	4

It would be better to have more freedom to do what I want on a job. 9X25X169jobfree	1	2	3	4
If I am told what to do, I often do the opposite. 9X25X169opp	1	2	3	4
I am sometimes afraid to disagree with others. 9X25X169disothers	1	2	3	4
It does not upset me to change my plans because someone in the group wants to do something else. 9X25X169plans	1	2	3	4
I am very open to solutions to my problems from others. 9X25X169solution	1	2	3	4
I enjoy "showing up" people who think they are right. 9X25X169showup	1	2	3	4
I usually go along with others' advice. 9X25X169otheradv	1	2	3	4
I feel it is better to stand up for what I believe than to be silent. 9X25X169standup	1	2	3	4
I am very stubborn and set in my ways. 9X25X169stubborn	1	2	3	4

Section 4

Question Code: 9X26X170

Short Desc: attbel

Read each of the following statements and decide how much you agree with each according to your attitudes, beliefs, and experiences. It is important for you to realize that there are no "right" or "wrong" answers to these questions. People are different, and we are interested in how you feel. Please respond according to the following 6-point scale:

	Strongly Disagree	Moderately Disagree	Slightly Disagree	Slightly Agree	Moderately Agree	Strongly Agree
It upsets me to go into a situation without knowing what I can expect from it. 9X26X170sitknow	1	2	3	4	5	6
I'm not bothered by things that interrupt my daily routine. 9X26X170dailrout	1	2	3	4	5	6
I enjoy having a clear and structured mode of life. 9X26X170lifemode	1	2	3	4	5	6
I like to have a place for everything and everything in its place. 9X26X170place	1	2	3	4	5	6
I find that a well-ordered life with regular hours makes my life tedious. 9X26X170wellorder	1	2	3	4	5	6
I don't like situations that are uncertain. 9X26X170uncert	1	2	3	4	5	6
I hate to change my plans at the last minute. 9X26X170planchange	1	2	3	4	5	6
I hate to be with people who are unpredictable. 9X26X170unpredpeople	1	2	3	4	5	6

I find that a consistent routine enables me to enjoy life more. 9X26X170consist	1	2	3	4	5	6
I enjoy the exhilaration of being in unpredictable situations. 9X26X170unpredsit	1	2	3	4	5	6
I become uncomfortable when the rules in a situation are not clear. 9X26X170uncomfrules	1	2	3	4	5	6
If I become sick, I have the power to make myself well again. 9X26X170sickwell	1	2	3	4	5	6
Often I feel that no matter what I do, if I am going to get sick, I will get sick. 9X26X170sicksick	1	2	3	4	5	6
If I see an excellent doctor regularly, I am less likely to have health problems. 9X26X170doctor	1	2	3	4	5	6
It seems that my health is greatly influenced by accidental happenings. 9X26X170acchealth	1	2	3	4	5	6
I can only maintain my health by consulting health professionals. 9X26X170healthprof	1	2	3	4	5	6
I am directly responsible for my health. 9X26X170dirresp	1	2	3	4	5	6
Other people play a big	1	2	3	4	5	6

part in whether I stay healthy or become sick. 9X26X170othpeop						
Whatever goes wrong with my health is my own fault. 9X26X170healthown	1	2	3	4	5	6
When I am sick, I just have to let nature run its course. 9X26X170sicknat	1	2	3	4	5	6
Health professionals keep me healthy. 9X26X170healthyprof	1	2	3	4	5	6
When I stay healthy, I'm just plain lucky. 9X26X170healthluck	1	2	3	4	5	6
My physical well-being depends on how well I take care of myself. 9X26X170physwell	1	2	3	4	5	6
When I feel ill, I know it is because I have not been taking care of myself properly. 9X26X170selfcare	1	2	3	4	5	6
The type of care I receive from other people is what is responsible for how well I recover from an illness. 9X26X170othercare	1	2	3	4	5	6
	Strongly Disagree	Moderately Disagree	Slightly Disagree	Slightly Agree	Moderately Agree	Strongly Agree
Even when I take care of myself, it's easy to get sick. 9X26X170easysick	1	2	3	4	5	6
When I become ill, it's a	1	2	3	4	5	6

matter of fate.<br>9X26X170illfate						
I can pretty much stay healthy by taking good care of myself.<br>9X26X170healthself	1	2	3	4	5	6
Following doctor's orders to the letter is the best way for me to stay healthy.<br>9X26X170docorders	1	2	3	4	5	6

submit

[Exit and Clear Survey]
