# Supplementary material for: An Online Community Improves Adherence in an Internet-Mediated Walking Program. Part 1: Results of a Randomized Controlled Trial
Source: J Med Internet Res. 2010 Dec 17;12(4):e71. doi: 10.2196/jmir.1338 (PMC3056526; doi:10.2196/jmir.1338)
Supplement: Supplementary file 7 [file jmir_v12i4e71_app7.pdf]

To be administered one week prior to end of study. Questions 5-7 go to participants in the forums intervention arm only.

Congratulations! You're almost done with *Stepping Up to Health*. As you begin your final week of our research program, we have a handful of short questions for you. You should be able to answer them in about 5 minutes.

1) What is your current weight in pounds? (fill in)

2) Prior to starting the Stepping Up to Health program, how often did you **READ** messages or posts on

a) message boards or online forums? Never Rarely Monthly Weekly Daily Don't Know

b) email groups or listservs? Never Rarely Monthly Weekly Daily Don't Know

c) instant messenger or online chat rooms? Never Rarely Monthly Weekly Daily Don't Know

d) blogs? Never Rarely Monthly Weekly Daily Don't Know

e) text messages on a cell phone? Never Rarely Monthly Weekly Daily Don't Know

3) Prior to starting the Stepping Up to Health program, how often did you **POST or SEND** messages or comments on

a) message boards or online forums? Never Rarely Monthly Weekly Daily Don't Know

b) email groups or listservs? Never Rarely Monthly Weekly Daily Don't Know

c) instant messenger or online chat rooms? Never Rarely Monthly Weekly Daily Don't Know

d) blogs? Never Rarely Monthly Weekly Daily Don't Know

e) text messages on a cell phone? Never Rarely Monthly Weekly Daily Don't Know

4) Did you peek or remove the sticker from your pedometer before you were supposed to? (Think back to when you first received your pedometer with a sticker hiding the numbers. Did you look under the sticker before you received your first goal email or before you were able to see your graphs?)

To be administered one week prior to end of study. Questions 5-7 go to participants in the forums intervention arm only.

Yes, I peeked.

No, I did not peek.

I'm not sure.

5) Did the ability to talk to or read posts from other participants motivate you to walk more?

Yes

No

Don't Know

6) What type of discussions in the forums did you find most helpful?

(Leave as an open-ended question)

7) Did any of the following concerns make you hesitant to post messages on the forums? Check all that apply.

I was concerned about my privacy.

I did not have time.

I couldn't figure out how to post.

I don't think I am good at writing.

I had nothing to add.

It didn't interest me.

I didn't know about the forums.

Other
