# Supplementary material for: An Online Community Improves Adherence in an Internet-Mediated Walking Program. Part 1: Results of a Randomized Controlled Trial
Source: J Med Internet Res. 2010 Dec 17;12(4):e71. doi: 10.2196/jmir.1338 (PMC3056526; doi:10.2196/jmir.1338)
Supplement: Supplementary file 8 [file jmir_v12i4e71_app8.pdf]

Congratulations on completing to the Stepping Up to Health program! We would like to ask you some questions that will help us to know how and why this program works or does not work for you. Some of the questions here we asked you at the beginning of the program, but we would like to know if your answers have changed over time.

This survey must be taken in one sitting, so you might want to set aside some time to take this survey. If you quit before the end of the survey, your answers will not be saved, and you will have to start over when you return. It takes most people 20-30 minutes to complete this survey.

Please answer each question. Your honest answers will provide the keys we need to create a program that meets your individual needs. The more we know, the more we can develop tools that make sense to you. You are not required to answer any question.

{THEREAREXQUESTIONS}

Section 1

Question Code: 8X16X105

Short Desc: 1part

Do you participate in any of the following social or recreational activities? Check any that apply.

Check any that apply

- ☐ Bowling (8X16X105Bowling)
- ☐ Gardening (8X16X105Gardening)
- ☐ Golfing (8X16X105Golf)
- ☐ Shuffleboard (8X16X105Shuf)
- ☐ Reading (8X16X105read)
- ☐ Watching television (8X16X105TV)
- ☐ Bicycling (8X16X105Bicycling)
- ☐ Fishing (8X16X105Fishing)
- ☐ Tennis (8X16X105Tennis)
- ☐ Swimming (8X16X105Swimming)
- ☐ Bingo (8X16X105Bingo)
- ☐ Playing cards (8X16X105cards)
- ☐ Attending church or social clubs (8X16X105church)
- ☐ Going to lunch or coffee with friends (8X16X105friends)
- ☐ I do not partake in any recreational activities (8X16X105rec)

Question Code: 8X16X106

Short Desc: 2aleisure

Over the past 7 days, in your leisure time, how often did you participate in sitting activities such as reading, watching TV or doing handcrafts?

Choose only one of the following

- Never (never)
- Seldom (1-2 days) (Seldom)
- Sometimes (3-4 days) (Sometimes)
- Often (5-7 days) (Often)

Question Code: 8X16X107
Short Desc: 2bact

What were these activities?

SEE QUSTIONCODE ABOVE

Question Code: 8X16X108
Short Desc: 2chours

On average, how many hours per day did you engage in these sitting activities in your leisure time?

Choose only one of the following

- Less than 1 hour (Less)
- 1 but less than 2 hours (1)
- 2-4 hours (2-4)
- More than 4 hours (more4)

Question Code: 8X16X109
Short Desc: 3awalk

Over the past 7 days, how often did you take a walk outside your home or yard for any reason? For example, for fun or exercise, walking to work, walking the dog, etc.?

Choose only one of the following

- Never (Never)
- Seldom (1-2 days) (sel)
- Sometimes (3-4 days) (Sometimes)
- Often (5-7 days) (Often)

Question Code: 8X16X110
Short Desc: 3bactivities

What were these activities?

SEE QUSTIONCODE ABOVE

Question Code: 8X16X111
Short Desc: 3chours

On average, how many hours (minutes) per day did you spend walking?
Choose only one of the following

- Less than 1 hour (less1)
- 1 but less than 2 hours (1)
- 2-4 hours (2-4)
- More than 4 hours (More4)

Question Code: 8X16X112
Short Desc: 4asport

Over the past 7 days, how often did you engage in light sport or recreational activities such as bowling, golf with a cart, shuffleboard, fishing from a boat or pier or other similar activities?
Choose only one of the following

- Never (Never)
- Seldom (1-2 days) (Seldom)
- Sometimes (3-4 days) (Sometimes)
- Often (5-7 days) (Often)

Question Code: 8X16X113
Short Desc: 4bact

What were these activities?
SEE QUSTIONCODE ABOVE

Question Code: 8X16X114
Short Desc: 4chours

On average, how many hours per day did you engage in these light sport or recreational activities?
Choose only one of the following

- Less than 1 hour (less1)
- 1 but less than 2 hours (1)
- 2-4 hours (2-4)
- More than 4 hours (more4)

Question Code: 8X16X115
Short Desc: 5amodsport

Over the past 7 days, how often did you engage in moderate sport and recreational activities such as doubles tennis, ballroom dancing, hunting, ice skating, golf without a cart, softball or similar activities?

Choose only one of the following

- Never (never)
- Seldom (1-2 days) (Seldom)
- Sometimes (3-4 days) (Sometimes)
- Often (5-7 days) (Often)

Question Code: 8X16X116
Short Desc: 5bact

What were these activities?

SEE QUSTIONCODE ABOVE

Question Code: 8X16X117
Short Desc: 5chours

On average, how many hours per day did you engage in these moderate sport or recreational activities?

Choose only one of the following

- Less than 1 hour (less1)
- 1 but less than 2 hours (1)
- 2-4 hours (2-4)
- More than 4 hours (Mor4)

Question Code: 8X16X118
Short Desc: 6astrenuous

Over the past 7 days, how often did you engage in strenuous sport and recreational activities such as jogging, swimming, cycling, singles tennis, aerobic dance, skiing (downhill or cross-country) or other similar activities?

Choose only one of the following

- Never (nev)
- Seldom (1-2 days) (Seldom)
- Sometimes (3-4 days) (Some)
- Often (5-7 days) (Often)

Question Code: 8X16X149
Short Desc: 6bact

What were these activities?

SEE QUSTIONCODE ABOVE

Question Code: 8X16X150
Short Desc: 6chours

On average, how many hours per day did you engage in those strenuous sport and recreational activities?

Choose only one of the following

- Less than 1 hour (a)
- 1 but less than 2 hours (b)
- 2-4 hours (c)
- More than 4 hours (d)

Question Code: 8X16X119
Short Desc: 7amuscle

Over the past 7 days, how often did you do any exercises specifically to increase muscle strength and endurance, such as lifting weights or pushups, etc.?

Choose only one of the following

- Never (nev)
- Seldom (1-2 days) (Seld)
- Sometimes (3-4 days) (Some)
- Often (5-7 days) (Often)

Question Code: 8X16X151
Short Desc: 7bact

What were these activities?

SEE QUSTIONCODE ABOVE

Question Code: 8X16X152
Short Desc: 7chours

On average, how many hours per day did you engage in exercises to increase muscle strength and endurance?

Choose only one of the following

- Less than 1 hour (a)
- 1 but less than 2 hours (b)
- 2-4 hours (c)

More than 4 hours (d)

Section 2

Question Code: 8X17X120
Short Desc: 8past7days

During the past 7 days, did you engage in any of the following activities?

	Yes	No
a) Light housework, such as dusting or washing dishes? 8X17X120a	Yes	No
b) Heavy housework or chores, such as vacuuming, scrubbing floors, washing windows, or carrying wood? 8X17X120b	Yes	No
c) Home repairs like painting, wallpapering, electrical work, etc.? 8X17X120c	Yes	No
d) Lawn work or yard care, including snow or leaf removal, wood chopping, etc.? 8X17X120d	Yes	No
e) Outdoor gardening? 8X17X120e	Yes	No
f) Caring for another person, such as children, grandchildren, dependent spouse, or another adult? 8X17X120f	Yes	No

Question Code: 8X17X121
Short Desc: 9motivated

Overall, how MOTIVATED are you to walk each day? Use the scale below.
Choose only one of the following

- 1 - Not Motivated
- 2
- 3
- 4
- 5
- 6
- 7
- 8
- 9

10 - Extremely Motivated

Question Code: 8X17X122
Short Desc: 10thought

How much have you THOUGHT about your reasons for exercising? Use the scale below.
Choose only one of the following

- 1 - No thought at all
- 2
- 3
- 4
- 5
- 6
- 7
- 8
- 9
- 10 - A great deal of thought

Question Code: 8X17X123
Short Desc: 11reasons

There are many REASONS people decide to walk. Please tell us how important each reason is for YOU.

	**1 - Not at all important**	**2**	**3**	**4**	**5**	**6**	**7 - Very Important**
To prevent future health problems 8X17X123a	1	2	3	4	5	6	7
I feel guilty for not exercising more 8X17X123b	1	2	3	4	5	6	7
Other people want me to 8X17X123c	1	2	3	4	5	6	7
To take responsibility for my own health 8X17X123d	1	2	3	4	5	6	7

Exercise sets a good example for my family 8X17X123e	1	2 3 4 5 6				7
To improve my physical health 8X17X123f	1	2 3 4 5 6				7
My doctor told me to exercise more 8X17X123g	1	2 3 4 5 6				7
I enjoy exercise 8X17X123h	1	2 3 4 5 6				7
I want to set a good example for my community 8X17X123i	1	2 3 4 5 6				7
I believe it is a good thing 8X17X123j	1	2 3 4 5 6				7
If I don't exercise, I know I will regret it 8X17X123k	1	2 3 4 5 6				7
Others would be upset with me if I didn't 8X17X123l	1	2 3 4 5 6				7
To manage my own weight 8X17X123m	1	2 3 4 5 6				7
I don't want to let others down 8X17X123n	1	2 3 4 5 6				7
To improve my ability to do daily activities 8X17X123o	1	2 3 4 5 6				7

To increase my energy levels 8X17X123p	1	2	3	4	5	6	7
I want others to see that I can 8X17X123q	1	2	3	4	5	6	7
It is easier to do what I am told 8X17X123r	1	2	3	4	5	6	7
I would feel bad about myself if I didn't 8X17X123s	1	2	3	4	5	6	7
To control an existing health problem 8X17X123t	1	2	3	4	5	6	7
It will give me a sense of accomplishment 8X17X123u	1	2	3	4	5	6	7
To feel tension and stress 8X17X123v	1	2	3	4	5	6	7
To feel more attractive 8X17X123w	1	2	3	4	5	6	7

Question Code: 8X17X124
Short Desc: 12confident

Overall, how CONFIDENT are you that you can walk each day? Use the scale below.
Choose only one of the following

- 1 Not at all Confident
- 2
- 3
- 4
- 5
- 6

- 7
- 8
- 9
- 10 Extremely Confident

Question Code: 8X17X125
Short Desc: 13difficult

Listed below are issues that some people feel make it DIFFICULT to exercise. Thinking of a range of numbers between one and seven, with one being "not at all a problem" and seven being "a huge problem," please tell us how much each of these issues is a problem for you.

	**1 - Not at all a problem**	**2**	**3**	**4**	**5**	**6**	**7 - A huge problem**
You feel you don't have the time to exercise 8X17X125a	1	2	3	4	5	6	7
The weather is hot 8X17X125b	1	2	3	4	5	6	7
The weather is cold 8X17X125c	1	2	3	4	5	6	7
Health issues make it difficult 8X17X125d	1	2	3	4	5	6	7
You're afraid of getting hurt 8X17X125e	1	2	3	4	5	6	7
It takes too much effort 8X17X125f	1	2	3	4	5	6	7
It's too expensive to join a club or gym 8X17X125g	1	2	3	4	5	6	7
You don't feel you have a safe place to exercise 8X17X125h	1	2	3	4	5	6	7
You might sweat 8X17X125i	1	2	3	4	5	6	7

You don't have anyone to exercise with 8X17X125j	1	2	3	4	5	6	7
You feel self-conscious about your looks during exercise 8X17X125k	1	2	3	4	5	6	7
You don't have energy 8X17X125l	1	2	3	4	5	6	7
You feel tired 8X17X125m	1	2	3	4	5	6	7
You're in a bad mood 8X17X125n	1	2	3	4	5	6	7

Question Code: 8X17X126
Short Desc: 14GET

Do you currently GET support from your family or friends in getting enough physical activity?
Choose only one of the following

- Yes (yes)
- No (no)

Question Code: 8X17X127
Short Desc: 15WANT

Do you WANT support from your family or friends in getting enough physical activity?
Choose only one of the following

- Yes (yes)
- No (no)

Question Code: 8X17X128
Short Desc: 16WHO

Section 3

Question Code: 8X18X129
Short Desc: 17dog

Do you own a dog?
Choose only one of the following

- Yes (yes)
- No (no)

Question Code: 8X18X130
Short Desc: 18walk
Question Code: 8X18X131
Short Desc: 19time

Section 4

Question Code: 8X19X132
Short Desc: 20ratehealth

In general, how would you rate your health?
Choose only one of the following

- Excellent (exc)
- Very good (vgood)
- Good (good)
- Fair (fair)
- Poor (poor)

Question Code: 8X19X133
Short Desc: 21limits

Please rate how much your health currently limits you in the following activities.

	Not at all Limited	Somewhat Limited	Significantly Limited
Low intensity activities, such as casual walking, golf, home maintenance, or gardening 8X19X133Low	NoLimit	SomeLimit	SigLimit
Moderate intensity activities, such as racquet sports, pushing a vacuum cleaner, bowling, golf, bicycling, swimming, or fast walking 8X19X133Mod	NoLimit	SomeLimit	SigLimit
Climbing one flight of stairs 8X19X133Clim	NoLimit	SomeLimit	SigLimit

Climbing several flights of stairs 8X19X133Clims	NoLimit	SomeLimit	SigLimit

Question Code: 8X19X134
Short Desc: 22feel

For each question below, please give the one answer that comes closest to the way you have been feeling in the last 4 weeks. How much of the time:

	**None of the time**	**A little of the time**	**A good bit of the time**	**Most of the time**	**All of the time**
Have you felt calm and peaceful? 8X19X134calm	1	2	3	4	5
Did you have a lot of energy? 8X19X134energy	1	2	3	4	5
Have you felt downhearted and blue? 8X19X134down	1	2	3	4	5

Question Code: 8X19X135
Short Desc: 23belief

Each item below is a belief statement about your health with which you may agree or disagree. Please choose a response along the scale that best represents how you feel.

	**1 - Strongly disagree**	**2**	**3**	**4**	**5**		**7 - Strongly agree**
The main thing that affects my health is what I myself do. 8X19X135myself	1	2	3	4	5	6	7
If I take care of myself, I can avoid illness. 8X19X135avoid	1	2	3	4	5	6	7
If I take the right	1	2	3	4	5	6	7

actions, I can stay healthy.
8X19X135action

Question Code: 8X19X136
Short Desc: 24lose

Are you currently trying to lose weight?
Choose only one of the following

- Yes (yes)
- No (no)

Question Code: 8X19X137
Short Desc: 25cigs

Do you currently smoke cigarettes?
Choose only one of the following

- Yes (yes)
- No, but I am a former smoker (noformer)
- No, I have never smoked (nonever)

Question Code: 8X19X138
Short Desc: 26numbercigs
Question Code: 8X19X139
Short Desc: 27quit

Section 5

Question Code: 8X28X181
Short Desc: 28resp

Please respond to the following statements:

	**Strongly Disagree**				**Strongly Agree**
I found Stepping Up to Health useful. 8X28X181useful	D	b	c	d	e
Using Stepping Up to Health enables me to monitor my walking more quickly. 8X28X181monitor	D	b	c	d	e

Using Stepping Up to Health increases the amount I walk. 8X28X181increase	D	b	c	d	e
My interaction with Stepping Up to Health was clear and understandable. 8X28X181interact	D	b	c	d	e
It would be easy for me to become skillful at using Stepping Up to Health. 8X28X181skill	D	b	c	d	e
I found Stepping Up to Health easy to use. 8X28X181easy	D	b	c	d	e
Learning to operate Stepping Up to Health is easy for me. 8X28X181operate	D	b	c	d	e
Using Stepping Up to Health is a bad idea. 8X28X181bad	D	b	c	d	e
Stepping Up to Health makes physical activity more interesting. 8X28X181interest	D	b	c	d	e
Working with Stepping Up to Health is fun. 8X28X181fun	D	b	c	d	e
I like working with Stepping Up to Health. 8X28X181like	D	b	c	d	e

I have the resources necessary to use Stepping Up to Health. 8X28X181resource	D	b	c	d	e
I have the knowledge necessary to use Stepping Up to Health. 8X28X181know	D	b	c	d	e
Stepping Up to Health is not compatible with other systems I use. 8X28X181sys	D	b	c	d	e
A specific person (or group) is available for assistance with Stepping Up to Health difficulties. 8X28X181assist	D	b	c	d	e
I feel apprehensive about using Stepping Up to Health. 8X28X181app	D	b	c	d	e
It scares me to think that I could lose a lot of information using Stepping Up to Health by hitting the wrong key. 8X28X181scare	D	b	c	d	e
I hesitate to use Stepping Up to Health for fear of making mistakes I cannot correct. 8X28X181hesitate	D	b	c	d	e
Stepping Up to	D	b	c	d	e

Health is somewhat intimidating to me. 8X28X181intimid

Question Code: 8X28X182
Short Desc: 29comp

I could complete a job or task using Stepping Up to Health...

	Strongly Disagree				Strongly Agree
If there was no one around to tell me what to do as I go. 8X28X182none	D	b	c	d	e
If I could call someone for help if I got stuck. 8X28X182someone	D	b	c	d	e
If I had a lot of time to complete the job for which Stepping Up to Health was provided. 8X28X182time	D	b	c	d	e
If I had just the built-in help facility for assistance. 8X28X182facility	D	b	c	d	e

Section 6

Post-Survey Study Satisfaction Survey

Question Code: 8X27X172
Short Desc: 2graphs

The graphs of my walking on the Stepping Up to Health website were:

Choose only one of the following

- Very useful (a)
- Somewhat useful (b)
- Not very useful (c)
- Not useful at all (d)

Question Code: 8X27X173
Short Desc: 3diabetes

I would recommend Stepping Up to Health to a friend with diabetes.
Choose only one of the following

- Very useful (a)
- Somewhat useful (b)
- Not very useful (c)
- Not useful at all (d)

Question Code: 8X27X174
Short Desc: 4cad

I would recommend Stepping Up to Health to a friend with coronary artery disease.
Choose only one of the following

- Definitely (def)
- Maybe (b)
- Probably not (c)
- Definitely not (d)

Question Code: 8X27X175
Short Desc: 5overweight

I would recommend Stepping Up to Health to a friend who is overweight.
Choose only one of the following

- Definitely (def)
- Maybe (b)
- Probably not (c)
- Definitely not (d)

Question Code: 8X27X176
Short Desc: 6three

What three things did you enjoy the MOST about Stepping Up to Health?

Question Code: 8X27X177
Short Desc: 7least

What three things did you enjoy LEAST about Stepping Up to Health?

Question Code: 8X27X178
Short Desc: 8pay

I would consider paying up to $________ to be able to continue using something like the Stepping Up to Health program for one year.

SEE QUSTIONCODE ABOVE

Question Code: 8X27X179
Short Desc: 9other

Other comments.

next >>

[Exit and Clear Survey]
