# Supplementary material for: An Online Community Improves Adherence in an Internet-Mediated Walking Program. Part 1: Results of a Randomized Controlled Trial
Source: J Med Internet Res. 2010 Dec 17;12(4):e71. doi: 10.2196/jmir.1338 (PMC3056526; doi:10.2196/jmir.1338)
Supplement: Supplementary file 9 [file jmir_v12i4e71_app9.html]

WG1.html 


Stepping Up To Health - Web Session 1 - Pages 1 and 2 - Last Revised August 10, 2006

|  |  |  |  |
| --- | --- | --- | --- |
| **Command** | **Logic** | **Message** | **Row** |
| Comment | WG1.html | Goal of Section: Welcome everyone to the program. Share with them what the program is about, what they will recieve, what we understand about them, and what they can find on the website. | 10 |
| Section | Page1Header |  | 20 |
| Text | not isEmpty(AddressPref) | **Welcome $AddressPref!** | 30 |
| Text | isEmpty(AddressPref) | **Welcome!** | 40 |
| Section | Page1Body |  | 50 |
| Text |  | We're very happy you have chosen to participate in **Stepping Up To Health**! | 60 |
| Comment |  | Text that could fire for InternalMotList\_Text: prevent future health problems, take responsibility for your own health, manage your weight, improve your ability to do daily activities, increase your energy levels, improve your physical health. (Depending on their answers to the survey, one or two from the list will appear. If two fire, they will be seperated with the word and.) | 70 |
| Text | count(InternalMotList)>=1 | You shared with us that you want to increase how much you walk because $englishList(InternalMotList,1,2). | 80 |
| Text |  | There's no doubt about it, **walking is good for you**. It's good for your heart, your lungs, for muscle and bone growth, and for your feeling of well-being. Strong scientific evidence supports the many benefits of regular walking. | 100 |
| Paragraph |  |  | 110 |
| Text |  | **Stepping Up To Health** is designed to help you increase your daily steps. We'll help you work through obstacles that you feel make it hard for you to regularly walk. We'll also discuss how to get support from those around you. How have you been doing so far? Have you begun walking more since joining the program? Are you wearing your pedometer right now? | 120 |
| Paragraph |  |  | 130 |
| Select | 1 |  | 131 |
| Text | Gender=="Male" | For a **man in his** | 140 |
| Text | Gender=="Female" | For a **woman in her** | 150 |
| Text | isEmpty(Gender) | For a person in **your** | 160 |
| EndSelect |  |  | 161 |
| Select | 1 |  | 162 |
| Text | Age>1 and Age<20 | **teens,** | 163 |
| Text | Age>=20 and Age<30 | **20's,** | 164 |
| Text | Age>=30 and Age<40 | **30's,** | 165 |
| Text | Age>=40 and Age<50 | **40's,** | 166 |
| Text | Age>=50 and Age<60 | **50's,** | 170 |
| Text | Age>=60 and Age<70 | **60's,** | 180 |
| Text | Age>=70 and Age<80 | **70's,** | 190 |
| Text | Age>=80 and Age<90 | **80's,** | 200 |
| Text | Age>=90 and Age<100 | **90's,** | 210 |
| Text | Age>=100 | **100's,** | 220 |
| Text | isEmpty(Age) | As we move forward, think about how | 230 |
| EndSelect |  |  | 231 |
| Text |  | increasing your daily steps can greatly benefit your health, especially | 240 |
| Comment |  | Text that could fire for WalkHealthHistory\_Text: arthritis, osteoporosis, emotional condition, sleep problems, chronic pain, high blood pressure, high cholesterol. (Depending on their answers to the survey, one or two from the list will appear. If two fire, they will be seperated with the word and.) | 241 |
| Select | 1 |  | 250 |
| Text | CADSev in ("High", "Low") | for someone like you who **has coronary artery disease.** | 260 |
| Text | DIABSev in ("High", "Low") | for someone like you who **has diabetes**. | 270 |
| Text | OBSev in ("High", "Low") | for someone like you who **is overweight**. | 280 |
| Text | count(WalkHealthHistory)>=1 | to help your $englishList(WalkHealthHistory, 1,2). | 300 |
| Text |  | to help you avoid future chronic conditions. | 310 |
| EndSelect |  |  | 320 |
| Select | 1 |  | 330 |
| Text | "CVD" in FamilyHealthHist | Plus, with your **family history of heart disease**, becoming more physically active might be extra meaningful to you. | 340 |
| Text | "Diabetes" in FamilyHealthHist and DIABSev in ("High", "Low") | Plus, with your **family history of diabetes**, becoming more physically active might be extra meaningful to you. | 341 |
| Text | "Diabetes" in FamilyHealthHist | Plus, with your **family history of diabetes**, becoming more physically active might be extra meaningful to you. | 350 |
| Text | "Stroke" in FamilyHealthHist | Plus, since someone in your family has **suffered a stroke** (family history is associated with an increased risk for you), becoming more physically active might be extra meaningful to you. | 360 |
| Text | "HiChol" in FamilyHealthHist | Plus, with your **family history of high cholesterol**, becoming more physically active might be extra meaningful to you. | 370 |
| Text | "HBP" in FamilyHealthHist | Plus, with your **family history of high blood pressure**, becoming more physically active might be extra meaningful to you. | 380 |
| Text | "Osteo" in FamilyHealthHist | Plus, with your **family history of osteoporosis**, becoming more physically active might be extra meaningful to you. | 390 |
| Text | "BreastCancer" in FamilyHealthHist or "ColonCancer" in FamilyHealthHist or "ProstateCancer" in FamilyHealthHist or "OtherCancer" in FamilyHealthHist | Plus, since someone in your family has experienced **cancer**, becoming more physically active might be extra meaningful to you. | 400 |
| EndSelect |  |  | 410 |
| Paragraph |  |  | 420 |
| Text |  | We'd like to begin by reviewing what we learned about you from your survey responses: | 430 |
| Select | 1 |  | 440 |
| Text | OwnDog=="Yes" and WalkDog=="Yes" and OftenWalkDog=="15Less" | - You have a walking partner that will walk with you every day -- **your dog**! You told us that you walk your dog about 15 minutes a day. A few extra walks each day is a great way for you to get outside and get some extra steps. | 450 |
| Text | OwnDog=="Yes" and WalkDog=="Yes" and OftenWalkDog=="15to30Min" | - You have a walking partner that will walk with you every day -- **your dog**! You told us that you walk your dog about 15-30 minutes a day. A few extra walks each day is a great way for you to get outside and get some extra steps. | 460 |
| Text | OwnDog=="Yes" and WalkDog=="Yes" and OftenWalkDog=="31to60Min" | - You have a walking partner that will walk with you every day -- **your dog**! You told us that you walk your dog about 30-60 minutes a day. That's great! Walking your dog is a great way to meet your daily walking goal. | 470 |
| Text | OwnDog=="Yes" and WalkDog=="Yes" and OftenWalkDog=="MoreThan60Min" | - You have a walking partner that will walk with you every day -- **your dog**! You told us that you walk your dog more than an hour a day. That's great! Walking your dog is a great way to meet your daily walking goal. | 480 |
| Text | OwnDog=="Yes" and WalkDog=="Yes" | - You have a walking partner that will walk with you every day -- **your dog**! You told us that you already walk your dog, but a few extra walks each day is a great way for you to get outside and get some extra steps. | 490 |
| Text | OwnDog=="Yes" and WalkDog=="No" | - You have a walking partner that will walk with you every day -- **your dog**! One or two short walks each day is a great way for you to get outside and get some extra steps. | 500 |
| Text | OwnDog=="Yes" | - You have a walking partner that will walk with you every day -- **your dog**! Walking your dog is a great way for you to get outside and get some extra steps into your day. | 510 |
| EndSelect |  |  | 520 |
| Comment |  | Text that could fire for PersonalRecAct\_Text: bowling, gardening, golfing, shuffleboard, reading, watching television, bicycling, fishing, tennis, swimming, playing bingo, playing cards, church or social clubs, spending time with friends. (Depending on their answers to the survey - one, two, or three from the list will appear. If two fire, they will be seperated with the word and. If three fire, they will be seperated with a comma and the word and.) | 521 |
| Select | 2 |  | 530 |
| Text | YrsAgoQuit in ("Less1Yr", "1to2Yr", "2to5Yr", "More5Yr") | - **You're a former smoker.** Congratulations on quitting! That's a tough challenge that you've overcome. You know what it takes to make big changes in your life. This can help you as you work to increase your daily steps. | 540 |
| Text | count(EnjoyRecAct)>=1 and "None" not in EnjoyRecAct | - You currently enjoy recreational activities, including $englishList(EnjoyRecAct, 1,3). What will it take to add walking to that list? | 560 |
| Text | CurrentSupport=="Yes" | - You mentioned that your family and friends are a **great source of support**. As you get into your walking program, you'll be happy you have the support of people who care about you. | 570 |
| Text | TryingLoseWt=="Yes" | - You told us that you're **trying to lose weight.** Walking is a great way to burn off some of those extra pounds. | 580 |
| Text | OAMotWalk>7 | - You're **really motivated** to walk every day. You're in a great place as you start this program. | 590 |
| Text | OAMotWalk>=4 and OAMotWalk<=7 | - You're feeling **pretty motivated** to walk each day. | 600 |
| Text | GenHealth in ("Fair", "Poor") | - You don't feel like your health is the best that it can be. By choosing to walk more, you may find your feelings will change over time. | 610 |
| Text | OAConWalk>7 | - You're **really confident** that you can walk every day. | 620 |
| Text | OAConWalk>=4 and OAConWalk<=7 | - You feel **pretty confident** about being able to walk more. We can help build that confidence. | 630 |
| Text | SF36SevFlights in ("SomeLimit", "SigLimit") or SF36OneFlight in ("SomeLimit", "SigLimit") | - You have some **difficulty climbing stairs**. Over time, walking may actually help make those stairs a little easier. | 640 |
| Text |  | - **You and your doctor** both feel that walking is a great way to start helping you become more active. | 650 |
| Text |  | - You signed up for **this program**. You're ready to take charge of your health. | 660 |
| EndSelect |  |  | 670 |
| Paragraph |  |  | 680 |
| Text |  | Research shows that **exercise is a powerful defender of our health.** What can it do for you? | 690 |
| Select | 1 |  | 700 |
| Text | CADSev in ("High", "Low") | - Living with **heart disease** can be difficult. But research shows that regular exercise can reduce your future cardiovascular risks. Walking is a great way to strengthen the strongest muscle in your body -- your heart. | 710 |
| Text | DIABSev in ("High", "Low") | - **Dealing with diabetes** everyday can be difficult. But research shows that regular exercise can help people with diabetes. Walking can help your insulin work better, improve your blood circulation, lower your blood sugar, and keep your joints flexible. | 720 |
| EndSelect |  |  | 730 |
| Select | 3 |  | 740 |
| Text | not DIABSev in ("High", "Low") and not CADSev in ("High", "Low") and not "BreastCancer" in HealthHistory and not "ColonCancer" in HealthHistory and not "ProstateCancer" in HealthHistory and not "LungCancer" in HealthHistory and not "OtherCancer" in HealthHistory and not "HBP" in HealthHistory and not "Arthritis" in HealthHistory | - As someone who is currently **overweight**, you are at risk for diabetes, heart disease, certain types of cancer, hypertension, and arthritis. Exercise will not only reduce your chance of being diagnosed with a serious disease, but along with a healthy diet, exercise is the best way to improve your blood circulation and reduce the pressure on your bones. | 750 |
| Text | not DIABSev in ("High", "Low") | - A great way to reduce your risk of **developing diabetes** is to exercise regularly and eat a healthy diet. | 760 |
| Text | "HBP" in HealthHistory | - Having **high blood pressure** makes your heart work harder and causes your arteries to be under added strain. By walking, you can reduce your blood pressure. Even a small amount of regular activity can help reduce long-term consequences of high blood pressure. | 770 |
| Text | "Arthritis" in HealthHistory | - **Arthritis** can be hard to live with. Exercise can help relieve the pain and stiffness you might feel. It can also give you more energy and reduce the disability common with arthritis. | 780 |
| Text | Gender=="Female" and Age>=45 | - Exercise can have positive effects on several **menopausal symptoms**. It can decrease the number and severity of hot flashes and reduce weight gain. It can also decrease anxiety and depression, increase energy levels, and reduce the progression of osteoporosis. | 790 |
| Text | Gender=="Male" and Age>=45 | - There is good news for men who need another reason to become motivated to walk! Men who exercise most days of the week have 30% less risk of experiencing **erectile dysfunction** and impotence. | 800 |
| Text | SmokeCigs=="Yes" | - You shared with us that you smoke. Smokers who are unable to quit can **still improve their health** by becoming physically active. And if you do decide that you might want to quit, physical activity can help! It's a great stress reliever that can replace reaching for a cigarette. | 810 |
| Text | TryingLoseWt=="Yes" | - Walking is a great way to **lose weight**. Walking one mile can burn at least 100 calories. By walking two miles a day, three times a week, you can help reduce your weight by one pound every three weeks. | 820 |
| Text | "Osteo" in HealthHistory | - **Osteoporosis** occurs when the bones lose calcium and become fragile. Walking can help promote bone growth and bone density. Physical activity reduces your risk of both falling and breaking a bone. | 830 |
| Text | Age>=50 | - Walkers have **stronger bones** and more flexibility than people who don't walk. As a walker, you're less likely to fall and suffer injuries such as hip fractures or joint injuries. Walkers also have much better balance than other people of the same age who do not walk. | 840 |
| Text | Age>1 and Age<50 | - Walkers have **stronger bones** and more flexibility than people who don't walk. As a walker, you'll also strengthen your muscles and joints. Walkers also have much better balance than other people of the same age who do not walk. |  |
| Text |  | - Walking has been shown to improve self esteem, relieve symptoms of depression and anxiety, and improve mood. Walking, particularly in pleasant surroundings, and with other people, offers many opportunities for **relaxation and social contact.** | 850 |
| EndSelect |  |  | 860 |
| Paragraph |  |  | 870 |
| Text |  | Exercising takes effort, but you can succeed with **careful planning and patience**. | 880 |
| Block | "SeatBelt" in OtherBeh or "BicHelmet" in OtherBeh or "MotorHelmet" in OtherBeh or "Physical" in OtherBeh or "StressManage" in OtherBeh or "Floss" in OtherBeh or "FluShot" in OtherBeh or "MultiVit" in OtherBeh or "Vacc" in OtherBeh or SmokeCigs in ("NoNever", "NoFormer") |  | 890 |
| Text |  | Just think of what you already do for your health. | 900 |
| Select | 3 |  | 910 |
| Text | "StressManage" in OtherBeh | - You actively try to manage your stress. | 920 |
| Text | SmokeCigs in ("NoNever", "NoFormer") | - You don't smoke. | 930 |
| Text | "SeatBelt" in OtherBeh | - You wear a seatbelt in the car. | 940 |
| Text | "BicHelmet" in OtherBeh | - You wear a bike helmet while cycling. | 950 |
| Text | "MotorHelmet" in OtherBeh | - You wear a motorcylce helmet while riding. | 960 |
| Text | "Physical" in OtherBeh | - You see a healthcare provider regularly. | 970 |
| Text | "Floss" in OtherBeh | - You floss your teeth. | 980 |
| Text | "FluShot" in OtherBeh | - You get a flu shot every year. | 990 |
| Text | "MultiVit" in OtherBeh | - You take a multivitamin. | 1000 |
| Text | "Vacc" in OtherBeh | - You're up to date on your shots. | 1010 |
| Text |  | - You signed up for this program! | 1020 |
| EndSelect |  |  | 1030 |
| Paragraph |  |  | 1040 |
| EndBlock |  |  | 1050 |
| Select | 1 |  | 1060 |
| Text | containsOne (OtherBeh, ["SeatBelt", "BicHelmet", "MotorHelmet", "Physical", "StressManage", "Floss", "FlusShot", "MultiVit", "Vacc"]) or SmokeCigs in ("NoNever", "NoFormer") | Walking will be one more thing you can do to maintain -- and improve -- your health. Continue on to the next page to learn more about why walking is recommended for people | 1070 |
| Text |  | Now is the time to increase your effort to take care of your body. Continue on to the next page to learn more about why walking is recommended for people | 1080 |
| EndSelect |  |  | 1090 |
| Select | 1 |  | 1100 |
| Text | CADSev in ("High", "Low") | with heart disease. | 1110 |
| Text | DIABSev in ("High", "Low") | with diabetes. | 1120 |
| Text | OBSev in ("High", "Low") | who are overweight. | 1130 |
| Text |  | who want to start exercising. | 1140 |
| EndSelect |  |  | 1150 |
| Section | Page2Header |  | 2020 |
| Text |  | **It's time to get moving!** | 2030 |
| Section | Page2Body |  | 2040 |
| Text |  | We're really glad you got your doctor's approval to start this program. Now that you're ready to get going, you should know a little more about exercise safety before you begin. We don't want you to get hurt. | 2050 |
| Paragraph |  |  | 2060 |
| Text |  | **How can I prevent pain and injury?** | 2070 |
| Paragraph |  |  | 2080 |
| Text |  | - When you begin, don't push yourself too hard. **Start slowly**. Ease into your walk. - Walk a little further than you normally do and **work up to walking further and faster** as time passes. - While you might be breathing hard, you should still be able to talk to a friend while walking. - After walking, **cool down** by slowing down gradually. Make sure to stretch after walking. Stretching after walking can help prevent muscle pain the next day. - If you ever feel pain while walking, be sure to visit your doctor before continuing. | 2090 |
| Paragraph |  |  | 2100 |
| Text |  | **Why should I walk?** | 2110 |
| Paragraph |  |  | 2120 |
| Select | 1 |  | 2121 |
| Text | CADSev=="High" | Based on your survey responses, it sounds like your **heart disease** really impacts your life. | 2140 |
| Text | DIABSev=="High" | Based on your survey responses, it sounds like your **diabetes** really impacts your life. | 2150 |
| Text | CADSev=="Low" | Based on your survey responses, having **heart disease** doesn't impact your life as much as it does others. | 2160 |
| Text | DIABSev=="Low" | Based on your survey responses, having **diabetes** doesn't impact your life as much as it does others. | 2170 |
| Text | OBSev=="High" | Based on your survey responses, it sounds like your weight really impacts your life. | 2180 |
| Text | OBSev=="Low" | Based on your survey responses, we know that you are **overweight**, but it sounds like your weight doesn't limit your daily activities. | 2190 |
| EndSelect |  |  | 2191 |
| Select | 1 |  | 2210 |
| Text | CADSev == "High" | People with heart disease should make a point to walk every day because walking can actually make your heart healthier.  - Walking can reduce feelings of chest pain and pressure. - It can **strengthen your heart** and help regulate your heartbeat. - Walking can reduce nausea and weakness. | 2220 |
| Text | DIABSev=="High" | People with diabetes should make a point to walk every day because walking can help you better manage your condition.  - Walking can lower your blood glucose and blood pressure. - It will lower your bad cholesterol and raise your good cholesterol. - It will improve your body's **ability to use insulin**. - Walking can help you lose weight. | 2230 |
| Text | CADSev=="Low" | People with heart disease should make a point to walk every day because walking can actually make your heart healthier.  - Walking can reduce feelings of chest pain and pressure. - It can **strengthen your heart** and help regulate your heartbeat. - Walking can reduce nausea and weakness. | 2240 |
| Text | DIABSev=="Low" | People with diabetes should make a point to walk every day because walking can help you better manage your condition.  - Walking can lower your blood glucose and blood pressure. - It will lower your bad cholesterol and raise your good cholesterol. - It will improve your body's **ability to use insulin**. - Walking can help you lose weight. | 2241 |
| Text | OBSev in ("High", "Low") | Overweight people should make a point to walk every day because walking can help you:  - Lower your blood pressure. - **Reduce depression** and help you sleep better. - Lose weight. - Increase your self-esteem. | 2242 |
| Text | Age>=50 | People your age should make a point to walk every day because the older you are, the more you need regular exercise. Exercise is the best way to **strengthen your heart**.  - Walking helps build and maintain healthy bones, muscles and joints. - It can help control weight. - Regular participation in walking is associated with reduced mortality rates for older adults. In other words, **walkers live longer**! | 2243 |
| Text |  | Everyone should make a point to walk daily because of all the great benefits.   - Walking helps build and maintain healthy bones, muscles and joints. - It can help control weight. - It may reduce depression and help you sleep better. - Walking is a great way to increase your self-esteem. | 2250 |
| EndSelect |  |  | 2251 |
| Paragraph |  |  | 2252 |
| Text |  | **How can people** | 2253 |
| Select | 1 |  | 2254 |
| Text | CADSev in ("High", "Low") | **with heart disease start a walking program?** | 2260 |
| Text | DIABSev in ("High", "Low") | **with diabetes start a walking program?** | 2270 |
| Text | OBSev in ("High", "Low") | **my weight start a walking program?** | 2280 |
| Text |  | **who want to be more active start a walking program?** | 2290 |
| EndSelect |  |  | 2300 |
| Paragraph |  |  | 2310 |
| Text |  | The Surgeon General recommends that | 2320 |
| Select | 1 |  | 2330 |
| Text | CADSev in ("High", "Low") | people with heart disease | 2340 |
| Text | DIABSev in ("High", "Low") | people with diabetes | 2350 |
| Text | OBSev in ("High", "Low") | people who are overweight | 2360 |
| Text |  | everyone should | 2370 |
| EndSelect |  |  | 2380 |
| Text |  | **exercise at least most, if not all days of the week.** That's been interpreted by most people as 5 or more days of the week. If you miss a day, it's ok. You're just getting started. You're not going to make your goal every day. If you get sick or can't walk for a few days, just go back when you can. You know your body. Test yourself. Over time, slowly increase the intensity of your workout and the duration. **Your final goal is to walk every day**, but take the time you need to get there. | 2390 |
| Paragraph |  |  | 2420 |
| Select | 1 |  | 2430 |
| Text | CADSev in ("High", "Low") | Going on walks is not the only way to increase your number of steps. You may not realize activities like golf, dancing, raking, mowing, gardening, and vacuuming are a great way to get in those extra steps. They're fine for almost anyone with a heart related condition. | 2440 |
| Text | DIABSev in ("High", "Low") and DiabFtProb>=4 | Knowing that you have foot problems, you want to pick exercises that won't put a lot of stress on your feet. Walking is a great way to begin, but it is not the only way to increase your number of steps. You may not realize that activities like golf, raking, mowing, gardening, vacuuming, and chair exercise are a great way to get in those extra steps. | 2450 |
| Text |  | Walking is a great way to begin, but it is not the only way to increase your number of steps. You may not realize that activities like golf, raking, mowing, gardening, vacuuming, and chair exercise are a great way to get in those extra steps. | 2460 |
| EndSelect |  |  | 2470 |
| Paragraph |  |  | 2670 |
| Text |  | **Are there any exercises that I should stay away from?** | 2680 |
| Paragraph |  |  | 2690 |
| Select | 1 |  | 2700 |
| Text | CADSev=="High" | Since your **heart disease** has impacted your life fairly significantly over the past year, you'll want to start your walking program slowly. Increase your daily steps, but don't push yourself too hard. | 2710 |
| Text | DIABSev=="High" | Since your **diabetes** has impacted your life fairly significantly over the past year, you'll want to start your walking program slowly. Increase your daily steps, but don't push yourself too hard. | 2720 |
| Text | OBSev=="High" | Since your **weight** has impacted your life fairly significantly over the past year, you'll want to start your walking program slowly. Perhaps you have pain in your back, knees or joints. We understand that beginning to exercise may be painful or hard for you at first. Increase your daily steps, but don't push yourself too hard. | 2730 |
| Text | CADSev=="Low" | While you have **heart disease**, your condition hasn't significantly impacted your life over the past year. However, you still want to be cautious when you begin an exercise routine. Start your walking program slowly. As time goes on, continue increasing your daily steps, and push yourself a little harder. | 2740 |
| Text | DIABSev=="Low" | While you have **diabetes**, your condition hasn't significantly impacted your life over the past year. However, you still want to be cautious when you begin an exercise routine. Start your walking program slowly. As time goes on, continue increasing your daily steps, and push yourself a little harder. | 2750 |
| Text | OBSev=="Low" | While you are **overweight**, your weight hasn't significantly impacted your life over the past year. However, you still want to be cautious when you begin an exercise routine. Start your walking program slowly. As time goes on, continue increasing your daily steps, and push yourself a little harder. | 2760 |
| EndSelect |  |  | 2761 |
| Select | 1 |  | 2762 |
| Text | DIABSev in ("High", "Low") | Remember, if something hurts or you feel discomfort, stop walking and take a break. Other things to keep in mind:  - Nerve damage can make it hard to tell if you've injured your feet during exercise. Check your feet before and after exercise for sores, blisters, irritation, cuts, or other injuries. - Walking may lower your blood glucose too much which can cause hypoglycemia. Be sure to check your blood glucose before you exercise. If it's below 100, have a small snack. After you exercise, check to see how it has affected your blood glucose level. - Drink plenty of fluids while walking, since your blood glucose can be affected by dehydration. | 2764 |
| Text | CADSev in ("High", "Low") or OBSev in ("High", "Low") | Remember, if something hurts or you feel discomfort, stop walking and take a break. | 2765 |
| Text | Age>=50 | Due to your age, there are a few special precautions you should take as you begin to walk more.  - Don't try to do too much too fast. - Exercise at an intensity appropriate for you. - Pick activities that are fun, that suit your needs and that you can do year-round. - Take more time to warm up and cool down before and after your workout. | 2770 |
| Text |  | There are a few special precautions you should take as you begin to walk more.   - Don't try to do too much too fast. - Exercise at an intensity appropriate for you. - Pick activities that are fun, that suit your needs and that you can do year-round. - Take the time to wam up and cool down before and after your workout. | 2271 |
| EndSelect |  |  | 2780 |
| Paragraph |  |  | 2790 |
| Text |  | **What to expect next** | 2810 |
| Paragraph |  |  | 2820 |
| Text | not isEmpty(AddressPref) | $AddressPref, in one week | 2830 |
| Text | isEmpty(AddressPref) | In one week, | 2840 |
| Text |  | you will receive an email message from **Stepping Up To Health** letting you know that your second personalized Web session is available. The session will focus on how to deal with concerns you have about being able to regularly include walking in your day. | 2850 |
| Paragraph |  |  | 2860 |
| Text |  | In fact, you will receive an email message from **Stepping Up To Health** whenever it's time to visit the Web site for new sessions, or when it's time to upload your pedometer. Of course, you can visit this site whenever you wish as well. Every day, this site will have a new **Daily Tip** that may help you move closer to your daily step goal. You can also review your weekly goal and your progress charts. | 2870 |
| Paragraph |  |  | 2880 |
| Text |  | Thank you for joining **Stepping Up To Health**. Best wishes as you begin to increase your daily steps and take control of your health. Remember, every small step you take to improve your exercise habits is one step closer to being healthier for the rest of your life. | 2890 |
