# Supplementary material for: An Online Community Improves Adherence in an Internet-Mediated Walking Program. Part 1: Results of a Randomized Controlled Trial
Source: J Med Internet Res. 2010 Dec 17;12(4):e71. doi: 10.2196/jmir.1338 (PMC3056526; doi:10.2196/jmir.1338)
Supplement: Supplementary file 10 [file jmir_v12i4e71_app10.html]

WG2.html


SUH - Session 2, pages 1 and 2 - Last Revision August 17, 2006

|  |  |  |  |
| --- | --- | --- | --- |
| **Command** | **Logic** | **Message** | **Row** |
| Comment | WG2.html | **Goal of Session #2:** Provide solutions for the participant's two biggest barriers to walking. | 10 |
| Section | Page1Header |  | 20 |
| Evaluate |  | Barrier = Barrier1 | 1000 |
| Text |  | **Things that can get in the way of walking . . .** | 30 |
| Section | Page1Body |  | 40 |
| Text | not isEmpty(AddressPref) | $AddressPref, welcome | 50 |
| Text | isEmpty(AddressPref) | Welcome | 60 |
| Text |  | back to your second personalized web guide from **Stepping Up To Health.**  In this session, we'll identify some of the specific challenges that many people face when it comes to walking and we'll offer some tips and suggestions for how you can overcome those challenges. | 70 |
| Paragraph |  |  | 80 |
| Select | 1 |  | 90 |
| Text | len(WalkBarrierVeryTrue)>=2 | Like many others, you have **some specific concerns** about being able to increase your daily steps. | 100 |
| Text | len(WalkBarrierVeryTrue)==1 or len(WalkBarrierTrue)>=1 | While you don't have serious concerns about being able to increase your daily steps, **you do have a few specific issues** that you feel could potentially get in the way. | 110 |
| Text |  | You **don't seem to have many barriers** about being able to increase your daily steps. Still, we want to provide you with some ideas on how others have worked through two of the more common issues that make daily walking hard for them. | 120 |
| EndSelect |  |  | 130 |
| Block | count(WalkBarrierVeryTrue)>=1 or count(WalkBarrierTrue)>=1 |  | 140 |
| Select | 1 |  | 150 |
| Text | OAConWalk>=8 | The good news is -- **overall, you feel very confident** you can do it. To help you maintain that overall confidence, let's look at those specific issues you feel make it hard for you to walk more and solutions to get past them. | 160 |
| Text | OAConWalk>=5 and OAConWalk<=7 | The good news is -- **overall, you feel pretty confident** you can do it. To help you boost that overall confidence a little more, let's look at those specific issues you feel make it hard for you to walk more and solutions to get past them. | 170 |
| Text | OAConWalk>=1 and OAConWalk<=4 | Your first challenge will be to increase your **overall confidence level**. To help you do that, let's look at those specific issues you feel make it hard for you to walk more and solutions to get past them. | 180 |
| Text |  | Let's take a look at some of those issues you feel might make it hard for you and solutions to get past them. | 190 |
| EndSelect |  |  | 200 |
| EndBlock |  |  | 210 |
| Paragraph |  |  | 220 |
| Select | 1 |  | 230 |
| Text | count(WalkBarrierVeryTrue)>=1 or count(WalkBarrierTrue)>=1 | You shared with us that you might find it tough to walk, especially when | 240 |
| Text |  | Many people don't feel confident that they can walk, especially when | 250 |
| EndSelect |  |  | 260 |
| Select | 1 |  | 280 |
| Text | "BarTime" in Barrier1 and (count(WalkBarrierVeryTrue)>=1 or count(WalkBarrierTrue)>=1) | you don't feel you have the time. | 290 |
| Text | "BarTime" in Barrier1 | they don't feel they have the time. | 300 |
| Text | "BarHealthProb" in Barrier1 | health issues make it difficult. | 310 |
| Text | "BarTired" in Barrier1 and (count(WalkBarrierVeryTrue)>=1 or count(WalkBarrierTrue)>=1) | you feel tired. | 320 |
| Text | "BarTired" in Barrier1 | they feel tired. | 330 |
| Text | "BarEnergy" in Barrier1 and (count(WalkBarrierVeryTrue)>=1 or count(WalkBarrierTrue)>=1) | you don't have the energy. | 350 |
| Text | "BarEnergy" in Barrier1 | they don't have the energy. | 360 |
| Text | "BarHurt" in Barrier1 and (count(WalkBarrierVeryTrue)>=1 or count(WalkBarrierTrue)>=1) | you're afraid of getting hurt. | 370 |
| Text | "BarHurt" in Barrier1 | they're afraid of getting hurt. | 380 |
| Text | "BarEffort" in Barrier1 | it seems to take too much effort. | 400 |
| Text | "BarBadMood" in Barrier1 and (count(WalkBarrierVeryTrue)>=1 or count(WalkBarrierTrue)>=1) | you're in a bad mood. | 410 |
| Text | "BarBadMood" in Barrier1 | they're in a bad mood. | 420 |
| Text | "BarExpensive" in Barrier1 | it's too expensive to join a club or gym. | 430 |
| Text | "BarSafePlace" in Barrier1 and (count(WalkBarrierVeryTrue)>=1 or count(WalkBarrierTrue)>=1) | you don't feel you have a safe place to exercise. | 440 |
| Text | "BarSafePlace" in Barrier1 | they don't feel they have a safe place to exercise. | 450 |
| Text | "BarSweat" in Barrier1 and (count(WalkBarrierVeryTrue)>=1 or count(WalkBarrierTrue)>=1) | you might sweat. | 460 |
| Text | "BarSweat" in Barrier1 | they might sweat. | 470 |
| Text | "BarNoOne" in Barrier1 and (count(WalkBarrierVeryTrue)>=1 or count(WalkBarrierTrue)>=1) | you don't have anyone to exercise with. | 480 |
| Text | "BarNoOne" in Barrier1 | they don't have anyone to exercise with. | 490 |
| Text | "BarSelfConscious" in Barrier1 and (count(WalkBarrierVeryTrue)>=1 or count(WalkBarrierTrue)>=1) | you feel self conscious while exercising. | 500 |
| Text | "BarSelfConscious" in Barrier1 | they feel self conscious while exercising. | 510 |
| EndSelect |  |  | 520 |
| Block | not (count(WalkBarrierVeryTrue)>1 or count(WalkBarrierTrue)>=1) |  | 530 |
| Select | 1 |  | 531 |
| Text | OAConWalk>=5 | How does your confidence compare? You told us that your overall confidence level is higher than average. That's great! Without many barriers, and with a large amount of confidence, you're in a great spot as you continue with your walking program. | 540 |
| Text | OAConWalk>=1 and OAConWalk<5 | How does your confidence compare? You told us that your overall confidence is lower than average. That means that your first challenge will be to try to increase your confidence level. To help you do that, let's look at some specific issues that others feel make it hard to walk and solutions to get past them. | 550 |
| Text |  | How does your confidence compare? When you think about your confidence, do you feel that it could be higher? Your first challenge may be to try to increase your confidence level. To help you do that, let's look at some specific issues that others feel make it hard to walk and solutions to get past them. | 551 |
| EndSelect |  |  | 552 |
| EndBlock |  |  | 560 |
| Text | (count(WalkBarrierVeryTrue)>=1 or count(WalkBarrierTrue)>=1) or (not (count(WalkBarrierVeryTrue)>1 or count(WalkBarrierTrue)>=1) and OAConWalk>=5) | Use the tips below to help you find ways to walk when things get in your way. Some of our solutions may even inspire you to come up with your own ideas on how to walk more often. | 570 |
| Comments |  | All Barriers are located in a seperate document. Two barriers will be addressed in this sesssion - one on page 1 and one on page 2. Two more barriers will be addresed in session 5. Each participant will have 4 barriers addressed in total. | 580 |
| Section | Page1Body2 |  | 590 |
| Insert |  | ../../Barriers.html | 610 |
| Section | Page1Body3 |  | 620 |
| Select | 1 |  | 630 |
| Text | count(WalkBarrierVeryTrue)>=2 or count(WalkBarrierTrue)>=2 | To learn solutions to another issue you told us that you face when it comes to walking, please go to the next page. | 640 |
| Text |  | To learn solutions to another issue that many new walkers face when it comes to walking, please go to the next page. | 650 |
| EndSelect |  |  | 660 |
| Section | Page2Header |  | 670 |
| Text |  | **. . . And solutions to keep you walking** | 680 |
| Evaluate |  | Barrier = Barrier2 |  |
| Section | Page2Body |  | 690 |
| Select | 1 |  | 701 |
| Text | count(WalkBarrierVeryTrue)>=2 or count(WalkBarrierTrue)>=2 | You also told us that you don't feel very confident about walking when | 710 |
| Text |  | Another common reason that many people say gets in their way of walking is when | 720 |
| EndSelect |  |  | 721 |
| Select | 1 |  | 730 |
| Text | "BarTime" in Barrier2 and (count(WalkBarrierVeryTrue)>=2 or count(WalkBarrierTrue)>=2) | you don't feel you have the time. | 740 |
| Text | "BarTime" in Barrier2 | they don't feel they have the time. | 750 |
| Text | "BarHealthProb" in Barrier2 | health issues make it difficult. | 760 |
| Text | "BarTired" in Barrier2 and (count(WalkBarrierVeryTrue)>=2 or count(WalkBarrierTrue)>=2) | you feel tired. | 770 |
| Text | "BarTired" in Barrier2 | they feel tired. | 780 |
| Text | "BarEnergy" in Barrier2 and (count(WalkBarrierVeryTrue)>=2 or count(WalkBarrierTrue)>=2) | you don't have the energy to do it. | 800 |
| Text | "BarEnergy" in Barrier2 | they don't have the energy to do it. | 810 |
| Text | "BarHurt" in Barrier2 and (count(WalkBarrierVeryTrue)>=2 or count(WalkBarrierTrue)>=2) | you're afraid of getting hurt. | 820 |
| Text | "BarHurt" in Barrier2 | they're afraid of getting hurt. | 830 |
| Text | "BarEffort" in Barrier2 | it seems to take too much effort. | 850 |
| Text | "BarBadMood" in Barrier2 and (count(WalkBarrierVeryTrue)>=2 or count(WalkBarrierTrue)>=2) | you're in a bad mood. | 860 |
| Text | "BarBadMood" in Barrier2 | they're in a bad mood. | 870 |
| Text | "BarExpensive" in Barrier2 | it's expensive to join a club or gym. | 880 |
| Text | "BarSafePlace" in Barrier2 and (count(WalkBarrierVeryTrue)>=2 or count(WalkBarrierTrue)>=2) | you don't feel you have a safe place to exercise. | 890 |
| Text | "BarSafePlace" in Barrier2 | they don't feel they have a safe place to exercise. | 900 |
| Text | "BarSweat" in Barrier2 and (count(WalkBarrierVeryTrue)>=2 or count(WalkBarrierTrue)>=2) | you might sweat. | 910 |
| Text | "BarSweat" in Barrier2 | they might sweat. | 920 |
| Text | "BarNoOne" in Barrier2 and (count(WalkBarrierVeryTrue)>=2 or count(WalkBarrierTrue)>=2) | you don't have anyone to exercise with. | 930 |
| Text | "BarNoOne" in Barrier2 | they don't have anyone to exercise with. | 940 |
| Text | "BarSelfConscious" in Barrier2 and (count(WalkBarrierVeryTrue)>=2 or count(WalkBarrierTrue)>=2) | you feel self conscious about your looks during exercise. | 950 |
| Text | "BarSelfConscious" in Barrier2 | they feel self conscious about their looks during exercise. | 960 |
| EndSelect |  |  | 970 |
| Text |  | Read below for tips to help with this issue. As you read, think about some additional solutions that you may be able to use to help get past this barrier. | 980 |
| Section | Page2Body2 |  | 990 |
| Insert |  | ../../Barriers.html | 1000 |
| Section | Page2Body3 |  | 1010 |
| Paragraph |  |  | 1020 |
| Block | BarHotWeath>=1 and BarHotWeath<=4 and ShortSeason(BaselineDate, 14)=="Summer" |  | 1030 |
| Text |  | You also told us that hot weather occassionally keeps you from walking. Uncomfortable weather can keep even the most faithful walker inside. But, there are ways to keep cool even if the temperature is high. Explore the tips below to see if you can find a way to walk when it's hot. | 1040 |
| Select | 1 |  | 1050 |
| Text | LtHousework=="Yes" and HeavyHousework=="Yes" | - If it's just too hot to venture outside, **catch up on some housework**. Dusting, vacuuming, washing windows and walking back and forth to the washer and dryer will help increase your daily steps. | 1060 |
| Text | LtHousework=="Yes" and HeavyHousework=="No" | - If it's just too hot to venture outside, **catch up on some housework**. Dusting and washing and putting away dishes can help you increase your daily steps. | 1070 |
| Text | LtHousework=="No" and HeavyHousework=="Yes" | - If it's just too hot to venture outside, **catch up on some housework**. Vacuuming, scrubbing floors and washing windows can help increase your daily steps. | 1080 |
| Text | LtHousework=="No" and HeavyHousework=="No" | - If it's just too hot to venture outside, **catch up on some housework**. Try dusting, vacuuming, or washing windows. All of these will help increase your daily steps. | 1090 |
| Text |  | - If it's just too hot to venture outside, **do some housework**. Dusting and washing and putting away dishes can help you increase your daily steps. | 1100 |
| EndSelect |  |  | 1110 |
| Select | 1 |  | 1120 |
| Text | BarExpensive>4 and containsOne(Employment, ["FullTime", "PartTime"]) and Gender=="Male" | - If you're not already a member, **join a fitness club** near your job. Hit the treadmill or track immediately before or after work. You'll meet other people and avoid the stress of driving in rush-hour traffic. Plus, the cost may be an extra incentive to stick with the program. | 1130 |
| Text | BarExpensive>4 and containsOne(Employment, ["FullTime", "PartTime"]) and Gender=="Female" | - If you're not already a member, **join a fitness club** near your job. Sign up for a group exercise class that meets immediately before or after work. You'll meet other people and avoid the stress of driving in rush-hour traffic. Plus, the cost may be an extra incentive to stick with the program. | 1140 |
| Text | BarExpensive>4 and Gender=="Female" | - **Join a fitness club** near your home. Sign up for a group exercise class that meets once a week. You'll get some great exercise and meet new people. Plus, the cost may be an extra incentive to stick with the program. | 1150 |
| Text | BarExpensive>4 and Gender=="Male" | - **Join a fitness club** near your home. Hit the treadmill or track immediately before or after work. You'll get some great exercise and meet new people. Plus, the cost may be an extra incentive to stick with the program. | 1160 |
| Text |  | - When it's hot outside, it makes sense to move your walks indoors. However, gyms can be expensive. How many gyms does your town have? Look into all of the local gyms in your area and **compare prices**. Perhaps you can join for a half a year or just for the summer. | 1170 |
| EndSelect |  |  | 1180 |
| Text |  | - Drink water to cool down at the beginning and end of your walk. You want to stay **well hydrated** so you don't overheat. | 1190 |
| Text |  | - Walk in the early morning or in the evening around sunset to **avoid the hottest part of the day.** Choose shaded trails or neighborhoods with tree lined sidewalks. | 1200 |
| Paragraph |  |  | 1210 |
| EndBlock |  |  | 1220 |
| Block | BarColdWeath>=1 and BarColdWeath<=4 and ShortSeason(BaselineDate, 14)=="Winter" |  | 1230 |
| Text |  | You also told us that cold weather keeps you from walking. Cold weather may seem like a great excuse to skip your walk for the day. But you don't have to make excuses! It is possible to stay warm even if the temperatures are low. | 1240 |
| Text | BarExpensive>4 | - **Join a local gym,** even if it's just for the winter months. Choose one that's convenient and geared toward your fitness level. Ask the staff to show you how to use the equipment. | 1250 |
| Text | ChildInHome=="No" and not SF36LowIntAct in ("SomeLimit," "SigLimit") | - Is it snowing? **Snow can be fun**! Be a kid again. Throw snowballs and make snow angels or snowmen on your walk. | 1260 |
| Text | ChildInHome=="Yes" and not SF36LowIntAct in ("SomeLimit," "SigLimit") | - Is it snowing? **With kids in the home**, snow can add a fun element to your walks. Join in on their fun. Throw snowballs and make snow angels or snowmen during your walk. | 1270 |
| Text |  | - Make sure your shoes have **good traction**. You don't want to slip or fall on any icy patches. If the sidewalk does look icy, walk in the snowy grass. | 1280 |
| Text |  | - Find a **warm place** to walk indoors. Walk at the mall, at an indoor track, or on a treadmill. | 1290 |
| Text |  | - **Run some errands**. Head to a department or grocery store and walk the aisles. | 1300 |
| Paragraph |  |  | 1310 |
| EndBlock |  |  | 1320 |
| Text | not isEmpty(AddressPref) | $AddressPref, as we come to the end of this session, we hope the solutions we provided will help as you work toward your walking goal. Which, if any, of the ideas are you willing to try this week? | 1330 |
| Text | isEmpty(AddressPref) | As we come to the end of this session, we hope the solutions we provided will help as you work toward your walking goal. Which, if any, of the ideas are you willing to try this week? | 1331 |
| Paragraph |  |  | 1340 |
| Text |  | **What to expect next** | 1350 |
| Paragraph |  |  | 1360 |
| Text |  | In one week, you will receive an email message from **Stepping Up To Health** letting you know that your third personalized Web session is available. The session will focus on your own, personal motivations. Until then, continue to **upload your pedometer**, review your walking charts, and visit often to read your **Daily Tip**. | 1370 |
