# Supplementary material for: An Online Community Improves Adherence in an Internet-Mediated Walking Program. Part 1: Results of a Randomized Controlled Trial
Source: J Med Internet Res. 2010 Dec 17;12(4):e71. doi: 10.2196/jmir.1338 (PMC3056526; doi:10.2196/jmir.1338)
Supplement: Supplementary file 11 [file jmir_v12i4e71_app11.html]

WG3.html


SUH Session 3, pages 1 and 2 - Last Revision August 21, 2006

|  |  |  |  |
| --- | --- | --- | --- |
| **Command** | **Logic** | **Message** | **Row** |
| Comment | WG3.html | Goal of Motivation Session (3): Discuss motivation levels. Internal vs. negative introject vs. external drive. Moving from extrinsic to intrinsic motivations. Their desires/reasons for participating. | 10 |
| Section | Page1Header |  | 20 |
| Text |  | **For all the right reasons** | 21 |
| Section | Page1Body |  | 30 |
| Text | not isEmpty(AddressPref) | $AddressPref, welcome | 40 |
| Text | isEmpty(AddressPref) | Welcome | 50 |
| Text |  | back to your third of six **Stepping Up To Health** sessions. You may remember that back in the first newsletter, we discussed exercise safety and in the second newsletter, we talked about ways to help overcome some of the challenges that keep you from reaching your daily step goal. In this session, we'd like to share some ideas that can keep you focused on the path to walking. This session is about motivating and encouraging you to increase your daily steps. | 51 |
| Paragraph |  |  | 60 |
| Text |  | Let's begin by looking at your current level of overall motivation. | 70 |
| Block | OAMotWalk>=1 |  | 80 |
| Text |  | In your survey, you told us that you aren't walking much during the day. You rated your motivation to walk more often as $int(OAMotWalk) out of 10. | 90 |
| Text | OAMotWalk>=1 and OAMotWalk<=3 | **So, you aren't feeling very motivated to walk each day**. | 110 |
| Text | OAMotWalk>3 and OAMotWalk<=6 | **So, you are feeling fairly motivated to walk each day**. | 120 |
| Text | OAMotWalk>6 and OAMotWalk<=8 | **So, you are feeling very motivated to start walking each day**. | 130 |
| Text | OAMotWalk>8 | **So, you are feeling exceptionally motivated to start walking each day**. | 140 |
| Text | OAMotWalk>=1 and OAMotWalk<=6 | What would it take to get you to go from $int(OAMotWalk) to a higher number such as $plus(OAMotWalk, 2) or $plus(OAMotWalk, 3)? | 150 |
| Text | OAMotWalk>6 and OAMotWalk<9 | What would it take to get you to go from $int(OAMotWalk) to a higher number such as $plus(OAMotWalk, 1) or $plus(OAMotWalk, 2)? | 160 |
| Text | OAMotWalk>=9 | Why was your motivation at $int(OAMotWalk) and not a lower number? In other words, what makes it so important to you? Take a few minutes to write down your thoughts.  - What can you do to keep your motivation strong? - What potential challenges lie ahead that could derail your plans to walk more? - How can you focus on the good things that come from exercise? | 170 |
| EndBlock |  |  | 190 |
| Text | isEmpty(OAMotWalk) | On a scale from 1-10, where 1 is not at all motivated and 10 extremely motivated, how would you rate your **motivation to walk right now**? | 200 |
| Paragraph |  |  | 210 |
| Text | IntrinsicMotive=="High" or ExtrinsicMotive=="High" or IntrojectMotive=="High" | Now, let's look a little more closely at what specifically motivates you. | 220 |
| Text | IntrinsicMotive=="High" | Based on what you shared with us, it sounds like you have some meaningful reasons for wanting to walk more often. You've told us that walking is: | 230 |
| Block | IntrinsicMotive=="High" and not ExtrinsicMotive=="High" and not IntrojectMotive=="High" |  | 240 |
| Select | 3 |  | 250 |
| Text | "IMPreventFuture" in Top3InternalMot | - important in preventing future health problems. | 260 |
| Text | "IMResponsible" in Top3InternalMot | - something you can do to take responsibility for your own health. | 270 |
| Text | "IMPhysHealth" in Top3InternalMot | - a key part of improving your physical health. | 280 |
| Text | "IMEnjoy" in Top3InternalMot | - something you enjoy. | 290 |
| Text | "IMBelieve" in Top3InternalMot | - something you believe is a good thing. | 300 |
| Text | "IMManageWeight" in Top3InternalMot | - a way to manage your weight. | 310 |
| Text | "IMDailyAct" in Top3InternalMot | - something that can help improve your ability to do daily activities. | 320 |
| Text | "IMIncreaseEnergy" in Top3InternalMot | - a way to increase energy levels. | 330 |
| Text | "IMAccomplishment" in Top3InternalMot | - important to having a sense of accomplishment. | 340 |
| EndSelect |  |  | 350 |
| EndBlock |  |  | 360 |
| Block | IntrinsicMotive=="High" and (ExtrinsicMotive=="High" or IntrojectMotive=="High") |  | 370 |
| Select | 2 |  | 380 |
| Text | "IMPreventFuture" in Top2InternalMot | - important in preventing future health problems. | 390 |
| Text | "IMResponsible" in Top2InternalMot | - something you can do to take responsibility for your own health. | 400 |
| Text | "IMPhysHealth" in Top2InternalMot | - a key part of improving your physical health. | 410 |
| Text | "IMEnjoy" in Top2InternalMot | - something you enjoy. | 420 |
| Text | "IMBelieve" in Top2InternalMot | - something you believe is a good thing. | 430 |
| Text | "IMManageWeight" in Top2InternalMot | - a way to manage your weight. | 440 |
| Text | "IMDailyAct" in Top2InternalMot | - something that can help improve your ability to do daily activities. | 450 |
| Text | "IMIncreaseEnergy" in Top2InternalMot | - a way to increase energy levels. | 460 |
| Text | "IMAccomplishment" in Top2InternalMot | - important to having a sense of accomplishment. | 470 |
| EndSelect |  |  | 480 |
| EndBlock |  |  | 490 |
| Text | IntrojectMotive=="High" and not IntrinsicMotive=="High" | Based on your survey responses, it seems you are often your own toughest critic when it comes to exercise. You've told us that you might feel | 500 |
| Text | IntrojectMotive=="High" and IntrinsicMotive=="High" | There are also times when you are your own toughest critic when it comes to exercise. You've told us that you might feel | 510 |
| Block | IntrojectMotive=="High" and not ExtrinsicMotive=="High" and not IntrinsicMotive=="High" |  | 520 |
| Select | 2 |  | 530 |
| Text | "NMGuilt" in Top3IntrojectMot | - guilty or ashamed | 540 |
| Text | "NMBadAboutSelf" in Top3IntrojectMot | - bad about yourself | 550 |
| Text | "NMRegret" in Top3IntrojectMot | - regretful | 560 |
| EndSelect |  |  | 570 |
| EndBlock |  |  | 580 |
| Block | IntrojectMotive=="High" and (ExtrinsicMotive=="High" or IntrinsicMotive=="High") |  | 590 |
| Select | 1 |  | 600 |
| Text | "NMGuilt" in Top2IntrojectMot | guilty or ashamed | 610 |
| Text | "NMBadAboutSelf" in Top2IntrojectMot | bad about yourself | 620 |
| Text | "NMRegret" in Top2IntrojectMot | regretful | 630 |
| EndSelect |  |  | 640 |
| EndBlock |  |  | 650 |
| Text | IntrojectMotive=="High" | if you did not walk. | 660 |
| Text | ExtrinsicMotive=="High" and not IntrojectMotive=="High" and not IntrinsicMotive=="High" | You've told us that you want to walk as part of a healthy lifestyle: | 670 |
| Text | ExtrinsicMotive=="High" and (IntrojectMotive=="High" or IntrinsicMotive=="High") | You've also told us that you want to walk as part of a healthy lifestyle: | 680 |
| Block | ExtrinsicMotive=="High" and not IntrinsicMotive=="High" and not IntrojectMotive=="High" |  | 690 |
| Select | 3 |  | 700 |
| Text | "EMOtherWant" in Top3ExtrinsicMotive | - because other people want you to. | 710 |
| Text | "EMGdExFam" in Top3ExtrinsicMotive | - so you can set a good example for your family. | 720 |
| Text | "EMDoctor" in Top3ExtrinsicMotive | - because your doctor told you to exercise more. | 730 |
| Text | "EMGdExCom" in Top3ExtrinsicMotive | - so you can set a good example for your community. | 740 |
| Text | "EMUpsetOthers" in Top3ExtrinsicMotive | - because others would be upset with you if you didn't. | 750 |
| Text | "EMOthersDown" in Top3ExtrinsicMotive | - so you won't let others down. | 760 |
| Text | "EMOthersSee" in Top3ExtrinsicMotive | - because you want others to see that you can. | 770 |
| Text | "EMTold" in Top3ExtrinsicMotive | - because you feel it's easier to do what you are told. | 780 |
| EndSelect |  |  | 790 |
| EndBlock |  |  | 800 |
| Block | ExtrinsicMotive=="High" and (IntrinsicMotive=="High" or IntrojectMotive=="High") |  | 810 |
| Select | 2 |  | 820 |
| Text | "EMOtherWant" in Top2ExtrinsicMotive | - because other people want you to. | 830 |
| Text | "EMGdExFam" in Top2ExtrinsicMotive | - so you can set a good example for your family. | 840 |
| Text | "EMDoctor" in Top2ExtrinsicMotive | - because your doctor told you to exercise more. | 850 |
| Text | "EMGdExCom" in Top2ExtrinsicMotive | - so you can set a good example for your community. | 860 |
| Text | "EMUpsetOthers" in Top2ExtrinsicMotive | - because others would be upset with you if you didn't. | 870 |
| Text | "EMOthersDown" in Top2ExtrinsicMotive | - so you won't let others down. | 880 |
| Text | "EMOthersSee" in Top2ExtrinsicMotive | - because you want others to see that you can. | 890 |
| Text | "EMTold" in Top2ExtrinsicMotive | - because you feel it's easier to do what you are told. | 900 |
| EndSelect |  |  | 910 |
| EndBlock |  |  | 920 |
| Select | 1 |  | 930 |
| Text | IntrinsicMotive=="High" and not ExtrinsicMotive=="High" and not IntrojectMotive=="High" | Being motivated by reasons like the ones you shared with us can help put you on the path to success. Many people find that lifestyle changes, such as walking more often, are longer lasting when they have meaningful reasons like the ones listed above. | 940 |
| Text | IntrojectMotive=="High" and not ExtrinsicMotive=="High" and not IntrinsicMotive=="High" | Reasons like the ones listed above can get you started on the road to a healthier lifestyle. Over time though, many people find that lifestyle changes, such as walking more often, will be longer lasting if they find more meaningful reasons for doing them. For example, empowering motivators might include the desire to improve your physical health or to gain a sense of accomplishment. | 950 |
| Text | ExtrinsicMotive=="High" and not IntrinsicMotive=="High" and not IntrojectMotive=="High" | It's clear that you value strong relationships with people who care about your health. Over time though, many people find that lifestyle changes, such as walking more often, will be longer lasting if they find more personal reasons for doing them. For example, empowering motivators might include the desire to improve your physical health or to gain a sense of accomplishment. | 960 |
| Text | not ExtrinsicMotive=="High" and IntrinsicMotive=="High" and IntrojectMotive=="High" | Reasons like these can get you started on the road to a healthier lifestyle. But many people find that lifestyle changes, such as walking more often, will be longer lasting if they focus on more meaningful reasons for doing them. For you, that would mean focusing more on the two bullets we listed above and not your feelings of guilt. | 970 |
| Text | ExtrinsicMotive=="High" and IntrinsicMotive=="High" and not IntrojectMotive=="High" | It's clear that you have strong relationships with people who care about your health. But many people find that lifestyle changes, such as walking more often, will be longer lasting if they focus on more personal reasons. For you, that would mean focusing more on the first two bullets we listed above. | 980 |
| Text | ExtrinsicMotive=="High" and IntrojectMotive=="High" and not IntrinsicMotive=="High" | It's clear that you have strong relationships with people who care about your health. Reasons like these can get you started on the road to being healthier. But many people find that lifestyle changes, such as walking more often, will be longer lasting if they find more meaningful reasons for doing them. For example, empowering motivators might include the desire to improve your physical health or to gain a sense of accomplishment. | 990 |
| Text | ExtrinsicMotive=="High" and IntrojectMotive=="High" and IntrinsicMotive=="High" | It's clear that you have strong relationships with people who care about your health. Reasons like these can get you started on the road to better eating. But many people find that lifestyle changes, such as walking more often, will be longer lasting if they focus on more personal reasons. For you, that would mean focusing more on the first two bullets we listed above. | 1000 |
| Text |  | Sometimes it's hard to get motivated about walking. But, have you considered some of the reasons other people have that motivate them to walk more? Regular walking can be:  - important for being as healthy as possible. - important for gaining a sense of accomplishment. - a key part of taking responsibility for your health.  Many people find that lifestyle changes, such as walking more often, are longer lasting when they have meaningful reasons like the ones above associated with the change they want to make. | 1010 |
| EndSelect |  |  | 1020 |
| Paragraph |  |  | 1030 |
| Text |  | To help you think more about your reasons to walk, we encourage you to do the exercise on the next page. It can help you think about the personal values and roles that are important to you. | 1040 |
| Comment |  | Comment: All bullets in this section are going to need squares instead of circles. We want the page to look like they could check the boxes if they print it off. | 1050 |
| Section | Page2Header |  | 1060 |
| Text |  | **What's important to you?** | 1070 |
| Section | Page2Body |  |  |
| Select | 1 |  | 1080 |
| Text | IntrinsicMotive=="High" | Since your motivation comes mostly from within yourself, the exercise below can only strengthen your focus on walking. | 1090 |
| Text | IntrinsicMotive=="Med" | Since part of your motivation comes from within yourself, the exercise below can only strengthen your internal reasons for wanting to walk. | 1100 |
| Text | IntrinsicMotive=="Low" | You aren't very motivated by internal reasons to walk. Try the exercise below. It can help you see how your values could be impacted by the choices you make. | 1110 |
| Text |  | What's really motivating your internal desire to walk? Try the exercise below. It can help you see how your values could be impacted by the choices you make. | 1120 |
| EndSelect |  |  | 1130 |
| Paragraph |  |  | 1140 |
| Text |  | Values are filters we use to evaluate ourselves and the world around us. These values sort our perceptions into good and bad, worthwhile and worthless, and so on. Look at the list below. Reflect on the three or four items you value most in life. Or, print off this sheet and fill it out. | 1150 |
| Paragraph |  |  | 1160 |
| Text |  | **I value being:** | 1170 |
| Paragraph |  |  | 1180 |
| Comment |  | Similar to Menu and PQ. - Except it won't be interactive. They can print off and fill in if they'd like. Is it possible for there to be two rows of bullets? The page is super long right now because of the long list. | 1190 |
| List |  |  |  |
| ListItem | MaritalStatus=="Married" and Gender=="Female" | a good wife | 1200 |
| ListItem | MaritalStatus=="Married" and Gender=="Male" | a good husband | 1210 |
| ListItem | MaritalStatus=="Partner" | a good partner | 1220 |
| ListItem | HomeStatus=="No" | respected at home | 1230 |
| Comment |  | The rest of the bullets are for everyone - no need to program logic for them. | 1240 |
| ListItem |  | a good community member |  |
| ListItem |  | strong |  |
| ListItem |  | on top of things |  |
| ListItem |  | competent |  |
| ListItem |  | spiritual |  |
| ListItem |  | successful |  |
| ListItem |  | healthy |  |
| ListItem |  | attractive |  |
| ListItem |  | disciplined |  |
| ListItem |  | responsible |  |
| ListItem |  | in control |  |
| ListItem |  | honest |  |
| ListItem |  | energetic |  |
| ListItem |  | considerate |  |
| ListItem |  | youthful |  |
| ListItem |  | independent |  |
| ListItem |  | creative |  |
| ListItem |  | dependable |  |
| ListItem |  | caring |  |
| ListItem |  | helpful |  |
| ListItem |  | accepting |  |
| EndList |  |  |  |
| Comment |  | The following should have a "worksheet" feel. Please add space after each question for someone to be able to print off the sheet and write in their answers. | 1260 |
| Paragraph |  |  | 1270 |
| Text |  | Think about the answers that you would check. These things are most important to you. Which, if any, of these roles could motivate you to increase your daily steps?  \_\_\_\_\_\_\_\_\_\_\_\_\_\_\_\_\_\_\_\_\_\_\_\_\_\_\_\_\_\_\_\_\_\_\_\_\_\_\_\_\_\_\_\_\_\_\_\_\_\_\_\_\_\_\_\_\_\_\_\_\_\_  \_\_\_\_\_\_\_\_\_\_\_\_\_\_\_\_\_\_\_\_\_\_\_\_\_\_\_\_\_\_\_\_\_\_\_\_\_\_\_\_\_\_\_\_\_\_\_\_\_\_\_\_\_\_\_\_\_\_\_\_\_\_  \_\_\_\_\_\_\_\_\_\_\_\_\_\_\_\_\_\_\_\_\_\_\_\_\_\_\_\_\_\_\_\_\_\_\_\_\_\_\_\_\_\_\_\_\_\_\_\_\_\_\_\_\_\_\_\_\_\_\_\_\_\_ | 1280 |
| Paragraph |  |  | 1290 |
| Text |  | How do you feel that increasing your daily steps positively affects your ability to achieve these roles or live out any of these values? For example, "*When I walk during the day, I find that I have energy and can stay on top of all the things I need to do throughout the day.*"  \_\_\_\_\_\_\_\_\_\_\_\_\_\_\_\_\_\_\_\_\_\_\_\_\_\_\_\_\_\_\_\_\_\_\_\_\_\_\_\_\_\_\_\_\_\_\_\_\_\_\_\_\_\_\_\_\_\_\_\_\_\_  \_\_\_\_\_\_\_\_\_\_\_\_\_\_\_\_\_\_\_\_\_\_\_\_\_\_\_\_\_\_\_\_\_\_\_\_\_\_\_\_\_\_\_\_\_\_\_\_\_\_\_\_\_\_\_\_\_\_\_\_\_\_  \_\_\_\_\_\_\_\_\_\_\_\_\_\_\_\_\_\_\_\_\_\_\_\_\_\_\_\_\_\_\_\_\_\_\_\_\_\_\_\_\_\_\_\_\_\_\_\_\_\_\_\_\_\_\_\_\_\_\_\_\_\_ | 1300 |
| Paragraph |  |  | 1310 |
| Text |  | Let your own, personal motivations be your guide to a healthier you. | 1320 |
| Paragraph |  |  | 1330 |
| Text |  | **What to expect next** | 1350 |
| Paragraph |  |  | 1360 |
| Text |  | Next week, you will receive an email message from **Stepping Up To Health** letting you know that your fourth personalized Web session is available. The session will focus on the many benefits of regular walking and how to reach out to others for help. | 1370 |
| Paragraph |  |  | 1380 |
| Text |  | Until then, continue to **upload your pedometer**, review your walking charts, and visit often to read your **Daily Tip**. | 1390 |
