# Supplementary material for: An Online Community Improves Adherence in an Internet-Mediated Walking Program. Part 1: Results of a Randomized Controlled Trial
Source: J Med Internet Res. 2010 Dec 17;12(4):e71. doi: 10.2196/jmir.1338 (PMC3056526; doi:10.2196/jmir.1338)
Supplement: Supplementary file 12 [file jmir_v12i4e71_app12.html]

WG4.html


SUH - Session 4, pages 1 and 2 - Last Revision August 10, 2006

|  |  |  |  |
| --- | --- | --- | --- |
| **Command** | **Logic** | **Message** | **Row** |
| Section | Page1Header |  | 10 |
| Text |  | **Your support network** | 20 |
| Section | Page1Body |  | 30 |
| Text | not isEmpty(AddressPref) | $AddressPref, welcome | 40 |
| Text | isEmpty(AddressPref) | Welcome | 50 |
| Text |  | back to your fourth personalized web guide from **Stepping Up To Health**. This session will focus on the support that you may receive from your friends and family. We'll also review the many personal benefits that you may gain from walking. | 60 |
| Paragraph |  |  | 70 |
| Block | CurrentSupport=="No" |  | 80 |
| Text |  | In your survey, you told us that your friends and family don't quite provide the support you feel you might need to be more active. **Making lifestyle changes can be easier though, when you have the support of important people in your life.** Support can come in different forms, like through words of encouragement or the willingness of others to join you on walks. If you put in a little effort up front, you just might find someone who can be there for you. | 90 |
| Paragraph |  |  | 91 |
| Text |  | *Try this*: Pick one person in your life that you feel comfortable with. Tell them why you want to be more active and what your plan of action is. Ask them if they can help. Give them ideas on how they can help, like the ones below. Do you feel that this is something you can do this week? | 92 |
| Paragraph |  |  | 93 |
| Text |  | Below, we have a list of people who may be important in your life. Which, if any, of the family and friends listed do you think could encourage you to get in more steps? Also, try to think of some ways they could help you walk more. | 94 |
| EndBlock |  |  | 110 |
| Block | CurrentSupport=="Yes" or isEmpty(CurrentSupport) |  | 120 |
| Text |  | **It's easier to make lifestyle changes when you have the support of important people in your life.** We're connected to the people around us through bonds of love, friendship, and community. Support can come in many different forms, through words of encouragement from friends and family, to group walks where everyone increases their steps together. | 130 |
| Paragraph |  |  | 140 |
| Select | 1 |  |  |
| Text | containsOne (WhoSupport, ["Spouse", "Sister", "Brother", "Child", "Mom", "Dad", "Friend", "Coworker"]) | You shared with us that **$MainSupport** is supportive of your decision to get more physical activity and will help you reach your goals. In what ways do they help? Below, we have a list of additional people who may be important in your life. Try to think of some additional ways they can help you walk more. | 150 |
| Text |  | Which, if any, of the family and friends listed below do you think could encourage you to get in more steps? Also, try to think of some ways they could help you walk more. | 170 |
| EndSelect |  |  |  |
| Paragraph |  |  |  |
| EndBlock |  |  | 180 |
| Paragraph |  |  | 190 |
| Text |  | **Potential supporters:** | 200 |
| Paragraph |  |  | 210 |
| Comment |  | Question for Mike - Is it possible for the bullets under Potential Supports to be next to the How They Can Help bullets? I'd rather have them side by side vs. on top of each other. | 220 |
| List |  |  |  |
| ListItem | MaritalStatus=="Married" and Gender=="Female" | husband | 230 |
| ListItem | MaritalStatus=="Married" and Gender=="Male" | wife | 240 |
| ListItem | MaritalStatus=="Partner" | partner | 250 |
| ListItem |  | children |  |
| ListItem |  | grandchildren |  |
| ListItem |  | parent |  |
| ListItem |  | sister/brother |  |
| ListItem |  | aunt/uncle |  |
| ListItem |  | relatives |  |
| ListItem |  | friends |  |
| ListItem |  | co-workers |  |
| ListItem |  | neighbors |  |
| ListItem |  | community groups |  |
| ListItem |  | support groups |  |
| EndList |  |  |  |
| Paragraph |  |  | 270 |
| Text |  | **How they can help:** | 280 |
| Paragraph |  |  | 290 |
| Text |  | - Provide encouraging words of support during weekly phone calls. - Join in on walks and events to increase your daily steps. - Remind you of why you want to walk more when you find yourself skipping walks. - Wear a pedometer as well and compare steps at the end of each day. Make it a friendly competition to see who walks more! | 300 |
| Paragraph |  |  | 310 |
| Text | CurrentSupport=="Yes" or isEmpty(CurrentSupport) | Do you think you could approach any of these people for help? Can you think of any more ways your support network can help you reach your goals? | 320 |
| Paragraph |  |  | 330 |
| Text |  | It would be wonderful if we could all anticipate exactly when and what type of help we need but, unfortunately, it usually doesn't work out that way. It takes a strong person to ask for help. But the response can be worth it. | 340 |
| Comment |  | Goal of Page 2 of Motivation Session #1: Highlight health benefits of eating fruit and vegetables. | 350 |
| Section | Page2Header |  | 360 |
| Text |  | **Walking and your health** | 370 |
| Section | Page2Body |  | 380 |
| Text |  | Walking is an activity that many Americans engage in on a daily basis. It is functional, social, fun and has numerous health benefits. | 390 |
| Select | 1 |  | 400 |
| Text | len(HealthHistory)==1 or len(HealthHistory)==2 | From what you shared with us, we know that you have been diagnosed with $englishList(HealthTreat, 1,2). Did you know that regular walking can help you manage your health? | 410 |
| Text | len(HealthHistory)>2 | From what you shared with us, we know that you have been diagnosed with a few health conditions, including $englishList(HealthTreat, 1,2). Did you know that regular walking can help you manage your health? | 420 |
| Text | len(HealthHistory)==0 and len(FamilyHealthHist)==0 | **You don't have any major health conditions that impact many Americans. You also don't have a family history of major illnesses**. Regular walking can help you stay healthy and lower your chances of developing serious conditions. | 430 |
| Text | len(HealthHistory)==0 | Based on the health conditions we asked about in our survey, **you don't have any major health conditions that impact many Americans**. Regular walking can help you stay healthy and greatly lower your chances of developing serious conditions. | 440 |
| Text |  | **It's true**, regular walking can help you be healthy and lower your chances of developing serious health conditions that impact many Americans today. | 450 |
| EndSelect |  |  | 460 |
| Paragraph |  |  | 461 |
| Text |  | In fact, scientists are constantly learning more about the many ways walking can contribute to your overall well-being. Walking requires no prescription, the risk of side effects is very low, and the list of benefits continues to grow. | 470 |
| Comment |  | Source for Kidney facts: http://www.kidney.org/kidneydisease/ckd/index.cfm and http://www.kidney.org/atoz/pdf/stayfit.pdf (row 590) | 471 |
| Select | 4 |  | 480 |
| Text | CADSev in ("High", "Low") | - Over time, regular walking can help **strengthen your heart** and prevent future health problems. | 490 |
| Text | "Stroke" in HealthHistory | - Along with other healthy lifestyle changes, regular walking can lower your risk of having another **stroke**. | 500 |
| Text | DIABSev in ("High", "Low") | - Walking can be an important part of managing your **diabetes**. Walking is a great way to regulate your blood sugar levels. | 510 |
| Text | "Asthma" in HealthHistory | - Walking can improve your **heart and lung fitness**, which can help prevent asthmatic symptoms, such as wheezing or difficulty breathing, in the future. | 520 |
| Text | Gender=="Female" and Age>=50 | - Walking is a great form of weight-bearing exercise that can help **strengthen your bones**, increase bone density and prevent you from falling and getting hurt. | 530 |
| Text | "MdDisorder" in HealthHistory | - Research has shown that you can decrease mental stress, decrease depression, and **improve your overall mood** through regular walking. | 540 |
| Text | "IGT" in HealthHistory and not DIABSev in ("High", "Low") | - **Pre-diabetes** (Impaired Glucose Tolerance) does not have to become diabetes. Walking can help you lose excess weight, which helps your body use glucose more effectively. | 550 |
| Text | "ChronicPain" in HealthHistory | - When you're in pain, exercise is probably the last thing on your mind. However, walking can help ease **chronic pain**. Walking will help you build strength, increase your flexibility and prompt your body to release endorphins -- all which can reduce your pain. | 560 |
| Text | "HBP" in HealthHistory | - Walking can be a great way for you to **control your blood pressure**. Walking strengthens the heart so it can pump more blood with less effort and with less pressure on the arteries. | 570 |
| Text | "HiChol" in HealthHistory | - You told us you have **high cholesterol**. Walking is a great way to increase your HDL (good) cholesterol and lower your LDL (bad) cholesterol. | 580 |
| Text | "Kidney" in HealthHistory | - 1 in 9 adults have **kidney disease** and those people, like you, greatly benefit from regular exercise. Walking will help control your blood pressure, lower your level of blood fats, and help control your body weight, which will help reduce the strain on your kidneys. | 590 |
|  |  | - Walking can slow aging, burn body fat, reduce stress, help control your appetite, raise your metobolism, and increase your energy. |  |
| Text |  | - On average, every minute of walking can **extend your life** by 1 ½ to 2 minutes. | 600 |
| Text |  | - Walking an extra 20 minutes each day can burn off 7 pounds of body fat per year. | 610 |
| Text |  | - Walking has numerous other benefits - including improving circulation, self-esteem, and your posture. | 620 |
| EndSelect |  |  | 630 |
| Paragraph |  |  | 631 |
| Text |  | Walking can also help you stay as healthy as possible and prevent health problems in the future. | 632 |
| Select | 2 |  | 640 |
| Text | not CADSev in ("High", "Low") and Gender=="Female" | - Heart disease is the **#1 killer of women** in the United States. Regular walking can help lower your risk. | 650 |
| Text | not CADSev in ("High", "Low") and Gender=="Male" | - Heart disease is the **#1 killer of men** in the United States. Regular walking can help lower your risk. | 660 |
| Text | not DIABSev in ("High", "Low") and "IGT" not in HealthHistory | - Regular walking is a great way to **prevent diabetes**. | 670 |
| Text | BarTired<=3 | - Walking is a great way to **reduce the feeling of being tired**. When you get into a walking routine, you'll begin to notice that you sleep better at night and are less tired during the day. | 680 |
| Text | "BreastCancer" not in HealthHistory and "ColonCancer" not in HealthHistory and "ProstateCancer" not in HealthHistory and "LungCancer" not in HealthHistory and "OtherCancer" not in HealthHistory | - **Cancer** is the second leading cause of death in the U.S. The most common cancers are lung, breast, prostate, and colon. Physical activity is proven to have cancer-protective effects. | 690 |
| Text | Age>=50 | - **Walkers live longer**. Walking can help prevent hip fractures, keep you flexible, and keep your mind clearer into old age. | 700 |
| Text | Age>1 and Age<50 | - **Walkers live longer.** Having a regular walking schedule can actually add years to your life. | 701 |
| Text |  | - Regular exercise has shown to reduce the number of episodes of the common cold each year. That means spending less time sick in bed! | 710 |
| EndSelect |  |  | 720 |
| Select | 1 |  | 730 |
| Text | BMI>=30 and TryingLoseWt=="Yes" | - As someone who is **overweight**, you are at an increased risk of having health problems. Walking can help you prevent heart disease by strengthening your heart muscle. And since you told us that you're trying to lose weight, walking can be a good way to burn off some extra calories. | 740 |
| Text | BMI>=30 and "Diabetes" not in HealthHistory | - As someone who is **overweight**, you are at an increased risk of having health problems. Walking can help you prevent heart disease by strengthening your heart muscle. Walking has also been proven to reduce the risk of diabetes due to excess weight. | 750 |
| Text | BMI>=30 and ("Stroke" not in HealthHistory and"ChronicPain" not in HealthHistory and "SleepProb" not in HealthHistory and "Osteo" not in HealthHistory) and not (CADSev== "High" or CADSev== "Low" ) | - Walking is a great way to **manage your weight**. Combined with healthy eating, physical activity is key to long-lasting weight control. Keeping your weight within healthy limits can lower your risk for heart disease, stroke, sleep apnea, and osteoarthritis. | 760 |
| Text |  | - Walking is a good way to **manage your weight**. Combined with healthy eating, physical activity is key to long-lasting weight control. | 770 |
| EndSelect |  |  | 771 |
| Select | 1 |  | 780 |
| Text | ("Angina" not in HealthHistory and "HeartFail" not in HealthHistory and "HeartAttack" not in HealthHistory and "CAD" not in HealthHistory) and "CVD" in FamilyHealthHist | - High cholesterol and blood pressure play a major role in the development of heart disease. Regular walking can help control these and lower your risk of heart disease. This is important to keep in mind with your **family history** of heart disease. | 790 |
| Text | "Diabetes" in FamilyHealthHist and "Diabetes" not in HealthHistory | - Even though someone in **your family** has diabetes, you can lower your risk for it by regularly walking. Exercise can help keep your weight down (being overweight is one of the biggest risk factors for developing adult-onset diabetes) and control blood sugar levels. | 800 |
| Text | "HiChol" in FamilyHealthHist and "HiChol" not in HealthHistory | - **High cholesterol** is part of your family's health history. If your family has a regular pattern of early heart disease (before age 55 in a man and before age 65 in a woman), it may mean that people in your family have an inherited tendency for high cholesterol levels. Even with this risk, you can control your overall risk of high cholesterol by increasing your daily steps. | 810 |
| Text | "Arthritis" in FamilyHealthHist and "Arthritis" not in HealthHistory | - Research has shown that walking can also help prevent future health problems, **such as arthritis.** | 840 |
| Text | "Osteo" in FamilyHealthHist and "Osteo" not in HealthHistory | - Research has shown that walking can also help prevent future health problems, **such as osteoporosis.** | 850 |
| Text | "HBP" in FamilyHealthHist and "HBP" not in HealthHistory | - Research has shown that walking can also help prevent future health problems, **such as high blood pressure.** | 860 |
| Text | Age>=50 | - Research has shown that walking can also help prevent future health problems, **including those associated with getting older**, such as arthritis and osteoporosis. | 870 |
| EndSelect |  |  | 880 |
| Text | Age>=50 | - People your age typically don't exercise that often. Exercise can improve your ability to be **independent**. Active people over the age of 50 have been found to be 20% to 50% less likely to lose their independence over time. | 890 |
| Paragraph |  |  | 891 |
| Text |  | The benefits of regular walking are huge. You get all of these great benefits, without any prescriptions or trips to the doctor. You can get them simply by lacing up your shoes and heading out the door! | 892 |
| Paragraph |  |  | 893 |
| Text |  | **What to expect next** | 910 |
| Paragraph |  |  | 911 |
| Text | not isEmpty(AddressPref) | $AddressPref, as we come | 920 |
| Text | isEmpty(AddressPref) | As we come | 930 |
| Text |  | to the end of your fourth session, we hope you have learned a little more about how to find the support that is right for you as well as all of the benefits that walking has to offer. While you are here, don't forget to upload your pedometer and check your daily tip! | 940 |
| Paragraph |  |  | 950 |
| Text |  | One week from now, you will receive your fifth personalized session. This session will continue to discuss more of the barriers that you may be facing when it comes to walking. Watch for an email message letting you know when it's available. | 960 |
