# Supplementary material for: An Online Community Improves Adherence in an Internet-Mediated Walking Program. Part 1: Results of a Randomized Controlled Trial
Source: J Med Internet Res. 2010 Dec 17;12(4):e71. doi: 10.2196/jmir.1338 (PMC3056526; doi:10.2196/jmir.1338)
Supplement: Supplementary file 13 [file jmir_v12i4e71_app13.html]

Web Guide 5


SUH - Session 5, pages 1 and 2 of the session. Last Revision August 17, 2006

|  |  |  |  |
| --- | --- | --- | --- |
| **Command** | **Logic** | **Message** | **Row** |
| Comment |  | General comments: All Barriers. One barrier will be inserted into session 5, page 1 and 2. Each participant will get 4 barriers. | 10 |
| Section | Page1Header |  | 20 |
| Text |  | **What might make changing hard - part 2** | 30 |
| Evaluate |  | Barrier = Barrier3 | 35 |
| Section | Page1Body |  | 40 |
| Text |  | When you first enrolled in **Stepping Up To Health**, | 50 |
| Select | 1 |  | 60 |
| Text | count(WalkBarrierVeryTrue)>=2 | you had **some definite concerns** about being able to | 70 |
| Text | count(WalkBarrierVeryTrue)==1 or count(WalkBarrierTrue)>=2 | you had **some serious concerns** about being able to | 80 |
| Text | count(WalkBarrierTrue)>=1 | you had **some slight concerns** about being able to | 90 |
| Text |  | you didn't mention **any significant barriers** keeping you from being able to | 100 |
| EndSelect |  |  | 110 |
| Text |  | reach your daily step goal. Do you still feel this way? What steps are you taking to make sure you regularly walk? | 120 |
| Paragraph |  |  | 130 |
| Text | OAConWalk>=1 | In the survey, we asked you how much confidence you had when it came to walking more. You picked $int(OAConWalk) (0 is "not at all confident" and 10 is "extremely confident"). Take a minute to think about why you chose this number. Now that you have a few weeks under your belt, do you feel that your confidence has gone up? | 140 |
| Paragraph |  |  | 150 |
| Select | 1 |  | 151 |
| Text | count(WalkBarrierVeryTrue)>=3 or count(WalkBarrierTrue)>=3 | The suggestions listed below may help with something else you told us might keep you from regularly walking. Which, if any, is something you might be willing to do to help you reach your walking goals? | 160 |
| Text |  | Take a look at the suggestions below. If you feel like you are still struggling to meet your walking goals, which, if any, is something you might be willing to do to help you reach your goals? | 161 |
| EndSelect |  |  | 162 |
| Paragraph |  |  | 170 |
| Section | Page1Body2 |  | 181 |
| Insert |  | ../../Barriers.html | 190 |
| Section | Page1Body3 |  | 200 |
| Select | 1 |  |  |
| Text | count(WalkBarrierVeryTrue)>=4 or count(WalkBarrierTrue)>=4 | To learn solutions to another issue that you told us might prevent you from regularly walking, please go to the next page. | 210 |
| Text |  | To learn solutions to another issue that many new walkers face when it comes to getting into a regular walking routine, please go to the next page. | 220 |
| EndSelect |  |  |  |
| Section | Page2Header |  | 230 |
| Text |  | **Maintaining your progress...staying focused** | 240 |
| Evaluate |  | Barrier = Barrier4 |  |
| Section | Page2Body |  | 250 |
| Text |  | In this session, as well as in session 2, we've looked at 3 things that can get in the way of reaching your walking goals. | 270 |
| Select | 1 |  | 271 |
| Text | count(WalkBarrierVeryTrue)>=4 or count(WalkBarrierTrue)>=4 | Let's look at one last thing that you told us gets in your way. | 272 |
| Text |  | Let's look at one last thing that many people mention often get in their way. | 273 |
| EndSelect |  |  | 274 |
| Text |  | Remember, **focus on solutions** and come up with a game plan to stay on track so that you can enjoy long term success. | 275 |
| Paragraph |  |  | 280 |
| Section | Page2Body2 |  | 290 |
| Insert |  | ../../Barriers.html | 300 |
| Section | Page2Body3 |  | 310 |
| Block | BarColdWeath>=1 and BarColdWeath<=4 and Season(BaselineDate, 14)=="Winter" and Season(BaselineDate, 35)=="Winter" |  | 320 |
| Text |  | Does cold weather still keep you from reaching your daily step goal? Here are a few more tips that may help. | 330 |
| Select | 1 |  | 340 |
| Text | HeavyHousework=="Yes" | - **Increase your steps** in the comfort of your own home by getting some work done around the house. Chores like vacuuming or washing windows can be a great way to increase your steps. | 350 |
| Text | HomeRepairs=="Yes" | - **Increase your steps** in the comfort of your own home by getting some work done around the house. Get some of those needed house repairs finished up inside before the nice weather comes along so you don't have to be stuck painting the basement on a beautiful day. Doing repairs around the house can be a great way to increase your steps. | 360 |
| EndSelect |  |  | 370 |
| Text |  | - **Dress in layers**. Wear a hat and gloves. Wear a breathable fabric next to your skin, an insulating layer of wool or fleece and a windproof outer layer. You can always remove layers if you get too warm. | 380 |
| Text |  | - When walking outside in cold weather, **head away from the wind** as you start your walk. At the end of your walk, you're more likely to be warm from the exercise and more tolerant of the wind on your way back. | 390 |
| EndBlock |  |  | 400 |
| Block | BarHotWeath>=1 and BarHotWeath<=4 and Season(BaselineDate,14)=="Summer" and Season(BaselineDate,35)=="Summer" |  | 410 |
| Text |  | Is heat still a problem for you when it comes to walking? Here are a few more tips that may help. | 411 |
| Select | 1 |  | 420 |
| Text | isNumber(ManyPlacesWalk) and ManyPlacesWalk>=3 | - You told us that there are many places to go within easy walking distance of your home. **Walk to a nearby mall or store**. You can enjoy the air-conditioned building to keep you cool. | 430 |
| Text |  | - Take a trip to the **local grocery store** and walk the aisles. Don't forget to hit the refrigerated and frozen section aisles. They can feel great on a hot day. | 440 |
| EndSelect |  |  | 450 |
| Text |  | - Dress in light colored, **breathable fabrics**, such as nylon, when you walk. A breathable fabric can draw the sweat away from your skin so that the evaporation can **cool you down.** | 460 |
| Text |  | - **Wear a hat** to keep the sun off your head. It's also a good way to prevent sunburn. | 470 |
| EndBlock |  |  | 480 |
| Block | BarHotWeath<=4 and Season(BaselineDate,35)=="Summer" and not Season(BaselineDate,14)=="Summer" |  | 490 |
| Text |  | You also told us that hot weather occassionally keeps you from walking. Uncomfortable weather can keep even the most faithful walker inside. But, there are ways to keep cool even if the temperature is high. Explore the tips below to see if you can find a way to walk when it's hot. | 500 |
| Select | 1 |  | 510 |
| Text | LtHousework=="Yes" and HeavyHousework=="Yes" | - If it's just too hot to venture outside, **catch up on some housework**. Dusting, vacuuming, washing windows and walking back and forth to the washer and dryer will help increase your daily steps. | 520 |
| Text | LtHousework=="Yes" and HeavyHousework=="No" | - If it's just too hot to venture outside, **catch up on some housework**. Dusting and washing and putting away dishes can help you increase your daily steps. | 530 |
| Text | LtHousework=="No" and HeavyHousework=="Yes" | - If it's just too hot to venture outside, **catch up on some housework**. Vacuuming, scrubbing floors and washing windows can help increase your daily steps. | 540 |
| Text | LtHousework=="No" and HeavyHousework=="No" | - If it's just too hot to venture outside, **catch up on some housework**. Try dusting, vacuuming, or washing windows. All of these will help increase your daily steps. | 550 |
| Text |  | - If it's just too hot to venture outside, **do some housework**. Dusting and washing and putting away dishes can help you increase your daily steps. | 560 |
| EndSelect |  |  | 570 |
| Select | 1 |  | 580 |
| Text | BarExpensive>4 and Employment in ("FullTime", "PartTime") and Gender=="Male" | - If you're not already a member, **join a fitness club** near your job. Hit the treadmill or track immediately before or after work. You'll meet other people and avoid the stress of driving in rush-hour traffic. Plus, the cost may be an extra incentive to stick with the program. | 590 |
| Text | BarExpensive>4 and Employment in ("FullTime", "PartTime") and Gender=="Female" | - If you're not already a member, **join a fitness club** near your job. Sign up for a group exercise class that meets immediately before or after work. You'll meet other people and avoid the stress of driving in rush-hour traffic. Plus, the cost may be an extra incentive to stick with the program. | 600 |
| Text | BarExpensive>4 and Gender=="Female" | - **Join a fitness club** near your home. Sign up for a group exercise class that meets once a week. You'll get some great exercise and meet new people. Plus, the cost may be an extra incentive to stick with the program. | 610 |
| Text | BarExpensive>4 and Gender=="Male" | - **Join a fitness club** near your home. Hit the treadmill or track immediately before or after work. You'll get some great exercise and meet new people. Plus, the cost may be an extra incentive to stick with the program. | 620 |
| Text |  | - When it's hot outside, it makes sense to move your walks indoors. However, gyms can be expensive. How many gyms does your town have? Look into all of the local gyms in your area and **compare prices**. Perhaps you can join for a half a year or just for the summer. | 630 |
| EndSelect |  |  | 640 |
| Text |  | - Drink water to cool down at the beginning and end of your walk. You want to stay **well hydrated** so you don't overheat. | 650 |
| Text |  | - Walk in the early morning or in the evening around sunset to **avoid the hottest part of the day.** Choose shaded trails or neighborhoods with tree lined sidewalks. | 660 |
| Paragraph |  |  | 670 |
| EndBlock |  |  | 680 |
| Block | BarColdWeath<4 and Season(BaselineDate,35)=="Winter" and not Season(BaselineDate,14)=="Winter" |  | 690 |
| Text |  | You also told us that cold weather keeps you from walking. Cold weather may seem like a great excuse to skip your walk for the day. But you don't have to make excuses! It is possible to stay warm even if the temperatures are low. | 700 |
| Text | BarExpensive>4 | - **Join a local gym,** even if it's just for the winter months. Choose one that's convenient and geared toward your fitness level. Ask the staff to show you how to use the equipment. | 710 |
| Text | ChildInHome=="No" and not SF36LowIntAct in ("SomeLimit," "SigLimit") | - Is it snowing? **Snow can be fun**! Be a kid again. Throw snowballs and make snow angels or snowmen on your walk. | 720 |
| Text | ChildInHome=="Yes" and not SF36LowIntAct in ("SomeLimit," "SigLimit") | - Is it snowing? **With kids in the home**, snow can add a fun element to your walks. Join in on their fun. Throw snowballs and make snow angels or snowmen during your walk. | 730 |
| Text |  | - Make sure your shoes have **good traction**. You don't want to slip or fall on any icy patches. If the sidewalk does look icy, walk in the snowy grass. | 740 |
| Text |  | - Find a **warm place** to walk indoors. Walk at the mall, at an indoor track, or on a treadmill. | 750 |
| Text |  | - **Run some errands**. Head to a department or grocery store and walk the aisles. | 760 |
| Paragraph |  |  | 770 |
| EndBlock |  |  | 780 |
| Text |  | **What to expect next** | 781 |
| Paragraph |  |  | 782 |
| Text |  | As we come to the end of your fifth session, we hope you feel armed and ready to get out there and increase your daily steps. While you are here, don't forget to explore:  - Your new daily tip (and perhaps some of the daily tips you've missed). - Personalized graphs of your daily and weekly steps. And make sure you upload your pedometer! - All of your previous sessions for ideas and inspiration to keep walking. | 790 |
| Paragraph |  |  | 800 |
| Text |  | One week from now, you will receive your sixth, and final, personalized session. This session will tell a personal story of someone who has made walking a regular part of their life. Watch for an email message letting you know when it's available. | 810 |
| Select | 1 |  | 820 |
| Text | not isEmpty(AddressPref) | Until then, happy walking, $AddressPref! | 830 |
| Text | isEmpty(AddressPref) | Until then, happy walking! | 840 |
| EndSelect |  |  | 850 |
