# Supplementary material for: An Online Community Improves Adherence in an Internet-Mediated Walking Program. Part 1: Results of a Randomized Controlled Trial
Source: J Med Internet Res. 2010 Dec 17;12(4):e71. doi: 10.2196/jmir.1338 (PMC3056526; doi:10.2196/jmir.1338)
Supplement: Supplementary file 14 [file jmir_v12i4e71_app14.html]

WG6.html


SUH - Session 6, pages 1. Last Revision Sept 12, 2006

|  |  |  |  |
| --- | --- | --- | --- |
| **Command** | **Logic** | **Message** | **Row** |
| Comment |  | Goal of Section: Provide a story about an individual that is similar to what we have learned about the participant from his/her survey responses. The testimonial will convey the character's walking program experiences and barriers they've had to overcome. | 10 |
| Section | Page1Header |  | 11 |
| Select | 1 |  | 12 |
| Text | Gender=="Male" | **Stepping up - Chris's success story** | 13 |
| Text |  | **Stepping up - Deb's success story** | 14 |
| EndSelect |  |  | 15 |
| Section | Page1Body |  | 16 |
| Select | 1 |  | 17 |
| Text | Gender=="Male" | Chris | 20 |
| Text |  | Deb | 30 |
| EndSelect |  |  | 31 |
| Comment |  | $Gender will use "he" or "she" depending on gender - $cap(Gender) will use "He" or "She" | 40 |
| Text |  | was in your shoes a few months ago. $cap(Gender) hadn't been exercising very much, and felt it was time to become more active. | 50 |
| Paragraph |  |  | 55 |
| Text |  | **Why did you decide to become more active?** | 60 |
| Paragraph |  |  | 65 |
| Comment |  | Text below is intended to be conversational, and therefore may not be grammatically correct | 66 |
| Select | 1 |  | 70 |
| Text | "Golf" in EnjoyRecAct | I was out golfing with some friends and noticed I couldn't keep up with them like I used to. I had my annual check-up that next week, and when my doctor asked me what I was doing for exercise, I didn't have much of an answer. Since I like to golf, $Gender suggested I start walking the course instead of using the cart, at least for 9 holes to start. | 80 |
| Text | "Tennis" in EnjoyRecAct | A couple of friends asked if I wanted to get back into playing tennis with them like I used to, and I had to say no because I didn't think I could keep up with them. I had my annual check-up that next week, and when my doctor asked me what I was doing for exercise, I didn't have much of an answer. If I wanted to get out and play tennis, $Gender suggested that I do some walking to get some exercise and help me get back into shape. | 90 |
| Text | "Bike" in EnjoyRecAct | It's kind of embarrassing. One day while I was trying to find something in the garage, I pulled out my old bike and dusted it off. I tried taking it for a ride, but it was a lot harder for me than it should have been. I had my annual check-up that next week, and when my doctor asked me what I was doing for exercise, I didn't have much of an answer. If I wanted to take my bike out more often, $Gender suggested I do some walking to get some exercise and to help me get back into shape. | 100 |
| Text | "Bowl" in EnjoyRecAct | It's kind of embarrassing. Some friends asked if I wanted to go bowling one night, and I thought I'd give it a shot. The morning after, my body ached and I was exhausted. I had my annual check-up that next week, and when my doctor asked me what I was doing for exercise, I didn't have much of an answer. $cap(Gender) suggested I get out and bowl some more and also try walking to get some exercise. | 110 |
| Text | "Garden" in EnjoyRecAct | It's kind of embarrassing. I was out trying to save my garden that I'd neglected for too long, and I didn't get much done before I had to take a breather. I had my annual check-up that next week, and when my doctor asked me what I was doing for exercise, I didn't have much of an answer. Since I like gardening, $Gender suggested I do a little of that mixed with walking so that I get some exercise. | 120 |
| Text | WorkPayVol=="Yes" | It's kind of embarrassing. Some friends asked if I wanted to help out as a volunteer at the local museum giving tours, and I thought I'd give it a shot. During my volunteer training, we did all this walking and at the end of the day, I was just exhausted. I had my annual check-up that next week, and when my doctor asked me what I was doing for exercise, I didn't have much of an answer. If I wanted to be able to spend more time volunteering at the museum, $Gender suggested that I do some walking to get some exercise and help me get back into shape. | 130 |
| Text | SF36SevFlights=="SigLimit" or SF36OneFlight in ("SomeLimit", "SigLimit") | It's kind of embarrassing. One day I was rushing up the stairs to grab something from the bedroom and had to slow down because I was totally winded. I had my annual check-up that next week, and when my doctor asked me what I was doing for exercise, I didn't have much of an answer. $cap(Gender) said I should try walking to get some exercise. | 140 |
| Text | SF36LowIntAct in ("SomeLimit","SigLimit") | It's kind of embarrassing. I was getting the house ready for some friends to come visit, and I got so tired from cleaning up the kitchen that I had to sit down and take a break before I was halfway done. I had my annual check-up that next week, and when my doctor asked me what I was doing for exercise, I didn't have much of an answer. $cap(Gender) said I should try walking to get some exercise so that doing everyday things would be a little easier for me. | 150 |
| Text |  | It's kind of embarrassing. I was out doing some errands, and I rushed into a store from the parking lot to avoid the rain. When I got inside, I was totally winded and had to stand there to catch my breath. I had my annual check-up that next week, and when my doctor asked me what I was doing for exercise, I didn't have much of an answer. $cap(Gender) said I should try walking to get some exercise so that doing everyday things would be a little easier for me. | 160 |
| EndSelect |  |  | 170 |
| Text |  | That sounded nice and all, but when $Gender told me that walking could |  |
| Select | 1 |  | 180 |
| Text | CADSev in ("High", "Low") | reduce my chances of having more heart problems in the future, | 190 |
| Text | DIABSev in ("High", "Low") | make diabetes easier to live with, | 200 |
| Text |  | reduce my chances of having some health problems down the road, | 210 |
| EndSelect |  |  | 220 |
| Text |  | I decided it was time to get on board. | 230 |
| Paragraph |  |  | 240 |
| Text |  | **What got you to take those first steps?** | 250 |
| Paragraph |  |  | 260 |
| Block | Motivation1=="IMPreventFuture" |  | 270 |
| Text |  | Well, "an apple a day" certainly wasn't cutting it. I needed to exercise more. But what really got me going was when my doctor said walking could | 280 |
| Select | 1 |  | 290 |
| Text | CADSev in ("High", "Low") | reduce my chances of having more heart problems down the road. | 300 |
| Text | DIABSev in ("High", "Low") | prevent me from having more problems with my diabetes down the road. | 310 |
| Text | BMI>=30 | prevent me from having health problems because of my weight. | 320 |
| Text |  | prevent me from having serious health problems down the road. | 330 |
| EndSelect |  |  | 340 |
| Text |  | As much as I love going to the doctor, I'd rather spend my time somewhere else in the future. | 350 |
| EndBlock |  |  | 360 |
| Block | Motivation1 in ("IMManageWeight", "EMOthersDown", "EMDoctor", "NMRegret", "IMIncreaseEnergy", "EMOtherWant", "IMResponsible", "EMGdExFam", "IMEnjoy", "EMUpsetOthers", "MotAttractive") |  | 370 |
| Text | Motivation1=="IMManageWeight" | I couldn't keep my weight under control. I tried every diet plan out there, and they all ended up with the same result. One thing I hadn't tried was walking. I thought I might as well go for it, something had to work. Plus, | 380 |
| Text | Motivation1=="EMDoctor" | My doctor told me to get out and exercise more, at least a few times a week. $cap(Gender) pointed me in the right direction before, so I thought I'd give it a shot. Plus, | 390 |
| Text | Motivation1=="IMResponsible" | I had let myself go for the last few years, not exercising, eating a few too many french fries, and always leaving room for my favorite treat, ice cream. I just felt it was time to take responsibility for my health. Plus, | 400 |
| Text | Motivation1=="EMGdExFam" | I've had some pretty unhealthy habits over the years. I rarely exercised, and I chose fries over a salad every chance I got. How could I expect my family to eat healthy and exercise if I wasn't? I needed to set a good example for my family, so walking seemed like something I should do. Plus, | 410 |
| Text | Motivation1=="IMEnjoy" | Even though I hadn't been exercising much, I actually enjoy it sometimes. It makes me feel like I'm doing something good for myself. I don't know why I wasn't doing it more often. Anyway, walking seemed like a good way to get back on track. Plus, | 420 |
| Text | Motivation1=="NMRegret" | My doctor told me it was pretty important that I get some exercise. I knew that $Gender was probably right and that I'd regret it if I had | 430 |
| Select | 1 |  | 440 |
| Text | CADSev in ("High", "Low") and Motivation1=="NMRegret" | more problems with my heart down the road. Plus, | 450 |
| Text | DIABSev in ("High", "Low") and Motivation1=="NMRegret" | more problems with my diabetes down the road. Plus, | 460 |
| Text | BMI>=30 and Motivation1=="NMRegret" | more problems with my weight down the road. Plus, | 470 |
| Text | Motivation1=="NMRegret" | health problems down the road. Plus, | 480 |
| EndSelect |  |  | 490 |
| Text | Motivation1=="IMIncreaseEnergy" | I couldn't believe how tired I was feeling all the time. I wanted to get more done | 500 |
| Select | 1 |  | 510 |
| Text | Employment in ("FullTime", "PartTime") and Motivation1=="IMIncreaseEnergy" | after work, but I always fell short. | 520 |
| Text | Motivation1=="IMIncreaseEnergy" | around the house, but I always fell short. | 530 |
| EndSelect |  |  |  |
| Text | Motivation1=="IMIncreaseEnergy" | My doctor said getting some exercise could help give me more energy, which didn't make much sense to me at first. I thought it'd make me more tired. But things weren't getting any better, so I gave it a shot. Plus, | 540 |
| Text | Motivation1 in ("EMOtherWant", "EMUpsetOthers", "EMOthersDown") | When I got home from the doctor, I let it slip | 550 |
| Select | 1 |  | 560 |
| Text | MaritalStatus=="Married" and Gender=="Male" and Motivation1 in ("EMOtherWant", "EMUpsetOthers", "EMOthersDown") | to my wife that I was encouraged to exercise more. She | 570 |
| Text | MaritalStatus=="Married" and Gender=="Female" and Motivation1 in ("EMOtherWant", "EMUpsetOthers", "EMOthersDown") | to my husband that I was encouraged to exercise more. He | 580 |
| Text | MaritalStatus=="Partner" and Motivation1 in ("EMOtherWant", "EMUpsetOthers", "EMOthersDown") | that I was encouraged to exercise more. My partner | 590 |
| Text | Motivation1 in ("EMOtherWant", "EMUpsetOthers", "OthersDown") | to my friend, Joanne, that I was encouraged to exercise more. She | 600 |
| EndSelect |  |  | 610 |
| Text | Motivation1 in ("EMOtherWant", "EMUpsetOthers", "EMOthersDown") | really wanted me to take the advice. I don't like letting others down, so I decided to give it a shot. Plus, | 620 |
| Text | TryingLoseWt=="Yes" and Motivation1=="MotAttractive" | I had been trying to lose weight for awhile, so walking seemed like something that could really help me. | 630 |
| Text | (TryingLoseWt=="No" or isEmpty(TryingLoseWt)) and Motivation1=="MotAttractive" | When my doctor told me about exercising and walking more, it sounded like something good for me. | 640 |
| Text | Motivation1=="MotAttractive" | If I managed my weight, I knew I'd feel better about myself and the way I looked. Plus, | 650 |
| Select | 1 |  | 660 |
| Text | "Golf" in EnjoyRecAct | golf season was coming up, and I wanted to be able to keep up with my friends. | 670 |
| Text | "Tennis" in EnjoyRecAct | I wanted to get out and play tennis more often. | 680 |
| Text | "Bike" in EnjoyRecAct | I wanted to get out and ride my bike more often. | 690 |
| Text | "Bowl" in EnjoyRecAct | bowling leagues were getting ready to start. | 700 |
| Text | "Garden" in EnjoyRecAct | I wanted to be able to work in my garden more often. | 710 |
| Text | WorkPayVol=="Yes" | I wanted to be able to volunteer more often and give tours at the museum. | 720 |
| Text |  | I had a class reunion coming up and I wanted to look good. | 730 |
| EndSelect |  |  | 740 |
| EndBlock |  |  | 750 |
| Text | Motivation1=="EMOthersSee" | Some of my friends like to give me a hard time, so when I told them I was thinking about exercising more, they said they'd believe it when they saw it. I thought I'd prove them wrong and show them I was serious about this walking thing. I couldn't wait to see the look on their faces when they saw what I was doing. | 760 |
| Text | Motivation1=="NMGuilt" | I'd be driving around town and see all these people out on a run or walking. After awhile, the guilt got to me. I wasn't exercising at all and I needed to make a change so that I wasn't reminded of that every time I drove around town. | 770 |
| Text | Motivation1=="IMAccomplishment" | After talking to my doctor, I knew I had to do something to get some exercise. I promised myself I'd walk 3 days a week to start and see how things went from there. I gave myself a reward after that first week, a trip to the local ice cream shop. It felt really good to accomplish what I'd set out to do. | 780 |
| Block | Motivation1=="MotControlExisting" |  | 790 |
| Text |  | For a while I'd been looking for something that I could do to help | 800 |
| Select | 1 |  | 810 |
| Text | CADSev in ("High", "Low") | control my heart disease. | 820 |
| Text | DIABSev in ("High", "Low") | control my diabetes. | 830 |
| Text | BMI >=30 | control my weight. | 840 |
| Text | "Arthritis" in HealthHistory | with my arthritis pain. | 850 |
| Text | "Osteo" in HealthHistory | with my osteoporosis. | 860 |
| Text | "HBP" in HealthHistory | manage my blood pressure. | 870 |
| Text | "HiChol" in HealthHistory | lower my cholesterol levels. | 880 |
| Text |  | keep me healthy. | 890 |
| EndSelect |  |  | 900 |
| Text |  | When my doctor suggested the walking thing, I thought I'd give it a shot. I promised myself I'd walk 3 days a week to start and see how things went from there. | 910 |
| EndBlock |  |  | 920 |
| Block | Motivation1=="IMDailyAct" |  | 930 |
| Text |  | It's tough to admit, but trying to do things like | 940 |
| Select | 1 |  | 950 |
| Text | LtHousework=="Yes" | washing dishes after a big meal | 960 |
| Text | HeavyHousework=="Yes" | washing the floors | 970 |
| Text | LawnWork=="Yes" | working out in the yard | 980 |
| Text | HomeRepairs=="Yes" | painting | 990 |
| Text |  | cleaning up the house and running errands | 1000 |
| EndSelect |  |  | 1010 |
| Text |  | were no longer that easy for me. I was tired of having trouble with those everyday things. My doctor said walking could help, so I thought I'd give it a shot. | 1020 |
| EndBlock |  |  | 1030 |
| Text | Motivation1=="NMBadAboutSelf" | A lot of things weren't going my way. My car had just gotten rear ended, I had spent the last week with the flu, and I had just eaten the last of the ice cream in the freezer. I didn't need to feel bad about anything else, so I tried to get out and walk a little like my doctor said. | 1040 |
| Block | Motivation1=="MotLessTension" |  | 1050 |
| Select | 1 |  | 1060 |
| Text | Employment in ("FullTime", "PartTime") | Some co-workers had just been layed off and things were pretty tense at work, so when my doctor told me that walking could help me deal with the stress, I was all ears. At first I was skeptical that it would really help, but I knew I had to try something to get me through the day and ease my mind. | 1070 |
| Text |  | My life was pretty stressful at the time. My mom had just been diagnosed with alzheimer's, and I was spending a lot of time trying to figure out where she was going to live. I just needed to try something to get me through the day and ease my mind. When my doctor suggested walking, I didn't know if it'd work or not, but I was up for trying anything. | 1080 |
| EndSelect |  |  | 1090 |
| EndBlock |  |  | 1100 |
| Block | Motivation1=="IMPhysHealth" |  | 1110 |
| Text |  | The talk with my doctor hit me pretty hard. $cap(Gender) made it clear that I needed to be in better physical shape if | 1120 |
| Select | 1 |  | 1130 |
| Text | "Golf" in EnjoyRecAct | I wanted to get out and play golf more often. | 1140 |
| Text | "Tennis" in EnjoyRecAct | I wanted to get out and play tennis more often. | 1150 |
| Text | "Bike" in EnjoyRecAct | I wanted to get out and ride my bike more often. | 1160 |
| Text | "Bowl" in EnjoyRecAct | I wanted to get out and bowl more often. | 1170 |
| Text | "Garden" in EnjoyRecAct | I wanted to be able to work in my garden more often. | 1180 |
| Text |  | I wanted to be able to do daily things without getting tired out. | 1190 |
| EndSelect |  |  | 1200 |
| Text |  | I knew I had to make a change and add some exercise to my day. It wasn't easy, but I got out and tried walking. | 1210 |
| EndBlock |  |  | 1220 |
| Text | Motivation1=="EMGdExCom" | Some people in my neighborhood are more likely to use their car to pick up the mail down the street rather than just walk to pick it up. I thought it was about time that someone tried to set a good example in the community by walking and being active. | 1230 |
| Text | Motivation1=="IMBelieve" | A lot of things weren't going my way. My car had just gotten rear ended, I had spent the last week with the flu, and I had just eaten the last of the ice cream in the freezer. I needed to do something positive that I could feel good about, so I tried to get out and walk a little like my doctor said. | 1240 |
| Text | Motivation1=="EMTold" | I knew that I should probably be exercising more, but I could never decide on what to do. One of my friends was doing pilates, another an aerobics class. I even had a neighbor doing that yoga thing. When I talked to my doctor, $Gender said walking would do me just fine. It sure was easier for me to follow doctor's orders than trying to figure out what would work for me. | 1250 |
| Paragraph |  |  | 1260 |
| Text |  | **How was it starting out?** | 1270 |
| Paragraph |  |  | 1280 |
| Block | Barrier1=="BarTime" or Barrier1=="BarTired" |  | 1300 |
| Text |  | I had a rocky start. Years ago, I used to work out in the evening. But now when I | 2700 |
| Select | 1 |  | 2710 |
| Text | "FullTime" in Employment or "PartTime" in Employment | get home from a long day at work, | 2720 |
| Text | "Homemaker" in Employment | get through a long day of running errands and cleaning up the house, | 2730 |
| Text | "Student" in Employment | get home from a long day of classes, | 2740 |
| Text | "UnLooking" in Employment | get through a long day of looking for a job, | 2750 |
| Text |  | get through a long day of running errands around town, | 2760 |
| EndSelect |  |  | 2770 |
| EndBlock |  |  | 2771 |
| Block | Barrier1=="BarTime" |  | 2780 |
| Text |  | I don't really have time for a walk. I have | 2790 |
| Select | 1 |  | 2800 |
| Text | Gender=="Male" | bills to pay or the yard to take care of, and something always seems like it needs fixing. | 2810 |
| Text | Gender=="Female" | dinner to make and need to get the house in order. | 2820 |
| Text |  | bills to pay and dinner to make. | 2830 |
| EndSelect |  |  | 2840 |
| Text |  | Plus, at some point I just want to | 2850 |
| EndBlock |  |  | 2860 |
| Block | Barrier1=="BarTime" or Barrier1=="BarTired" |  |  |
| Text | Barrier1=="BarTired" | the last thing I want to do is go out on a walk. I'd rather have a good dinner and | 2870 |
| Select | 1 |  | 2970 |
| Text | "TV" in EnjoyRecAct | relax in front of the TV. | 2980 |
| Text | "Read" in EnjoyRecAct | relax with a good book. | 2990 |
| Text | MaritalStatus=="Married" and Gender=="Male" | relax with my wife. | 3000 |
| Text | MaritalStatus=="Married" and Gender=="Female" | relax with my husband. | 3010 |
| Text | MaritalStatus=="Partner" | relax with my partner. | 3020 |
| Text | "Cards" in EnjoyRecAct | relax and play some cards. | 3030 |
| Text |  | relax before going to bed. | 3040 |
| EndSelect |  |  | 3050 |
| Text | Barrier1=="BarTime" | It wasn't easy, but I made sure to always schedule some time in for a walk on a lunch break. It actually worked out pretty nice to break up my busy day, and instead of making me feel more rushed, the walking slowed the pace of my day down. | 3055 |
| Text | Barrier1=="BarTired" | It wasn't easy, but I decided to wake up a little earlier and walk first thing in the morning. It was hard to do at first, but after a month, it became pretty routine. The bonus - evenings open for other things. | 3060 |
| EndBlock |  |  | 3070 |
| Block | Barrier1=="BarHealthProb" |  | 3080 |
| Select | 1 |  | 3090 |
| Text | CADExAfraid=="Yes" | I had a rocky start because I was worried about my heart disease. At the beginning of one of my first walks, I felt sick to my stomach, and I had to stop and take a seat. It was probably nerves more than anything, but it kept me from walking for another week. When I got the courage to start up again, I took it slow and made sure to rest when I got tired. Thankfully, I didn't experience any major problems, and I've been walking ever since. | 3100 |
| Text | CADSev in ("High", "Low") and CADSwell>=3 | I had a rocky start because I was worried about my ankles swelling up. It had happened to me before, and it was a pain, literally. Sure enough, after one of my walks my feet were hurting and I looked down to see some swelling. I quickly made sure to elevate my feet and thankfully the swelling went down. I went to see my doctor and $Gender told me that things would be ok as long as I took it slow. Things have gone pretty well since then. | 3120 |
| Text | CADSev in ("High", "Low") | I had a rocky start because I didn't know what to expect with my heart disease. It was probably nerves more than anything. I wasn't used to exercise, so I took it slow and I sat down and rested sometimes because I got tired. After awhile I got more comfortable with how my body reacted to exercise, so that made things easier. Thankfully, I didn't experience any major problems, and I've been walking ever since. | 3130 |
| Text | DIABSev in ("High", "Low") and DiabFtProb>=4 | I had a rocky start. My feet had given me problems in the past, and I was worried they'd still be an issue. Sure enough, I got a blister on one of my first walks. I talked to my doctor about it, and $Gender told me how important it was that I stay active and check my feet for sores everyday to make sure things didn't get worse. $cap(Gender) even made sure the socks and shoes I was wearing fit right. I was nervous to start up again, so it took me a couple of weeks to get back on track. | 3140 |
| Text | DIABSev in ("High", "Low") and DiabMonthLowSug in ("1to3Times", "4to6Times", "7to12Times", "More12Times") or DiabYrSvrLowSug in ("1to3Times", "4to6Times", "7to12Times", "More12Times") or DiabMonthHighSug in ("1to3Times", "4to6Times", "7to12Times", "More12Times") | I had a rocky start. I wasn't sure what my blood sugar level would do when I exercised. I followed the doctor's orders, starting out slowly during the first couple of weeks, and I made sure to monitor my blood sugar before and after exercising just to make sure I was doing ok. After awhile, I got a feel for how my body reacted to exercise, so thankfully I didn't have to check my blood nearly as much, that was getting old. Even though it was a pain to start out, I'm glad I did what I did because I don't think I'd be comfortable taking long walks like I do now. | 3150 |
| Text | DIABSev in ("High", "Low") | I had a rocky start. I wasn't sure how exercising would go with my diabetes. Following doctor's orders, I started slow and checked my feet for sores after walking. I also checked my blood sugar quite a bit at first, just to be safe. It was a hassle at first, but I did get more comfortable with walking. I got a blister after a walk one day, which set me back a bit, but everything went pretty well after that. | 3160 |
| Text | "Arthritis" in HealthHistory | I had a rocky start because I was worried about my arthritis flaring up. That was sure to make every day more uncomfortable. I was sore after my first few walks, and I thought that was going to be it for me and walking. But I decided to take my doctor's advice and try for a little longer. Sure enough, my knees and ankles loosened up a bit and it wasn't nearly as hard on me as it was starting out. | 3170 |
| Text | "Asthma" in HealthHistory | I had a rocky start. On one of my first walks, I got out of breath and had to take a seat. It was a good thing I had my inhaler with me, I probably would have panicked otherwise. That scared me and kept me from walking for a couple of weeks. I talked to my doctor about it, and $Gender said to take it slow and give it another shot. I tried again, and thankfully I didn't have any big problems. After a couple of weeks, it was much easier to head out on a walk without worrying so much. | 3180 |
| Text | "Osteo" in HealthHistory and not Barrier2=="BarNoOne" | I had a rocky start. With osteoporosis, a fall would be real bad, and so I didn't get out to walk much at all at first. When a friend told me to walk at the local high school track because it was flat, that seemed to make things easier. I started out slowly and the more I walked, the more comfortable I got with it. During the week, I also asked friends to join me, both to make sure I didn't fall and to keep me company. | 3190 |
| Text | "Osteo" in HealthHistory | I had a rocky start. With osteoporosis, a fall would be real bad, and so I didn't get out to walk much at all at first. When a friend told me to walk at the local high school track because it was flat, that seemed to make things easier. I started out slowly and the more I walked, the more comfortable I got with it. | 3200 |
| Text | "HBP" in HealthHistory | I had a rocky start because I was worried about my high blood pressure. One time I went in for a check-up, and it was pretty high, so that was always in the back of my mind. I sure didn't feel like going for a walk and risking it getting worse. But then I got myself one of those blood pressure gadgets so I could check it at home, and that made me feel more comfortable. I took it slow, and after a couple weeks, I really wasn't thinking about my blood pressure anymore when I was walking. | 3230 |
| Text |  | I had a rocky start. I hadn't been exercising at all, so I wasn't sure how it was going to go. On my first walk I think I over did it because I was exhausted afterwards and the next morning I woke up pretty sore. I sure didn't feel like going out on a walk after that. I ended up giving it a try again a week later, and took it a little slower. I was a little sore the next morning, but not nearly as bad. I kept up with it from there, and things went pretty well. | 3240 |
| EndSelect |  |  | 3250 |
| EndBlock |  |  | 3260 |
| Text | Barrier1=="BarEnergy" | I had a rocky start. I didn't really feel like I had the energy to get out and walk enough to make it worthwhile. I started really slow, or actually, extremely slow. I was only able to get out for a walk 1 time in the first 2 weeks. After that, I made an effort to walk at least 3 times in the next week. Somehow I was able to do it, and I was surprised to feel like I had more energy than before. I think it helped that I decided to walk in the morning. I usually have more energy then anyway. | 3290 |
| Text | Barrier1=="BarHurt" | I had a rocky start. I hadn't exercised much in a while and I didn't want to hurt myself. I started out a little slow and I did have some muscle soreness after the first few walks. My doctor suggested that I do some stretches after my walks, and that really seemed to help out. Getting by those first couple of weeks was important for me. Now it's just part of the daily routine. | 3300 |
| Block | Barrier1=="BarEffort" |  | 3301 |
| Text |  | I had a rocky start. Walking around the neighborhood was a little boring for me. I had a hard time increasing my number of steps without going out on longer walks, which I really didn't feel like doing. That's why I was surprised to see such a high step count one day because I hadn't gone for a long walk. I realized the steps must have come from | 3302 |
| Select | 1 |  | 3303 |
| Text | LawnWork=="Yes" | being outside raking all the leaves that had been covering our yard. I couldn't believe that I could get that many steps in just by doing work around the yard. | 3304 |
| Text | HomeRepairs=="Yes" | the time I spent painting the trim on the house. I had no idea I could get that many steps in just by doing some work around the house. | 3305 |
| Text | LtHousework=="Yes" or HeavyHousework=="Yes" | the time I spent cleaning. It had rained for most of the week, and I decided to get some much needed cleaning done inside the house. I had no idea I could get that many steps in just by doing some work around the house. | 3306 |
| Text |  | the time I spent cleaning up the house before a family get-together. I had no idea I could get that many steps in just by doing a little cleaning around the house. | 3307 |
| EndSelect |  |  | 3308 |
| EndBlock |  |  | 3309 |
| Text | Barrier1=="BarBadMood" | I had a rocky start. I was never really in the mood to get out and go for a walk. I tended to have lots of stuff on my mind and walking sure wasn't one of them. One day I wasn't feeling to good about myself sitting around the house, so I needed to get out and do something. I ended up taking a walk. It felt good to get out of the house, and when I got back, whatever was bothering me just didn't seem that bad anymore. I got into a walking routine and those bad days didn't seem to come around quite so often anymore. | 3310 |
| Text | Barrier1=="BarExpensive" | When my doctor told me it'd be good for me if I exercised a little more, I thought I'd have to join a gym or some kind of health club. I really didn't want to spend the money for that, so it took me a while to get started. One day I just clipped on the pedometer and tried to be active around the house doing some cleaning and then I went out for a short walk. I was really surprised to see how many steps I ended up getting in without even going to a gym to exercise. | 3320 |
| Block | Barrier1=="BarSafePlace" |  | 3330 |
| Text |  | I had a rocky start because | 3340 |
| Select | 1 |  | 3350 |
| Text | SafeWalkNight>=1 and SafeWalkNight<=2 and SafeWalkDay>=1 and SafeWalkDay<=2 | my neighborhood isn't really the safest place to take a walk during the day, and definitely not at night. | 3360 |
| Text | SafeWalkNight>=1 and SafeWalkNight<=2 | my neighborhood isn't really the safest place to take a walk, especially at night. | 3370 |
| Text | SafeWalkDay>=1 and SafeWalkDay<=2 | my neighborhood isn't really the safest place to take a walk during the day. | 3380 |
| Text | SidewalksSafe>=1 and SidewalksSafe<=2 | I didn't feel comfortable walking on the sidewalks and paths around my neighborhood. They're really poorly taken care of. | 3390 |
| Text |  | I didn't really feel that I had a safe place to exercise. | 3420 |
| EndSelect |  |  | 3430 |
| Text |  | So, I had to think of creative ways to increase my steps. On some days I would go to the local mall to walk around. Other times, I just found ways to be active | 3440 |
| Select | 1 |  | 3450 |
| Text | Employment in ("FullTime","PartTime") | at work by taking some extra trips to the water fountain for a drink and parking my car further away from the office. I even stopped sending as many emails as I used to and just walked down to my co-workers offices to ask them a question. | 3460 |
| Text |  | by doing a little extra work around the house. Even though I didn't get as many steps in while I was outside compared to walking, I was doing something. My house has sure benefited though. The yard looks great and I can actually get in the garage again. | 3470 |
| EndSelect |  |  | 3480 |
| EndBlock |  |  | 3490 |
| Text | Barrier1=="BarSweat" | I had a rocky start. I hate to sweat and that's the first thing that came to mind when I thought about exercise. Needless to say, I was hesistant to even start. But my doctor told me that I didn't have to sweat to benefit from walking, so I gave it a shot. I found out pretty quickly that if I did my walking either in the morning or night when it was cooler out, I didn't sweat as much. I tried to reward myself after a long walk in warmer weather with a nice cool shower when I could. | 3500 |
| Block | Barrier1=="BarNoOne" and not WantSupport=="No" |  | 3510 |
| Text |  | I had a rocky start, especially since I really didn't want to go for a walk by myself. | 3520 |
| Select | 1 |  | 3530 |
| Text | "Spouse" in WhoSupport and Gender=="Male" and MaritalStatus=="Married" | When I talked to my wife about what I was going to do, she actually offered to join me on some of my walks. It was just what I needed. | 3540 |
| Text | "Spouse" in WhoSupport and Gender=="Female" and MaritalStatus=="Married" | When I talked to my husband about what I was going to do, he actually offered to join me on some of my walks. It was just what I needed. | 3550 |
| Text | "Spouse" in WhoSupport and MaritalStatus=="Partner" | My partner recognized I was struggling to get going and actually offered to join me on some of my walks. It was just what I needed. | 3560 |
| Text | WantSupport=="Yes" and "Friend" in WhoSupport | When I talked to my friends about what I was going to do, they actually offered to join me on some of my walks. It was just what I needed. | 3570 |
| Text | "Coworker" in WhoSupport | When I talked to some co-workers about what I was going to do, they actually offered to join me on some of my walks. We agreed to fit in a walk at lunch, which turned out to be a great way to break up the day. | 3580 |
| Text |  | When I talked to some neighbors about what I was going to do, they actually offered to join me on some of my walks. It was just what I needed. | 3590 |
| EndSelect |  |  | 3600 |
| Text |  | It made it a little easier having someone there to talk to as I walked. | 3610 |
| EndBlock |  |  | 3620 |
| Text | Barrier1=="BarNoOne" and WantSupport=="No" | I had a rocky start. It was hard to get going when I didn't have anyone to walk with. One day I was chatting with some neighbors about what I was going to do and they actually offered to join me on some of my walks. It was kind of nice having someone to talk to as I walked. | 3630 |
| Text | Barrier1=="BarSelfConscious" | I had a rocky start. I didn't really want to exercise in public, so I tried finding places to walk where no one was around, but that plan didn't work out very well. Eventually, I realized that what I was doing for myself was more important than what others thought about me. The funny thing is, once I started walking, I noticed there were others out there exercising that looked like me. I didn't stick out as much as I thought I would! | 3640 |
| Paragraph |  |  | 3643 |
| Text |  | **Did you have help from anyone?** | 3650 |
| Paragraph |  |  | 3651 |
| Text | Barrier1=="BarNoOne" and not WantSupport=="No" | Like I said, I had a hard time starting out, and it sure was nice to have someone to give me a boost. I don't know if I would've been able to do it otherwise. | 3660 |
| Text | Barrier1=="BarNoOne" and WantSupport=="No" | Like I said, I had a hard time starting out, and I didn't think I'd need it, but it sure was nice to have my neighbors give me a boost. I don't know if I would've been able to do it otherwise. | 3670 |
| Text | Barrier2=="BarNoOne" and not WantSupport=="No" | You bet. Things got easier after my rough start, but then I hit a bump in the road a few weeks later. That's when I got the help I was looking for. | 3680 |
| Text | Barrier2=="BarNoOne" and WantSupport=="No" | You bet. Even though I had a hard time starting out, I really didn't feel like I needed any help to get going. That changed a few weeks later when things began to get tough. | 3690 |
| Block | not WantSupport=="No" and (not Barrier1=="BarNoOne" and not Barrier2=="BarNoOne") |  | 3700 |
| Text |  | You bet. When I really didn't feel like getting out there, | 3710 |
| Select | 1 |  | 3720 |
| Text | "Spouse" in WhoSupport and MaritalStatus=="Married" and Gender=="Male" | my wife helped keep me on track. She's an early riser, so seeing her dressed and ready on those mornings that I had a hard time getting out the door | 3730 |
| Text | "Spouse" in WhoSupport and MaritalStatus=="Married"and Gender=="Female" | my husband helped keep me on track. He's an early riser, so seeing him dressed and ready on those mornings that I had a hard time getting out the door | 3740 |
| Text | "Spouse" in WhoSupport and MaritalStatus=="Partner" | my partner helped keep me on track. Seeing someone dressed and ready on those mornings that I had a hard time getting out the door | 3750 |
| Text | "Child" in WhoSupport | my children helped keep me on track. On the weekend they'd be ready at my door when I was having a tough time getting moving, and it | 3760 |
| Text | "Friend" in WhoSupport and Gender=="Male" | my pal Eric helped keep me on track. Some mornings he'd be ready at my door when I was having a tough time getting moving, and it | 3770 |
| Text | "Friend" in WhoSupport and Gender=="Female" | my friend Sonya helped keep me on track. Some mornings she'd be ready at my door when I was having a tough time getting moving, and it | 3780 |
| Text | "Coworker" in WhoSupport and Gender=="Male" | my co-worker, Alan, helped keep me on track. Some days when I was having a tough time getting moving, he'd give me a boost, and it | 3790 |
| Text | "Coworker" in WhoSupport and Gender=="Female" | my co-worker, Nancy, helped keep me on track. Some days when I was having a tough time getting moving, she'd give me a boost, and it | 3800 |
| Text | "Sister" in WhoSupport | my sister, Barb, helped keep me on track. Some mornings she'd be ready at my door when I was having a tough time getting moving, and it | 3810 |
| Text | "Brother" in WhoSupport | my brother, Steve, helped keep me on track. Some mornings he'd be ready at my door when I was having a tough time getting moving, and it | 3820 |
| Text | "Other" in WhoSupport and Gender=="Male" | my cousin, Paul, helped keep me on track. Some mornings he'd be ready at my door when I was having a tough time getting moving, and it | 3830 |
| Text | "Other" in WhoSupport and Gender=="Female" | my cousin, Trish, helped keep me on track. Some mornings she'd be ready at my door when I was having a tough time getting moving, and it | 3840 |
| Text | "Mom" in WhoSupport | my mom helped keep me on track. Some mornings she'd be ready at my door when I was having a tough time getting moving, and it | 3850 |
| Text | "Dad" in WhoSupport | my dad helped keep me on track. Some mornings he'd be ready at my door when I was having a tough time getting moving, and it | 3860 |
| Text | Gender=="Male" | my pal Eric helped keep me on track. Some mornings he'd be ready at my door when I was having a tough time getting moving, and it | 3870 |
| Text |  | my friend Sonya helped keep me on track. Some mornings she'd be ready at my door when I was having a tough time getting moving, and it | 3880 |
| EndSelect |  |  | 3890 |
| Text |  | was just enough to keep me going. It was nice to have someone to walk with or just get encouragement from. | 3900 |
| EndBlock |  |  | 3910 |
| Text | WantSupport=="No" and (not Barrier1=="BarNoOne" and not Barrier2=="BarNoOne") | When I started out, I didn't really want any help. I thought I'd be fine on my own. But then a few weeks into the program I was struggling to keep up. When I went to get some new shoes at the mall one day, the salesman asked me where I went walking around town. He was a walker too, and he gave me some new ideas for some walking trails in the area that I'd never been to. The trails were great, they made long walks seem short! | 3920 |
| Paragraph |  |  | 3921 |
| Text |  | **Did you have a hard time keeping up with the program?** | 3930 |
| Paragraph |  |  | 3931 |
| Select | 1 |  | 3932 |
| Block | (Barrier2=="BarTime" and (not Barrier1=="BarTired")) or (Barrier2=="BarTired" and (not Barrier1=="BarTime")) |  | 3940 |
| Text |  | Sometimes it was tough to keep going. Years ago, I used to workout in the evening. But now when I | 3950 |
| Select | 1 |  | 3960 |
| Text | Employment in ("FullTime","PartTime") | get home from a long day at work, | 3970 |
| Text | "Homemaker" in Employment | get through a long day of running errands and cleaning up the house, | 3980 |
| Text | "Student" in Employment | get home from a long day of classes, | 3990 |
| Text | "UnLooking" in Employment | get through a long day of looking for a job, | 4000 |
| Text |  | get through a long day of running errands around town, | 4010 |
| EndSelect |  |  | 4020 |
| Text | (Barrier2=="BarTime" and (not Barrier1=="BarTired")) and Gender=="Male" | I don't really have time for a walk. I have bills to pay or the yard to take care of, and something always seems like it needs fixing. Plus, at some point I just want to | 4040 |
| Text | (Barrier2=="BarTime" and (not Barrier1=="BarTired")) and Gender=="Female" | I don't really have time for a walk. I have dinner to make and need to get the house in order. Plus, at some point I just want to | 4050 |
| Text | (Barrier2=="BarTime" and (not Barrier1=="BarTired")) and isEmpty(Gender) | I don't really have time for a walk. I have bills to pay and dinner to make. Plus, at some point I just want to | 4060 |
| Text | (Barrier2=="BarTired" and (not Barrier1=="BarTime")) | the last thing I want to do is go out on a walk. I'd rather have a good dinner and | 4120 |
| Select | 1 |  | 4130 |
| Text | "TV" in EnjoyRecAct | relax in front of the TV. | 4140 |
| Text | "Read" in EnjoyRecAct | relax with a good book. | 4150 |
| Text | MaritalStatus=="Married" and Gender=="Male" | relax with my wife. | 4160 |
| Text | MaritalStatus=="Married" and Gender=="Female" | relax with my husband. | 4170 |
| Text | MaritalStatus=="Partner" | relax with my partner. | 4180 |
| Text | "Cards" in EnjoyRecAct | relax and play some cards. | 4190 |
| Text |  | relax before going to bed. | 4200 |
| EndSelect |  |  | 4210 |
| Text |  | It wasn't easy, but I decided to wake up a little earlier and walk first thing in the morning. I'm not lying when I say it was hard to do at first. But after a month of working at it, it started to become somewhat routine. The bonus - my evenings weren't disturbed. | 4220 |
| EndBlock |  |  | 4230 |
| Block | Barrier2=="BarHealthProb" or Barrier3=="BarHealthProb" |  | 4360 |
| Select | 1 |  | 4370 |
| Text | CADSev in ("High", "Low") and CADSwell>=4 | Sometimes it was tough to keep going. One time after walking my ankles swelled up and that didn't feel too great. I made sure to elevate my feet and thankfully, the swelling went down. I sure didn't feel like going for a walk after that. I went to see my doctor and $Gender told me that things should be ok as long as I took it slow and $Gender was right, things have gone pretty well since then. | 4380 |
| Text | CADSev in ("High", "Low") | Sometimes it was tough to keep going because I didn't know what to expect with my heart disease. It was probably nerves more than anything. I wasn't used to exercise, so I took it slow and sat down and rested sometimes because I got tired. After awhile, I got more comfortable with how my body reacted to exercise, so that made things easier. Thankfully, I didn't experience any major problems, and I've been walking ever since. | 4390 |
| Text | DIABSev in ("High", "Low") and DiabMonthLowSug in ("1to3Times", "4to6Times", "7to12Times", "More12Times") or DiabYrSvrLowSug in ("1to3Times", "4to6Times", "7to12Times", "More12Times") or DiabMonthHighSug in ("1to3Times", "4to6Times", "7to12Times", "More12Times") | Sometimes it was tough to keep going because I was worried about my blood sugar level. It's probably because one time after a walk my blood sugar got real low and that made me real nervous. That episode set me back for a week or so until I got the courage to give it another shot. It helped when I called my doctor about it and $Gender gave me some advice about taking my meds. That settled my nerves a bit and things have gone pretty well since then. | 4400 |
| Text | DIABSev in ("High", "Low") and DiabFtProb>=4 and Age>50 | Sometimes it was tough to keep going because I had problems with my feet. I talked to my doctor about it, and $Gender told me to check my feet for sores everyday to make sure things didn't get worse. $cap(Gender) even made sure that the socks and shoes I was wearing fit right. $cap(Gender) also said I should try mixing in some other kinds of exercise into my routine that would put less stress on my feet. I tried riding a bike and my feet didn't seem to have a problem with it. And, as a bonus, riding the bike made me feel like a kid again. | 4410 |
| Text | DIABSev in ("High", "Low") and DiabFtProb>=4 and Age>1 and Age<=50 | Sometimes it was tough to keep going because I had problems with my feet. I talked to my doctor about it, and $Gender told me to check my feet for sores everyday to make sure things didn't get worse. $cap(Gender) even made sure that the socks and shoes I was wearing fit right. $cap(Gender) also said I should try mixing in some other kinds of exercise into my routine that would put less stress on my feet. I tried riding a bike and my feet didn't seem to have a problem with it. And, as a bonus, riding the bike was a lot of fun. | 4411 |
| Text | DIABSev in ("High", "Low") | Sometimes it was tough to keep going, especially with my diabetes. One time when I got home from a walk, my blood sugar was really low, and it got me all worried. I didn't go out again for another week. I asked my doctor what I should do, and $Gender gave me some advice about taking my meds. I tried it out, and it seemed to work pretty well. I haven't had many problems since, and I'm way more comfortable walking than I was before. | 4420 |
| Text | "Arthritis" in HealthHistory | Sometimes it was tough to keep going, especially when I had my arthritis flare up. One time my knee was hurting me pretty bad, so I just quit walking for a couple of weeks. After talking to my doctor, $Gender told me I should give it another shot and see how I feel. I decided to go for it, and it didn't end up being too bad. Sometimes I still have some soreness, but I've lost a little bit of weight so I think walking has been worth it. | 4430 |
| Text | "Asthma" in HealthHistory | Sometimes it was tough to keep going. On one of my walks, I got out of breath and had to take a seat. It was a good thing I had my inhaler with me, otherwise I probably would have panicked. That scared me and kept me from walking for a couple of weeks. I talked to my doctor about it, and $Gender said to take it slow and give it another shot. I tried again, and thankfully I didn't have any big problems. After a couple of weeks, it was much easier to head out on a walk without worrying so much. | 4440 |
| Text | "Osteo" in HealthHistory and not Barrier1=="BarNoOne" | Sometimes it was tough to keep going because I was worried about my osteoporosis. I didn't really have a great place to walk where I felt comfortable and knew I wouldn't fall. But when a friend told me to try the local high school track because it was flat, that seemed to make things easier. The more I walked there, the more comfortable I got with it. During the week, I also asked friends to join me, both to make sure I didn't fall and to keep me company. | 4450 |
| Text | "Osteo" in HealthHistory | Sometimes it was tough to keep going because I was worried about my osteoporosis. I didn't really have a great place to walk where I felt comfortable and knew I wouldn't fall. But when a friend told me to try the local high school track because it was flat, that seemed to make things easier. The more I walked there, the more comfortable I got with it. | 4460 |
| Text | "HBP" in HealthHistory | Sometimes it was tough to keep going because I was worried about my high blood pressure. One time I went in for a check-up, and it was pretty high. That really scared me, and I sure didn't feel like going for a walk and risking it getting worse. I took a break for a couple of weeks and got myself one of those blood pressure gadgets so I could check it at home. Once I was comfortable again, I gave it a shot. Things have been going pretty well ever since. | 4480 |
| Text |  | Sometimes it was tough to keep going, especially the time I got the flu. I felt awful for 2 whole weeks, and it threw me off my walking routine. Once I started feeling better, I didn't really feel like getting out and walking again because it felt like I was starting all over again. After a week of moping around the house, I decided to give it another shot. I got back to my routine sooner than I thought, and things have been going pretty well ever since. | 4490 |
| EndSelect |  |  | 4500 |
| EndBlock |  |  | 4510 |
| Block | Barrier2=="BarEffort" or Barrier3=="BarEffort" |  | 4540 |
| Text |  | After awhile, walking around the neighborhood got a little boring for me. I had a hard time increasing my number of steps without going out on longer walks, which I really didn't feel like doing. That's why I was surprised to see such a high step count one day because I hadn't gone for a long walk. I realized the steps must have come from | 4550 |
| Select | 1 |  | 4560 |
| Text | LawnWork=="Yes" | being outside raking all the leaves that had been covering our yard. I couldn't believe that I could get that many steps in just by doing work around the yard. | 4570 |
| Text | HomeRepairs=="Yes" | the time I spent painting the trim on the house. I had no idea I could get that many steps in just by doing some work around the house. | 4580 |
| Text | LtHousework=="Yes" or HeavyHousework=="Yes" | the time I spent doing spring cleaning. It had rained for most of the week, and I decided to get some much needed cleaning done inside the house. I had no idea I could get that many steps in just by doing some work around the house. | 4590 |
| Text |  | the time I spent cleaning up the house before a family get-together. I had no idea I could get that many steps in just by doing a little cleaning around the house. | 4600 |
| EndSelect |  |  | 4610 |
| EndBlock |  |  | 4620 |
| Block | Barrier2=="BarEnergy" or Barrier3=="BarEnergy" |  | 4521 |
| Text |  | Sometimes it was tough to keep going, especially when I didn't really feel like I had the energy to get out and walk enough to make it worthwhile. I started a bad trend of not going out at all when that happened. I knew I needed to make a change, so after that, I made an effort to walk at least 2 times in the next week. Somehow, I was able to do it, and I was surprised to feel like I had more energy than before. I think it helped that I decided to walk in the morning. I usually have more energy then anyway. | 4630 |
| EndBlock |  |  | 4631 |
| Block | Barrier2=="BarHurt" or Barrier3=="BarHurt" |  | 4632 |
| Text |  | Sometimes it was tough to keep going. One morning I was out on a walk, and I got distracted by a biker passing by and ended up tripping on a crack in the sidewalk and falling down. I didn't really hurt myself, but I sure could have, and that scared me. I didn't feel like going out on a walk after that for awhile. After a week or so, I decided to give it another shot, and made sure to be more cautious of where I was walking. Thankfully, I haven't had any falls since then, and things have gone pretty well. | 4640 |
| EndBlock |  |  | 4641 |
| Block | Barrier2=="BarBadMood" or Barrier3=="BarBadMood" |  | 4642 |
| Text |  | Sometimes it was tough to keep going, especially when I wasn't in the mood to get out and go for a walk. Exercise would be one of the last things I felt like doing on some of those bad days. Even though it was tough to do, I knew that getting out and being active was better than moping around the house. When I got into a walking routine, things didn't always get better, but I can say this, they never got worse. | 4650 |
| EndBlock |  |  | 4651 |
| Block | Barrier2=="BarExpensive" or Barrier3=="BarExpensive" |  | 4652 |
| Text |  | Sometimes it was tough to keep going. When I started out, I got a gym membership thinking that it would motivate me to exercise because I was paying for it. I ended up not liking the gym very much, and it was expensive, so I got stuck in a rut of not exercising. I didn't want to give up, so I tried walking some outdoors, and it was so much nicer than being inside that gym. I got into a routine, and things have been going pretty well ever since. | 4660 |
| EndBlock |  |  | 4661 |
| Block | Barrier2=="BarSafePlace" or Barrier3=="BarSafePlace" |  | 4670 |
| Text |  | Sometimes it was tough to keep going because | 4680 |
| Select | 1 |  | 4690 |
| Text | SafeWalkNight>=1 and SafeWalkNight<=2 and SafeWalkDay>=1 and SafeWalk<=2 | my neighborhood isn't really the safest place to take a walk during the day, and definitely not at night. | 4700 |
| Text | SafeWalkNight>=1 and SafeWalkNight<=2 | my neighborhood isn't really the safest place to take a walk, especially at night. | 4710 |
| Text | SafeWalkDay>=1 and SafeWalkDay<=2 | my neighborhood isn't really the safest place to take a walk during the day. | 4720 |
| Text | SidewalksSafe>=1 and SidewalksSafe<=2 | I didn't feel comfortable walking on the sidewalks and paths around my neighborhood. They're really poorly taken care of. | 4730 |
| Text |  | I didn't really feel that I had a safe place to exercise. | 4740 |
| EndSelect |  |  | 4770 |
| Text |  | I had to think of creative ways to increase my steps. On some days I would go to the local mall to walk around. Other times, I just found ways to be active | 4780 |
| Select | 1 |  | 4790 |
| Text | containsOne(Employment, ["FullTime", "PartTime"]) | at work by taking some extra trips to the water fountain for a drink and parking my car further away from the office. I even stopped sending as many emails as I used to and just walked down to my co-workers offices to ask them a question. | 4800 |
| Text |  | by doing a little extra work around the house. Even though I didn't get as many steps in while I was outside compared to walking, I was doing something. My house has sure benefited though. The yard looks great and I can actually get in the garage again. | 4810 |
| EndSelect |  |  | 4820 |
| EndBlock |  |  | 4830 |
| Block | Barrier2=="BarSweat" or Barrier3=="BarSweat" |  | 4831 |
| Text |  | Sometimes it was tough to keep going, especially when the warmer weather came along and the sweating began. I hate sweating, so that kept me indoors for awhile when summer hit. I was falling short of my goal, so I knew I had to change my routine. I decided to move my walking indoors to the mall for the hotter days so I didn't have to worry about sweating much. | 4840 |
| EndBlock |  |  | 4841 |
| Block | (Barrier2=="BarNoOne" or Barrier3=="BarNoOne") and not WantSupport=="No" |  | 4850 |
| Text |  | Yeah, like I said, it got tough after awhile, especially during those times when I needed help to get moving and I didn't have anyone to walk with. | 4860 |
| Select | 1 |  | 4870 |
| Text | "Spouse" in WhoSupport and Gender=="Male" and MaritalStatus=="Married" | When I talked to my wife about how I was struggling with the program, she actually offered to join me on some of my walks. It was just what I needed. | 4880 |
| Text | "Spouse" in WhoSupport and Gender=="Female" and MaritalStatus=="Married" | When I talked to my husband about how I was struggling with the program, he actually offered to join me on some of my walks. It was just what I needed. | 4890 |
| Text | "Spouse" in WhoSupport and MaritalStatus=="Partner" | My partner recognized I was struggling to keep up with the program, and actually offered to join me on some of my walks. It was just what I needed. | 4900 |
| Text | WantSupport=="Yes" and "Friend" in WhoSupport | When I talked to my friends about how I was struggling with the program, they actually offered to join me on some of my walks. It was just what I needed. | 4910 |
| Text | Employment in ("FullTime","PartTime") and "Coworker" in WhoSupport | When I talked to some co-workers and I mentioned something about the program, they actually offered to join me on some of my walks. We agreed to fit in a walk at lunch, which turned out to be a great way to break up the day. | 4920 |
| Text |  | When I talked to some neighbors about how I was struggling with the program, they actually offered to join me on some of my walks. It was just what I needed. | 4930 |
| EndSelect |  |  | 4940 |
| Text |  | It made it a little easier having someone there to talk to as I walked. | 4950 |
| EndBlock |  |  | 4960 |
| Block | (Barrier2=="BarNoOne" or Barrier3=="BarNoOne") and WantSupport=="No" |  | 4961 |
| Text |  | Like I said, I didn't think I'd need the help, but things got tough and I wasn't keeping up. One day I was chatting with some neighbors about how I was struggling with the program and they actually offered to join me on some of my walks. It made it a little easier having someone there to talk to as I walked. | 4970 |
| EndBlock |  |  | 4971 |
| Block | Barrier2=="BarSelfConscious" or Barrier3=="BarSelfConscious" |  | 4972 |
| Text |  | Sometimes it was tough to keep going because I didn't really like exercising in public. For awhile I tried finding places to walk where no one was around. I did find a few park trails where no one usually was, but that seemed to get busier soon after I discovered it. I got tired of looking and realized that what I was doing for myself was more important than what others thought about me. The funny thing is once I began to see that, I didn't really notice the trails being so busy. Maybe it was always just in my head! | 4980 |
| EndBlock |  |  | 4981 |
| EndSelect |  |  | 4990 |
| Paragraph |  |  | 4991 |
| Text |  | **Do you have any advice for someone in a walking program?** | 5000 |
| Paragraph |  |  | 5001 |
| Select | 1 |  | 5010 |
| Text | CADSev=="High" or DIABSev=="High" or BMI>=30 | It's easy to try and push too hard and do too much at one time. That's what happened to me. I tried getting in too many steps on a walk because I was trying to make up for not exercising earlier in the week. It was a bad idea. I had to take a few days off after that. | 5020 |
| Text | "Golf" in EnjoyRecAct | Don't just think about going for walks around the neighborhood as your only way to be active. I love that I can combine walking with golf. Changing the scenery is key. I even sometimes drive to this park with trails. It's all about enjoying my surroundings while I'm out there. | 5030 |
| Text | "Tennis" in EnjoyRecAct | Don't just think about going for walks around the neighborhood as your only way to be active. Adding variety to the routine is key. Tennis was always one of my favorite sports, but I hadn't played much in a while. I decided to get back into it by calling up some friends and playing doubles a couple of times a week. | 5040 |
| Text | "Bike" in EnjoyRecAct | Don't just think about going for walks around the neighborhood as your only way to be active. Take a bike out for a spin. I've even taken a ride to a park and done some walking on trails there. It's a great way to find new places to walk. Changing the scenery is key. It's all about enjoying my surroundings while I'm out there. | 5050 |
| Text | "Bowl" in EnjoyRecAct | Don't just think about going for walks around the neighborhood as your only way to be active. Adding variety to the routine is key. I got back into the bowling league that I used to be a part of a few years ago. It's great to know I can do what I love and get steps in at the same time. | 5060 |
| Text | "Garden" in EnjoyRecAct | Don't just think about going for walks around the neighborhood as your only way to be active. I love that I can be active in my garden. This year I really wanted to make the yard look nice, and it paid off in more ways than one. I was getting some more steps in and the yard looks great! | 5070 |
| Text | CurrentSupport=="Yes" | Some days will be harder than others. Make sure to welcome the support from people around you. It's not easy to keep walking all the time, but if you have someone there who can give you a boost, it makes it a lot easier. | 5080 |
| Text | WorkPayVol=="Yes" | Don't just think about going for walks around the neighborhood as your only way to be active. I get some walking in when I volunteer at the museum giving tours. I love that I can stay busy doing something that I care about and be exercising at the same time. It also helps to have a change of scenery. I like to enjoy my surroundings when I'm walking, that way I don't get bored. | 5090 |
| Text | OwnDog=="Yes" | Don't just think about going for walks around the neighborhood as your only way to be active. I love taking my dog to the park and walking the trails. He seems to love it too. Changing the scenery is key. It's all about enjoying my surroundings while I'm out there. | 5100 |
| Text |  | It's easy to try and push too hard and do too much at one time. That's what happened to me. I tried getting in too many steps on a walk because I was trying to make up for not exercising earlier in the week. It was a bad idea. I had to take a few days off after that. | 5105 |
| EndSelect |  |  | 5106 |
| Paragraph |  |  | 5110 |
| Text |  | **What to Expect Next** | 5115 |
| Paragraph |  |  | 5120 |
| Text |  | We hope that your **Stepping Up To Health** personalized sessions have been helpful in your efforts to increase your daily steps and take better care of your body. As you continue beyond **Stepping Up To Health**, try to maintain any progress that you've made while setting new goals to include more steps in your day. Regular exercise is not only important for this program; it is something that has hopefully become part of your regular routine. | 5130 |
| Paragraph |  |  | 5140 |
| Text |  | We realize that sometimes you may find it hard to reach your daily goals. Ups and downs are part of life. The important thing is that you bounce back to resume your healthier walking habits as soon as you can. Remember, walking for life is not a sprint -- it's a long-distance race. It's not how quickly or dramatically you change your lifestyle, but whether you have the stamina to maintain healthy exercise habits for the long haul. | 5150 |
| Paragraph |  |  | 5160 |
| Text |  | As you continue on, remind yourself now and again about why you've changed your walking habits. What has motivated you to increase your daily steps? What sources of strength can you draw upon to make these changes long lasting? | 5170 |
| Paragraph |  |  | 5180 |
| Text |  | Since this is the sixth and final **Stepping Up To Health** session, we want to thank you for participating in the program. We also want to encourage you to continue your work to add more steps to your day. Refer back to your previous sessions if you want to review the steps you've already taken. You should also continue to upload your pedometer and check your weekly goals. Congratulations on your success and for making every step count! | 5190 |
