# Supplementary material for: An Online Community Improves Adherence in an Internet-Mediated Walking Program. Part 1: Results of a Randomized Controlled Trial
Source: J Med Internet Res. 2010 Dec 17;12(4):e71. doi: 10.2196/jmir.1338 (PMC3056526; doi:10.2196/jmir.1338)
Supplement: Supplementary file 15 [file jmir_v12i4e71_app15.html]

WG7.html


SUH - Session 7, Last Revision August 8, 2006

|  |  |  |  |
| --- | --- | --- | --- |
| **Command** | **Logic** | **Message** | **Row** |
| Comment |  | Goal of Section: Static page. Will appear after all 6 previous sessions have been released to the user. The page will remain until the program is over. | 10 |
| Section | Page1Header |  | 20 |
| Text | not isEmpty(AddressPref) | **Good Luck $AddressPref!** | 30 |
| Text | isEmpty(AddressPref) | **Good Luck!** | 40 |
| Section | Page1Body |  | 50 |
| Text |  | Thank you for participating in **Stepping Up To Health**. You have received all 6 of your personalized web guides. | 60 |
| Paragraph |  |  | 70 |
| Text |  | Please continue to utilize the **Stepping Up To Health** website. You still have access to:  - New **Daily Tips** that may help you move closer to your daily step goals. - All 6 of your previous personalized **web guides**. - Your walking **progress charts** from throughout the program. - Your weekly goals. - And of course, you should continue to upload your **pedometer**! | 80 |
| Paragraph |  |  | 90 |
| Text |  | Remember, you took the initiative to start this walking program. The longer you keep walking, the more likely it will become a permanent part of your life. We wish you the best of luck as you continue to increase your daily steps and take control of your health. | 100 |
| Paragraph |  |  |  |
| Section | Page1CallOut |  |  |
| Select | 1 |  |  |
| Text | Gender=="Male" | "Obstacles don't have to stop you. If you run into a wall, don't turn around and give up. Figure out how to climb it, go through it, or work around it."  - Michael Jordan |  |
| Text | Gender=="Female" | "Not only are you responsible for your life, but doing the best at this moment puts you in the best place for the next moment."  -Oprah Winfrey |  |
| EndSelect |  |  |  |
