# Supplementary material for: An Online Community Improves Adherence in an Internet-Mediated Walking Program. Part 1: Results of a Randomized Controlled Trial
Source: J Med Internet Res. 2010 Dec 17;12(4):e71. doi: 10.2196/jmir.1338 (PMC3056526; doi:10.2196/jmir.1338)
Supplement: Supplementary file 16 [file jmir_v12i4e71_app16.html]

Barriers.html

or

|  |  |  |  |
| --- | --- | --- | --- |
| **Command** | **Logic** | **Message** | **Row** |
| Block | Barrier == "BarTime" |  | 10 |
| Text |  | **MY DAY IS BUSY. I JUST DON'T HAVE TIME TO WALK.** | 20 |
| Paragraph |  |  | 30 |
| Text |  | You're busy. We understand. We know that sometimes it's difficult to find time to fit physical activity into your schedule. Work, family, and social commitments leave very little time to do much else. But there's always a way to add some extra steps into your day, even if it is only for 10 minutes. Here are some ideas for how to make some extra time to walk: | 40 |
| Select | 1 |  | 50 |
| Text | HomeStatus=="No" and containsOne (AdultInHome, ["Spouse", "Partner", "AdultChild", "ImFamily", "Relative"]) | - Take a short walk after dinner and invite your **family** to join you. It's a great time to catch up on each other's days. | 60 |
| Text | HomeStatus=="No" and containsOne (AdultInHome, ["OthAdult", "Friend"]) | - Take a short walk after dinner and invite your **housemates** to join you. It's a great time to catch up on each other's days. | 70 |
| Text |  | - Take a short walk after dinner and invite a **friend or neighbor** to join you. It's a great time to catch up on each other's days. | 80 |
| EndSelect |  |  | 90 |
| Text | containsOne (Employment, ["FullTime", "PartTime"]) | - **Make the most of your commuting time**. Walk or bike to work. If you ride the bus to and from work, get off a few blocks early and walk the rest of the way. If you drive to work, park far enough from the building entrace so it takes you at least 5 minutes to get to it. | 100 |
| Select | 1 |  | 110 |
| Text | containsOne (Employment, ["FullTime", "PartTime"]) | - **Sneak in small walking trips** during a busy day. Instead of emailing or calling someone in your office, walk over to their desk to talk with them. Another option is to finish your lunch with a 10 minute walk. | 120 |
| Text | containsOne (Employment, ["UnLooking", "UnNotLooking", "Homemaker", "Retired", "Disabled"]) or isEmpty(Employment) | - **Sneak in small walking trips** during a busy day. How? When at home, walk around the yard or around the block while dinner is in the oven. Just be sure to check your watch so you know how long you have to walk. | 130 |
| Text | containsOne (Employment, ["Student"]) | - **Sneak in small walking trips** during a busy day. On your way to class, park your car further away from where your class meets. Or, get off the bus a few stops earlier and walk the rest of the way. | 140 |
| EndSelect |  |  | 150 |
| Select | 1 |  | 160 |
| Text | containsOne (Employment, ["FullTime", "PartTime"]) | - Schedule your time to walk as if it were an **important meeting** with a manager at work. Write it into your planner so that it becomes part of your daily routine. Invest your time and effort into your health by making it a priority. | 170 |
| Text | containsOne (Employment, ["UnLooking", "UnNotLooking", "Homemaker", "Retired", "Disabled"]) or isEmpty(Employment) | - Schedule your time to walk as if it were an **important appointment**. Write it into your planner so that it becomes part of your daily routine. Invest your time and effort into your health by making it a priority. | 180 |
| Text | containsOne (Employment, ["Student"]) | - Schedule your time to walk as if it were an **important class**. Write it into your planner so that it becomes part of your daily routine. Invest your time and effort into your health by making it a priority. | 190 |
| EndSelect |  |  | 200 |
| Select | 4 |  | 210 |
| Text | StoresWalkDist>=3 | - **Work walking into your daily errands**. Instead of driving to a store, walk to the closest market or convenience store. Or if you have to drive, pick a parking spot at the far end of the lot. | 220 |
| Text | Age>1 and Age<50 | - **Wake up early**. Try getting up 30 minutes earlier than you normally do and use the extra time to walk. Some research suggests that people who exercise in the morning are more likely than are others to stick with it over the long term. | 221 |
| Text | Age>=50 | - **Wake up early**. Try getting up 30 minutes earlier than you normally do and use the extra time to walk. Some research suggests that people who exercise in the morning are more likely than are others to stick with it over the long term. If you're too stiff in the morning, however, wait to walk until later in the day. | 222 |
| Text |  | - **Plan ahead.** Commit to a 15 minute walk each day - and stick to it! Put it on your calendar, in your day planner, or set your alarm clock. If it's at the beginning of the day, think through what needs to get done that day. If it's in the evening, use it as a way to unwind. | 230 |
| Text |  | - When given the chance, use the **stairs instead of an elevator** as a way to increase your daily steps. | 240 |
| Text |  | - **Turn inactive time into active time**. Time spent at home doesn't have to be "couch potato" time. Cut your TV time in half and turn it into walking time. | 250 |
| EndSelect |  |  | 270 |
| EndBlock |  |  | 280 |
| Block | Barrier == "BarHealthProb" |  | 290 |
| Text |  | **SOME OF MY HEALTH ISSUES MAKE IT DIFFICULT FOR ME TO WALK.** | 300 |
| Paragraph |  |  | 310 |
| Text |  | It can be hard to stick to a walking program when you have health problems that may get in the way of walking as much as you'd like. We understand that it can be difficult. Here are some facts that might make you feel better about walking as you manage your condition. | 320 |
| Select | 1 |  | 330 |
| Text | CADExAfraid=="Yes" and CADSev in ("High", "Low") | - Having **heart disease** may make it difficult to walk. And it might be hard for you to stick to a walking program when you're afraid to exercise because of your condition. Walk slowly and listen to your body. Walk with a friend until you feel a little more comfortable being on your own. | 340 |
| Text | CADSev in ("High", "Low") | - Having **heart disease** may make it difficult to walk. Walk slowly and listen to your body. Take breaks when you need to and rest when necessary. | 350 |
| EndSelect |  |  | 360 |
| Text | CADSwell>=3 | - Heart problems **can cause swelling** in your ankles or legs. Since this has been a problem for you in the past, you want to make sure you take good care of your legs before and after walks. If you notice swelling, lay down in bed or on a couch and elevate your legs above your heart level. This will help reduce swelling. | 370 |
| Text | DIABSev in ("High", "Low") | - Managing **diabetes** is not an easy task. But walking can help maintain your blood sugar level and control your cholesterol. Take good care of your feet. Wear comfortable shoes and socks so that you don't get blisters. | 380 |
| Text | BMI>=25 | - As someone who is **overweight**, you may experience being out of breath as you begin to walk. That's to be expected. It may make it difficult in the beginning, but as you walk more often, you may experience that your breathing comes a little easier. Walk slowly and walk with walking sticks for additional balance if needed. | 390 |
| Select | 1 |  | 400 |
| Text | "Arthritis" in HealthHistory | - The pain and stiffness that comes with **arthritis** can make it difficult to walk, especially when you are starting out. Use a stationary bike to ease the pressure on your joints. Staying active may help strengthen the muscles in your joints and reduce the pain and stiffness so exercise can be easier in the future. | 410 |
| Text | "Asthma" in HealthHistory | - Because of your **asthma,** you may be afraid to start walking. Learn how your body and lungs react to your walks and then begin to increase your steps and speed. And always remember to carry your inhaler. | 420 |
| Text | "Osteo" in HealthHistory | - Since you have been diagnosed with **osteoporosis**, you want to pick safe, flat services to walk. Walk on a treadmill, on a track, or on level sidewalks to prevent getting hurt. | 430 |
| Text | "HBP" in HealthHistory | - Are you worried about your **high blood pressure**? You can personally see how walking can help lower your numbers. Check your pressure now. Every few weeks, check it again. What are you seeing with your numbers? Are they going down? Monitor your changes. | 440 |
| Text | "HiChol" in HealthHistory and not "HBP" in HealthHistory | - Are you worried about your **high cholesterol**? You can personally see how walking can help lower your numbers. Have your cholesterol level checked. Every few months, check it again. What are you seeing with your numbers? Are they going down? Monitor your changes. | 450 |
| EndSelect |  |  | 460 |
| Select | 1 |  | 470 |
| Text | SF36OneFlight=="SigLimit" and not SF36SevFlights in ("SomeLimit", "SigLimit") | - Climbing one flight of stairs is very difficult for you. **Pick walking activities that don't require stairs**. For example, while at the mall, take the escalator or elevator to get to your desired floor, then continue walking through the stores. | 480 |
| Text | SF36OneFlight=="SomeLimit" | - Climbing one flight of stairs can be difficult for you. **Pick walking activities that don't require stairs**. For example, while at the mall, take the escalator or elevator to get to your desired floor, then continue walking through the stores. | 490 |
| Text | SF36SevFlights=="SigLimit" | - Climbing several flights of stairs is very difficult for you. **Pick walking activities that don't require stairs**. For example, while at the mall, take the escalator or elevator to get to your desired floor, then continue walking through the stores. | 500 |
| Text | SF36SevFlights=="SomeLimit" | - Climbing several flights of stairs can be difficult for you. **Pick walking activities that don't require stairs**. For example, while at the mall, take the escalator or elevator to get to your desired floor, then continue walking through the stores. | 510 |
| Text | SF36LowIntAct=="SigLimit" | - Your health significantly limits your ability to do low intensity activities, such as playing golf, home maintenance, or gardening. To get in your daily steps, **add them to activities you already do** on a daily basis. Walk a few extra aisles at the grocery or department store, walk in place during television commercials and go for short walks around the block after dinner. | 520 |
| Text | SF36LowIntAct=="SomeLimit" | - Your health somewhat limits your ability to do low intensity activities, such as playing golf, home maintenance, or gardening. To get in your daily steps, **add them to activities you already do** on a daily basis. Walk a few extra aisles at the grocery or department store, walk in place during television commercials and go for short walks around the block after dinner. | 530 |
| Text | SF36dIntAct=="SigLimit" | - Your health significantly limits your ability to do moderate intensity activities, such as golfing, bicycling, pushing a vacuum cleaner, and fast walking. **Stick to low intensity activities,** such as casual walking, home maintenance, or gardening. | 540 |
| Text | SF36dIntAct=="SomeLimit" | - Your health somewhat limits your ability to do moderate intensity activites, such as playing golf, bicycling, pushing a vacuum cleaner, and fast walking. **Stick to low intensity activities**, such as casual walking, home maintenance, or gardening. | 550 |
| EndSelect |  |  | 560 |
| Text |  | - Always carry a **cell phone** while you walk. | 570 |
| Text |  | - **Tell** a friend or family member where you're going before setting off. | 580 |
| Text |  | - If your health continues to make it difficult to walk, schedule an appointment with your doctor. | 590 |
| EndBlock |  |  | 600 |
| Block | Barrier == "BarTired" |  | 610 |
| Text |  | **I'M TOO TIRED TO WALK.** | 620 |
| Paragraph |  |  | 630 |
| Text |  | It can be hard to stick to your walking plan when you're feeling tired. The tips below can help you get out the door even when you feel like you don't have the energy to walk. | 640 |
| Text | WantSupport=="Yes" | - **Ask** a friend or family member to walk with you. They can encourage you and help boost your energy when you are feeling tired. | 650 |
| Select | 1 |  | 660 |
| Text | containsOne(Employment, ["FullTime", "PartTime"]) | - Keep a pair of **walking shoes** at your workplace so that you're ready when you find a few minutes to walk when you're not feeling so tired. | 670 |
| Text |  | - Keep a pair of **walking shoes** in your car so that you're ready when you find a few minutes to walk when you're not feeling so tired. | 680 |
| EndSelect |  |  | 690 |
| Text |  | - **Make plans to walk** and stick to them. Every night after dinner, schedule a walk. Once you start doing it every evening, it will become part of your daily routine and you might begin to head out the door without thinking about it. | 700 |
| Text |  | - Walking can actually be a great way to **feel less tired during the day**. Add in more steps during the day while doing your regular daily activities. Take the stairs more often, walk around the block after meals, walk up and down all the aisles in the grocery store, and add in extra steps while you're cleaning. After a few days, you might begin to notice that you feel less tired during the day. | 710 |
| Text |  | - **Drink more water**. Water, the most abundant nutrient in the human body, is often a forgotten nutrient. Drink water before, after and during your walks. Being fatigued may be a sign of dehydration and lack of water can make you even more tired. | 720 |
| Text |  | - **Plan walking tours of cities** when you travel. Most people feel energized when visiting new spots. | 730 |
| Text |  | - **When shopping**, park in the far back corner of the parking lot and walk further to the door. As an extra bonus -- you're more likely to find spots without other cars parked alongside, which reduces the chance of getting scratches and dings. | 740 |
| EndBlock |  |  | 750 |
| Block | Barrier == "BarEnergy" |  | 930 |
| Text |  | **I DON'T HAVE ENOUGH ENERGY TO WALK.** | 940 |
| Paragraph |  |  | 950 |
| Text |  | You may not have the energy now, but as a result of walking you'll begin to feel more energized during the day. Here are some tips to get you into your walking shoes and out the door even when you feel like your energy level is low: | 960 |
| Text | containsOne (Employment, ["FullTime", "PartTime"]) | - Use a restroom, copy machine, or water fountain that is further from your work area. This will help you get in some **extra steps** without having to put in a lot of effort. | 970 |
| Select | 1 |  | 980 |
| Text | "AmIndian" in Race | - **Embrace your roots** and find the energy to reach your daily step goal. Celebrate your American Indian heritage by learning a traditional dance. | 990 |
| Text | "Asian" in Race | - **Embrace your roots** and find the energy to reach your daily step goal. Celebrate your Asian heritage by learning a traditional dance. | 1000 |
| Text | "Black" in Race | - **Embrace your roots** and find the energy to reach your daily step goal. Celebrate your African-American heritage by learning a traditional dance. | 1010 |
| Text | "PacIsland" in Race | - **Embrace your roots** and find the energy to reach your daily step goal. Celebrate your Pacific Island heritage by learning a traditional dance. | 1020 |
| Text | "White" in Race | - **Embrace your roots** and find the energy to reach your daily step goal. Celebrate your cultural heritage by learning a traditional dance. | 1030 |
| Text |  | - **Embrace your roots** and find the energy to reach your daily step goal. Celebrate your cultural heritage by learning a traditional dance. | 1040 |
| EndSelect |  |  | 1050 |
| Text | OwnDog=="Yes" | - Think of **all the energy** your dog has. Walking in the park may revitalize you while also allowing your dog to stretch his legs. | 1060 |
| Text |  | - When do you feel you're **most energetic**? In the morning, mid-day or evening? Plan your walks around your energy level. | 1070 |
| Text |  | - Need a small way to add in steps when you feel low on energy? When **on the phone,** stand up and walk around as you talk. | 1080 |
| Text |  | - **Do something new and different.** Walk a different path through the park, or walk in a different neighbood. Visit a mall in a neighboring town. By spicing up your routine, you may find that you actually look forward to your walks. | 1090 |
| Text |  | - **Challenge yourself.** Set up a friendly competition with a friend. Whoever walks the most steps before a certain date gets taken out to lunch by the other. | 1100 |
| EndBlock |  |  | 1110 |
| Block | Barrier == "BarHurt" |  | 1120 |
| Text |  | **I DON'T WANT TO GET HURT.** | 1130 |
| Paragraph |  |  | 1140 |
| Text |  | Fear of getting hurt keeps a lot of people from beginning a walking program. No one wants to slip and fall, sprain an ankle, or pull a muscle. However, if you take the proper precautions, your risk of getting hurt is very small. | 1150 |
| Select | 1 |  | 1160 |
| Text | WalkDog=="Yes" | - **Add steps to activities that you already do**. Trade in your riding mower for a push mower, rake your leaves more often, walk your dog a little further, wash and wax your car yourself. | 1170 |
| Text |  | - **Add steps to activities that you already do**. Trade in your riding mower for a push mower, rake your leaves more often, wash and wax your car yourself. | 1180 |
| EndSelect |  |  | 1190 |
| Text | Age>=50 | - When you're done walking, **stretch your muscles**. This will help prevent pain the following day and also help increase your flexibility and range of motion. Maintaining the full range of motion through your joints will help your balance. Coordination and balance will help keep you mobile and less likely to fall. | 1191 |
| Text | Age>1 and Age<50 | - When you're done walking, **stretch your muscles**. This will help prevent pain the following day and also help increase your flexibility and range of motion. | 1192 |
| Text |  | - Walking won't cause you to get hurt **if you're careful**. Begin your walk slowly to warm up. Count your steps per minute. After 5 minutes, increase your steps per minute by at least 15 steps for the rest of your walk. | 1200 |
| Text |  | - **Wear appropriate shoes and socks**. Make sure your shoes fit properly and that they have good traction. | 1210 |
| Text |  | - **Bring a water bottle**. Drink plenty of water before, during, and after your walks to stay hydrated. You'll be less likely to suffer from dehydration. | 1230 |
| Text |  | - **Walk with a partner**. Even though your chance of getting hurt is very small, you may feel more comfortable knowing that you'll have someone there who can help you if necessary. | 1240 |
| EndBlock |  |  | 1250 |
| Block | Barrier == "BarEffort" |  | 1520 |
| Text |  | **WALKING TAKES TOO MUCH EFFORT.** | 1530 |
| Paragraph |  |  | 1540 |
| Text |  | Being active does not mean having to get sweaty and out-of-breath. Walking can be enjoyable and peaceful rather than hard work. Follow these tips to help keep your effort at a level you're comfortable with. | 1550 |
| Select | 1 |  | 1560 |
| Text | CurrentSupport=="Yes" and "Spouse" in WhoSupport and Gender=="Male" | - You told us that you are **getting support** to walk from your wife. Ask her to walk with you. Walking won't seem like so much effort if you have someone with you whose company you enjoy. You may be surprised by how quickly the time flies by. | 1570 |
| Text | CurrentSupport=="Yes" and "Spouse" in WhoSupport and Gender=="Female" | - You told us that you are **getting support** to walk from your husband. Ask him to walk with you. Walking won't seem like so much effort if you have someone with you whose company you enjoy. You may be surprised by how quickly the time flies by. | 1580 |
| Text | CurrentSupport=="Yes" and "Sister" in WhoSupport | - You told us that you are **getting support** to walk from your sister. Ask her to walk with you. Walking won't seem like so much effort if you have someone with you whose company you enjoy. You may be surprised by how quickly the time flies by. | 1590 |
| Text | CurrentSupport=="Yes" and "Brother" in WhoSupport | - You told us that you are **getting support** to walk from your brother. Ask him to walk with you. Walking won't seem like so much effort if you have someone with you whose company you enjoy. You may be surprised by how quickly the time flies by. | 1600 |
| Text | CurrentSupport=="Yes" and "Child" in WhoSupport | - You told us that you are **getting support** to walk from your child(ren). Ask them to walk with you. Walking won't seem like so much effort if you have someone with you whose company you enjoy. You may be surprised by how quickly the time flies by. | 1610 |
| Text | CurrentSupport=="Yes" and "Mom" in WhoSupport | - You told us that you are **getting support** to walk from your mom. Ask her to walk with you. Walking won't seem like so much effort if you have someone with you whose company you enjoy. You may be surprised by how quickly the time flies by. | 1620 |
| Text | CurrentSupport=="Yes" and "Dad" in WhoSupport | - You told us that you are **getting support** to walk from your dad. Ask him to walk with you. Walking won't seem like so much effort if you have someone with you whose company you enjoy. You may be surprised by how quickly the time flies by. | 1630 |
| Text | CurrentSupport=="Yes" and "Friend" in WhoSupport | - You told us that you are **getting support** to walk from a friend. Ask them to walk with you. Walking won't seem like so much effort if you have someone with you whose company you enjoy. You may be surprised by how quickly the time flies by. | 1640 |
| Text | CurrentSupport=="Yes" and "Coworker" in WhoSupport | - You told us that you are **getting support** to walk from your coworker. Ask them to walk with you. Walking won't seem like so much effort if you have someone with you whose company you enjoy. You may be surprised by how quickly the time flies by. | 1650 |
| Text | CurrentSupport=="No" and WantSupport=="Yes" | - You shared with us that you're not currently getting support to walk from those around you. Have you tried asking someone to walk with you? Who do you think might want to join you? Walking may not seem like so much effort if you have someone with you whose **company you enjoy**. You may be surprised by how quickly the time flies by. | 1660 |
| Text | CurrentSupport=="No" and WantSupport=="No" | - You may be surprised by how quickly the time flies by if you're walking with someone whose **company you enjoy**. Think about asking someone to walk with you the next time you go out -- walking may not seem like so much effort. | 1670 |
| Text |  | - Walking may not seem like so much effort if you have someone with you whose company you enjoy. **Ask someone to walk with you**. You'll be amazed by how quickly the time flies by. | 1680 |
| EndSelect |  |  | 1690 |
| Text | Gender=="Male" | - Find a **fun activity** that you enjoy that will help increase your daily steps. How about a few games of miniature golf? | 1700 |
| Text | Gender=="Female" | - Find a **fun activity** that you enjoy that will help increase your daily steps. Visit some local craft shows or art fairs. | 1710 |
| Text | StreetsHillyDiff>=3 | - You told us that it's difficult to walk in your neighborhood **because of the hills.** Try finding a place to walk indoors at a local gym or mall. Look for a local park with flat trails if you prefer to be outdoors. And don't forget -- it's always possible to get in extra steps around the house. | 1720 |
| Select | 1 |  | 1730 |
| Text | containsOne (AdultInHome, ["Spouse", "Partner", "AdultChild", "ImFamily", "Relative"]) | - **Use variety** to "jump start" your walking plan. Add 10 minute walks after every meal. Add some walking time into your morning routine, or make evening walks a family event. | 1740 |
| Text |  | - **Use variety** to "jump start" your walking plan. Add 10 minute walks after every meal. Add some walking time into your morning routine, or make evening walks a nightly event with a friend. | 1750 |
| EndSelect |  |  | 1760 |
| Text |  | - **Pace yourself**. If you go at it full force, you'll burn out. Or, you'll hurt yourself, get discouraged, and quit. Prepare yourself for the long haul. Start out gradually, then build up by adding small challenges as you go along. | 1770 |
| Text |  | - A daily walk can be a great time for you to **focus on yourself** and think about your life. During your next walk, think about what's important to you. Many people say that their family and friends are most important. When thinking about the people that you value, and how walking may help you live a longer and healthier life, you may find that it's worth the effort. | 1780 |
| Text |  | - **Add in extra steps** during tasks you already have to do. For example, consider not using the drive through. Park and walk in to the bank or restaurant instead. Think of small things you can do to increase your steps without putting in much effort. | 1790 |
| Text |  | - **Convenience is the key**. Add steps to the activities that you already do, such as house work and shopping. | 1800 |
| EndBlock |  |  | 1810 |
| Block | Barrier == "BarBadMood" |  | 1820 |
| Text |  | **WHEN I'M IN A BAD MOOD, I DON'T WANT TO WALK.** | 1830 |
| Paragraph |  |  | 1840 |
| Text |  | It can be hard to motivate yourself to exercise when you're in a bad mood. But did you know that walking can actually help improve your mood? Walking has benefits beyond improving your fitness. Many people walk as much for mental and spiritual well-being as for fitness. If you are feeling down, let your feet do the walking to a better outlook. | 1850 |
| Text | 1 <=DownheartedBlue <=3 or "MdDisorder" in HealthHistory | - Taking a walk can **boost your mood** and feeling of well-being, even for people who occasionally feel downhearted and blue. Walking can be a great stress reliever. During your next walk, think about what makes you feel down. Walking can be a good time to find solutions to things that can make life a bit more difficult at times. | 1860 |
| Select | 1 |  | 1870 |
| Text | containsOne(Employment, ["FullTime", "PartTime"]) and TryingLoseWt=="Yes" | - **De-stress with a quick walk after work**. Walking before dinner may also suppress your appetite, helping to reduce your total daily calorie intake. | 1880 |
| Text | Employment=="Student" and TryingLoseWt=="Yes" | - **De-stress with a quick walk after class**, before heading home for dinner. Walking before eating may also suppress your appetite, helping to reduce your total daily calorie intake. | 1890 |
| EndSelect |  |  | 1900 |
| Text | OwnDog=="Yes" | - Your dog can be a great companion when you're feeling down. Put his leash on and head outside for a long walk. A long walk and the company of your favorite pet can help cheer you up. | 1910 |
| Text |  | - The best **motivation comes from within.** Make yourself a promise to walk and stick to it, even when you're not in the best mood. Just keeping your promise to yourself can make you feel better. | 1920 |
| Text |  | - Exercise causes your body to **produce endorphins**, hormones that are naturally made and released in your body which make you feel good. After walking for a few weeks, compare your current mood to how you felt before you began to walk. You might notice that your walking program may actually help you feel better. | 1930 |
| Text |  | - **Dance** to your favorite music for 10 minutes every day. Dancing is a great way to put you in a better mood and may help you reach your daily step goal. | 1940 |
| Text |  | - **Make it fun**. Listen to music, find a walking buddy, or sign up with a group for a charity walk. | 1950 |
| Text |  | - **Reward yourself.** Make walking fun by treating yourself to a movie or dinner at a nice restaurant when you reach your walking goals. | 1960 |
| EndBlock |  |  | 1970 |
| Block | Barrier == "BarExpensive" |  | 1980 |
| Text |  | **IT'S TOO EXPENSIVE TO JOIN A CLUB OR GYM.** | 1990 |
| Paragraph |  |  | 2000 |
| Text |  | It doesn't have to be expensive to walk. You don't have to join a gym or a club to get a good workout. What are other options? Take a look below. | 2010 |
| Select | 1 |  | 2020 |
| Text | SafeWalkDay>=3 and SafeWalkNight>=3 and SidewalksSafe>=3 | - Take advantage of your **safe neighborhood**. Head outside to the streets or sidewalks, or walk on the local high school's outdoor track. | 2030 |
| Text | (SafeWalkDay>=1 and SafeWalkDay<=2) and (SafeWalkNight>=1 and SafeWalkNight<=2) and (SidewalksSafe>=1 and SidewalksSafe<=2) | - Even though you don't feel safe walking in your own neighborhood, you may be able to find a neighborhood or park in the area where you do feel comfortable. Ask your neighbors and local police for recommendations on **safe locations to walk**. | 2040 |
| Text | SafeWalkDay>=3 and (SafeWalkNight>=1 and SafeWalkNight<=2) | - Take advantage of your **safe neighborhood** during the day. Head outside to the streets or sidewalks, or walk on the local high school's outdoor track. | 2050 |
| Text | (SafeWalkNight>=1 and SafeWalkNight<=2) and isEmpty(SafeWalkDay) | - You don't feel safe walking in your neighborhood at night. That's ok. It's not uncommon for people to feel unsafe at night. **Schedule your walks for times during the day** when you know the sun will light your way. | 2060 |
| Text | (SafeWalkDay>=1 and SafeWalkDay<=2) and SafeWalkNight>=3 | - You feel safe in your neighborhood at night. **Schedule your walks for after dinner and before bed**. Carry a flashlight to help light your way. | 2070 |
| Text |  | - **Find a place to walk where you feel safe**. Neighborhoods, community centers, arboretums, and local shopping areas can all be great, safe choices. | 2080 |
| EndSelect |  |  | 2090 |
| Select | 1 |  | 2100 |
| Text | "Golf" in EnjoyRecAct and Season(BaselineDate, 14)=="Summer" | - You told us that you enjoy **playing golf**. Public courses can be very affordable. You can save even more money by walking instead of using a golf cart. | 2110 |
| Text | "Bowl" in EnjoyRecAct | - You told us that you enjoy **bowling**. Think about how many times you walk back and forth to the lane during one game - up to 20 times! Is this something you feel you could do more often over the week to increase your daily steps? | 2120 |
| Text | "Tennis" in EnjoyRecAct | - You told us that you enjoy playing **tennis**. Think about how many times you cross the court to hit a ball. Play tennis more often to increase your daily steps. Most high schools and community centers have courts that you can use for free. | 2130 |
| Text | "Garden" in EnjoyRecAct and Season(BaselineDate, 14)=="Summer" | - You told us that you enjoy **gardening**. Think about how many times you walk around your yard as you garden. Garden more often to increase your daily steps. And offer to help neighbors and friends with their gardens. Not only will it help reach your daily step goal, you'll improve flexibility in your arms, legs and back. | 2140 |
| Text | "Shuffleboard" in EnjoyRecAct | - You told us that you enjoy **shuffleboard**. Think about how many times you walk around the table or floor board as you play. Is this something you feel you could do more often over the week to increase your daily steps? | 2150 |
| Text | "Bike" in EnjoyRecAct and Season(BaselineDate, 14)=="Summer" | - You told us that you enjoy **bicycling**. How often do you go for a bike ride? Is it time to increase the number of days or the length of time you bike? | 2160 |
| Text | "Bike" in EnjoyRecAct and Season(BaselineDate,14)=="Winter" | - You told us that you enjoy **bicycling**. How often do you go for a bike ride? Now that it's cold out, you may want to think about getting an exercise bike. You might be able to find one at a thrift or secondhand store for a low price. Put it in front of your TV to help reach your daily step goal. | 2170 |
| Text |  | - **Do your favorite activities help you reach your daily step goal**? Bowling, gardening, golfing, bicycling, tennis and many more can be great ways to get in some extra steps without having to go to a gym. | 2180 |
| EndSelect |  |  | 2190 |
| Select | 1 |  | 2200 |
| Text | "Cards" in EnjoyRecAct | - It's great that you enjoy **playing cards**. While playing cards may not be an activity that involves a lot of walking, there are ways to add steps into the activity. Every half hour, put down your cards and take a short walk around the block with the people you're playing cards with. | 2210 |
| Text | "Read" in EnjoyRecAct | - It's great that you enjoy **reading**. While reading may not be an activity that involves a lot of walking, there are ways to add steps into the activity. Every half hour, put down your book and take a short walk around the block. Or, increase your daily steps while wandering the aisles to pick out a new book at your local library or bookstore. | 2220 |
| EndSelect |  |  | 2230 |
| Text | Season(BaselineDate, 14)=="Winter" | - **Take a day trip** to a museum, aquarium or art gallery. It's a great way to increase your steps and vary your routine. | 2240 |
| Text | Season(BaselineDate, 14)=="Summer" | - **Take a day trip** to a museum, aquarium, zoo, or art gallery. It's a great way to increase your steps and vary your routine. | 2250 |
| Text |  | - State and city parks and trails are great places to find some new walking paths. | 2260 |
| Text |  | - **Start a walking group** with some friends, neighbors, or co-workers to make your workout a social event. It's an inexpensive way to get moving while spending time with friends. | 2270 |
| EndBlock |  |  | 2280 |
| Block | Barrier == "BarSafePlace" |  | 2290 |
| Text |  | **I DON'T HAVE A SAFE PLACE TO EXERCISE.** | 2300 |
| Paragraph |  |  | 2310 |
| Text |  | Even when you know the benefits of being active, you may find it very hard to change your lifestyle if you do not have a place to walk where you feel safe. Here are some ideas on how you can feel more comfortable, regardless of where you walk. | 2320 |
| Text | OwnDog=="Yes" | - **Take your dog**! You'll feel safer having a walking companion. | 2330 |
| Text | OwnDog=="No" or isEmpty(OwnDog) | - **Adopt or borrow a dog** to walk with. You'll feel safer having a walking companion. | 2340 |
| Text | Season(BaselineDate, 14)=="Summer" | - In the summer, head to local **fairs and festivals** in your community. You'll be surrounded by people and great sites and smells. | 2350 |
| Text |  | - Many people feel safer about walking when they have a **walking partner**. Ask a friend, neighbor or family member to walk with you. | 2360 |
| Text |  | - When walking outside, carry a cell phone or a whistle. You'll be able to alert someone if you need help. | 2370 |
| Text |  | - You don't always have to leave the **safety of your home** to get in some extra steps. Try walking in place when watching TV. Or, walk around your home when you're on the telephone. Do you have stairs? Walking up and down the stairs during commercials can be a great way to reach your daily step goal. | 2380 |
| Text |  | - **Explore!** Leave your immediate surroundings and see what else is out there. Take your car or the bus to a new neighborhood or park. | 2390 |
| Text |  | - **Many suburbs were built for cars, not walking**. If you live in such an area, get involved with your local government in planning new sidewalks, walking paths and other connectors so you can safely put more walking into your day. | 2400 |
| Text |  | - **Select a walking route** that feels safe to you. A mall, treadmill, indoor track, or local school track used by other walkers and runners can be safer-feeling environments. | 2410 |
| EndBlock |  |  | 2420 |
| Block | Barrier == "BarSweat" |  | 2430 |
| Text |  | **I DON'T LIKE TO SWEAT.** | 2440 |
| Paragraph |  |  | 2450 |
| Text |  | Your body works best when its temperature is about 98.6 degrees F. When your body gets hotter than that, your brain sends a message to your body, telling it to sweat. The sweat leaves your skin through your pores and when it hits the air, the air makes it evaporate. As the sweat evaporates off your skin, you cool down. Even though sweating may make you feel uncomfortable, it's good for your body. Here are some tips to keep you walking even if you don't like to sweat. | 2460 |
| Text | EasyWalkTransit>=3 | - Do you ever **take the bus**? Get off the bus a stop or two before your usual stop and walk the rest of the way. It'll help you increase your steps, and you're not likely to sweat since you aren't specifically out to exercise. | 2470 |
| Text |  | - **Pick a time to walk** when you know that you'll have time to run home and enjoy a cool shower. Sweat might not seem like such a problem if you can freshen up afterwards. | 2480 |
| Text |  | - **Exercise early in the morning or later in the evening** when it's cooler outside. You're less likely to sweat. | 2490 |
| Text |  | - Find an **air-conditioned location**, such as a mall or gym, to walk. | 2500 |
| Text |  | - You don't have to walk at a **high intensity** to reach your daily step goal. It is possible to walk slow, reach your daily step goal, and never break a sweat. | 2510 |
| Text |  | - **Park at the far end** of the parking lot when visiting a store. You will be rewarded by cool air once you've reached the air conditioned store. | 2520 |
| Text |  | - **Hide the tv remote** and walk to the tv to change channels, or return the shopping cart all the way into the store after grocery shopping. Even little things like these will help bring you a little closer to your daily step goal. | 2530 |
| EndBlock |  |  | 2540 |
| Block | Barrier == "BarNoOne" |  | 2550 |
| Text |  | **I DON'T HAVE ANYONE TO EXERCISE WITH.** | 2560 |
| Paragraph |  |  | 2570 |
| Text |  | Friends, family, neighbors, and members of your community may be willing to walk with you. Do you feel comfortable asking someone? Here are some great tips on how you can find a walking buddy: | 2580 |
| Select | 1 |  | 2590 |
| Text | "Spouse" in WhoSupport and Gender=="Female" | - **Invite your husband**. Spending time with a supportive walking partner can be a great way to spend time together without interruption. If he can't walk with you, make a list of people whose company you enjoy and ask someone from the list. | 2600 |
| Text | "Spouse" in WhoSupport and Gender=="Male" | - **Invite your wife**. Spending time with a supportive walking partner can be a great way to spend time together without interruption. If she can't walk with you, make a list of people whose company you enjoy and ask someone from the list. | 2610 |
| Text | "Sister" in WhoSupport | - **Invite your sister.**  Spending time with a supportive walking partner can be a great way to spend time together without interruption. If she can't walk with you, make a list of people whose company you enjoy and ask someone from the list. | 2620 |
| Text | "Brother" in WhoSupport | - **Invite your brother**. Spending time with a supportive walking partner can be a great way to spend time together without interruption. If he can't walk with you, make a list of people whose company you enjoy and ask someone from the list. | 2630 |
| Text | "Child" in WhoSupport | - **Invite your child(ren)**. Spending time with a supportive walking partner can be a great way to spend time together without interruption. If they can't walk with you, make a list of people whose company you enjoy and ask someone from the list. | 2640 |
| Text | "Mom" in WhoSupport | - **Invite your mom**. Spending time with a supportive walking partner can be a great way to spend time together without interruption. If she can't walk with you, make a list of people whose company you enjoy and ask someone from the list. | 2650 |
| Text | "Dad" in WhoSupport | - **Invite your dad**. Spending time with a supportive walking partner can be a great way to spend time together without interruption. If he can't walk with you, make a list of people whose company you enjoy and ask someone from the list. | 2660 |
| Text | "Coworker" in WhoSupport | - **Invite a co-worker**. Spending time with a supportive walking partner can be a great way to spend time together without interruption. If he or she can't walk with you, make a list of people whose company you enjoy and ask someone from the list. | 2670 |
| Text |  | - **Invite a friend**. Spending time with a supportive walking partner can be a great way to spend time together without interruption. If he or she can't walk with you, make a list of people whose company you enjoy and ask someone from the list. | 2680 |
| EndSelect |  |  | 2690 |
| Text | BarSafePlace>=1 and BarSafePlace<=2 | - **Listen to music or books on tape** to entertain yourself while walking. Just remember to keep the volume low so you can still hear traffic. | 2700 |
| Text | OwnDog=="Yes" | - You have the perfect walking companion - **a dog!** Dogs love walks and you'll love having someone who is constantly ready to go when you are. You know your dog is willing to walk for 10 more minutes. How about you? | 2710 |
| Text | OwnDog=="No" | - **Dogs are great walking companions**. Since you don't own a dog, ask a neighbor or friend if you can take their dog for a walk. Local shelters also appreciate volunteers who walk their dogs. | 2720 |
| Text | containsOne(Employment, ["FullTime", "PartTime"]) | - Get in some of your steps with a co-worker **while on the job**. While waiting for a meeting to start, circle the room and greet others as they arrive. Or, form a workplace walking group and meet to walk together during lunch or a break. | 2730 |
| Text | not containsOne(Employment, ["FullTime", "PartTime"]) | - Get in some of your steps during the day with a neighbor. **Form a neighborhood walking group** and meet to walk together in the morning or early evening. | 2740 |
| Text |  | - **Join a walking club** or an exercise group. Visit your local community center to see what groups or clubs might be available for you to join. | 2750 |
| Text |  | - **Be social**. Dance clubs, hiking groups, health clubs, and adult centers can be great ways to meet new people and find new walking partners. | 2760 |
| Text |  | - **Attend walking events** or walk where there are other walkers. Strike up a conversation as you walk, and soon you will have new walking friends. | 2770 |
| EndBlock |  |  | 2780 |
| Block | Barrier == "BarSelfConscious" |  | 2790 |
| Text |  | **I FEEL SELF CONSCIOUS ABOUT HOW I LOOK WHEN I EXERCISE.** | 2800 |
| Paragraph |  |  | 2810 |
| Text |  | Whether it's the clothes, how your body moves, or being seen by others outside of your usual daily "look", it can be hard to focus on exercising if you feel people are looking at you. Here are some tips to feeling more comfortable in your skin while you walk. | 2820 |
| Text | CurrentSupport=="Yes" | - Ask a friend or family member who you know is supportive of your efforts to walk with you. They might be able to **boost your confidence** and help you get to a place where you feel comfortable about your body while walking.   2830 | |
| Text | CurrentSupport=="No" | - Ask a friend or family member to walk with you. They might be able to **boost your confidence** and help you get to a place where you feel comfortable about your body while walking.   2840 | |
| Text |  | - **Join in a walking event** or walking club. You will see many other people who have bodies similar to yours. | 2850 |
| Text |  | - Buy yourself some brand **new walking clothes**. It can feel great to be in a new outfit. | 2860 |
| Text |  | - Focus on **positive self-talk**. Look in the mirror and tell yourself, "This is important for my health and I really want to do it." | 2870 |
| Text |  | - Remind yourself what a great favor you're doing for your **cardiovascular health**, or focus on how much stronger you feel after a workout. | 2880 |
| Text |  | - An overweight, healthy walker is a **role-model** for so many people who are afraid to be active themselves. You can make a difference in the lives of many others who might see you. | 2890 |
| EndBlock |  |  | 2900 |
