# Supplementary material for: An Online Community Improves Adherence in an Internet-Mediated Walking Program. Part 1: Results of a Randomized Controlled Trial
Source: J Med Internet Res. 2010 Dec 17;12(4):e71. doi: 10.2196/jmir.1338 (PMC3056526; doi:10.2196/jmir.1338)
Supplement: Supplementary file 4 [file jmir_v12i4e71_app4.pdf]

MEDICAL CLEARANCE FORM

I give permission for my physician to provide information to Dr. Richardson's research team to determine my eligibility to participate in a research study.

Participant's signature ________________________________ Date _____/______/______

Dear Dr. _______________________________ (please print full name),

Your patient, ___________________________, would like to participate in our research study for patients with type 2 diabetes and/or coronary artery disease and/or who are overweight or obese.

The study is a 16-week web- and e-mail-based intervention using enhanced pedometers to monitor step-counts and promote walking. The intervention is designed for patients with or at risk for chronic disease. The walking program will promote a gradual increase in walking over the course of 16 weeks. Disease-specific education about walking safely is provided.

Because this patient has diabetes, coronary artery disease, or is at risk for such diseases, and because this walking program is part of a research study, we need to ensure that it is safe for your patient to participate. For most people with diabetes or coronary artery disease or BMIs higher than 25, the risks of remaining sedentary far outweigh the risks of starting a walking program. However, for some patients who have unstable angina, who are currently in the process of a cardiovascular disease workup, or who have other medical problems, the risk having a heart attack while walking might outweigh the benefits of starting a walking program. Therefore, we are asking for medical clearance for your patient to be a part of our research study.

Please check all that apply:

__________ This patient has type 2 diabetes.

__________ This patient has coronary artery disease.

__________ This patient has a BMI of 25 or higher.

Please check one of the following:

___ There is an acceptable level of risk for this patient to begin a walking program.

___ This patient is **not** an appropriate candidate for a walking program.

___ I would like to complete an evaluation before giving medical clearance.

___ I am not familiar with this patient's medical history.

___ Other ____

MD signature ______________________________________ Date _____/______/______

Please fax this completed form to (734) 998-7335, attention: Adrienne Janney.
For questions about the study please contact Dr. Caroline Richardson at (734) 998-7120.
IRBMED HUM00012230
